# A population-based urinary and plasma metabolomics study of environmental exposure to cadmium

**DOI:** 10.1265/ehpm.23-00218

**Published:** 2024-03-30

**Authors:** Yoshiki Ishibashi, Sei Harada, Yoko Eitaki, Ayako Kurihara, Suzuka Kato, Kazuyo Kuwabara, Miho Iida, Aya Hirata, Mizuki Sata, Minako Matsumoto, Takuma Shibuki, Tomonori Okamura, Daisuke Sugiyama, Asako Sato, Kaori Amano, Akiyoshi Hirayama, Masahiro Sugimoto, Tomoyoshi Soga, Masaru Tomita, Toru Takebayashi

**Affiliations:** 1 Department of Preventive Medicine and Public Health, Keio University School of Medicine, Tokyo, Japan; 2 Institute for Advanced Biosciences, Keio University, Tsuruoka, Yamagata, Japan; 3 Faculty of Nursing and Medical Care, Keio University, Fujisawa, Kanagawa, Japan; 4 Faculty of Environment and Information Studies, Keio University, Fujisawa, Kanagawa, Japan

**Keywords:** Metabolomics, Cadmium, Urine, Plasma, Multivariable regression model

## Abstract

**Background:**

The application of metabolomics-based profiles in environmental epidemiological studies is a promising approach to refine the process of health risk assessment. We aimed to identify potential metabolomics-based profiles in urine and plasma for the detection of relatively low-level cadmium (Cd) exposure in large population-based studies.

**Method:**

We analyzed 123 urinary metabolites and 94 plasma metabolites detected in fasting urine and plasma samples collected from 1,412 men and 2,022 women involved in the Tsuruoka Metabolomics Cohort Study. Regression analysis was performed for urinary N-acetyl-beta-D-glucosaminidase (NAG), plasma, and urinary metabolites as dependent variables, and urinary Cd (U-Cd, quartile) as an independent variable. The multivariable regression model included age, gender, systolic blood pressure, smoking, rice intake, BMI, glycated hemoglobin, low-density lipoprotein cholesterol, alcohol consumption, physical activity, educational history, dietary energy intake, urinary Na/K ratio, and uric acid. Pathway-network analysis was carried out to visualize the metabolite networks linked to Cd exposure.

**Result:**

Urinary NAG was positively associated with U-Cd, but not at lower concentrations (Q2). Among urinary metabolites in the total population, 45 metabolites showed associations with U-Cd in the unadjusted and adjusted models after adjusting for the multiplicity of comparison with FDR. There were 12 urinary metabolites which showed consistent associations between Cd exposure from Q2 to Q4. Among plasma metabolites, six cations and one anion were positively associated with U-Cd, whereas alanine, creatinine, and isoleucine were negatively associated with U-Cd. Our results were robust by statistical adjustment of various confounders. Pathway-network analysis revealed metabolites and upstream regulator changes associated with mitochondria (ACACB, UCP2, and metabolites related to the TCA cycle).

**Conclusion:**

These results suggested that U-Cd was associated with metabolites related to upstream mitochondrial dysfunction in a dose-dependent manner. Our data will help develop environmental Cd exposure profiles for human populations.

**Supplementary information:**

The online version contains supplementary material available at https://doi.org/10.1265/ehpm.23-00218.

## 1. Introduction

The application of metabolomics-based profiling in environmental epidemiology is a promising approach to refine the process of health risk assessment of environmental chemicals. Mechanism-based metabolomics profiles reveal biologically plausible exposure-response relationships and link exposure and/or early response with critical health endpoints [1–3]. Metabolomics allows the study of multiple processes among the three domains of the exposome (general external, specific, and internal); thus, it helps to elucidate the impact of toxic chemical exposure on human health, clarify the related integrative biological mechanisms, and identify upstream markers for early detection [4–9].

Cadmium is a nephrotoxic chemical found in air, food, water, and particularly in tobacco smoke [10–14]. Urinary N-acetyl-beta-D-glucosaminidase (NAG) leakage is one of the most sensitive clinical marker of renal tubular damage, occurring at a lower level of Cd exposure than the exposure level of β2-microglobulin and α1-microglobulin [10, 12, 15–17]. NAG is, however, excreted into urine at a relatively late stage of Cd-induced damage [18–20]. Thus, the health risk assessments need refinement because of the potential risks at low levels of environmental Cd exposure.

Some previous studies have examined the association between Cd and urinary metabolites [21–24], but none of those reported their results with adjustment for potential confounding factors sufficiently such as dietary energy intake, alcohol consumption, smoking, blood pressure, and blood glucose levels, probably due to insufficient sample size at low Cd levels. Reliability of metabolite measurement for thousands of samples over the long periods is another issue for a large-scale study as technical variability such as large inter-batch variations can introduce measurement error and bias [25, 26].

We thus conducted a large population-based study with well-standardized metabolomics using capillary electrophoresis time-of-flight mass spectrometry (CE-TOFMS) aimed at identifying changes in metabolomic profiles at relatively low Cd exposure level.

## 2. Methods

### Study population and sample collection

The study population consisted of residents in Tsuruoka City, Yamagata Prefecture, where is a typical Japanese rural area and no history of specific Cd pollution. Therefore, the source of residents’ Cd intake was estimated to be from food and smoking mainly. The Tsuruoka Metabolomics Cohort Study (TMCS) is a population-based cohort study initiated in April 2012 involving 11,002 participants of 35–74 years in age who attended annual municipal or workplace health checkup programs held at four city sites during the baseline period (April 2012–March 2015). TMCS was designed to discover metabolomic biomarkers for diseases and disorders related to environmental and genetic factors as described previously [27–30]. In the present study, first-year participants (4,237 participants) were included for Cd quantification in urine. Individuals that did not respond to smoking habit questions; had experienced a stroke, or been diagnosed with ischemic heart disease, cancer, or liver disease; had non-fasting urine and plasma samples; and/or had no urine and plasma samples were excluded from the study (S Fig. 1). Consequently, 1,412 men and 2,022 women were included in the statistical analysis. This study is a cross-sectional study and analyzed baseline participants.

All participants completed a comprehensive questionnaire on lifestyle, dietary habits, and medical history. We also collected biological samples, including serum, urine, plasma, and deoxyribonucleic acid (DNA), along with medical examination data recorded during the health checkup programs at recruitment. Information on drinking and smoking habits, dietary patterns, and level of physical activity was obtained through a structured, self-administered questionnaire, and these data were screened by trained staff using face-to-face interviews. Alcohol intake per week was calculated based on the frequency of alcohol consumption during a typical week and the total alcohol intake on each occasion, which was then divided by seven to obtain the average alcohol intake per day [31]. Smokers were classified as current, past, or non-smokers, and pack-years were studied [32]. Food Frequency Questionnaire (FFQ) and Physical Activity Questionnaire (PAQ) were used to estimate the dietary intake and physical activity of the population [33–35]. To avoid variation due to fasting state and circadian rhythm, urine and plasma samples were collected from each participant at 8:30–10:30 am after an overnight fast. Blood pressure was measured twice in the sitting position using an Omron HBP-T105S-N sphygmomanometer (Omron, Kyoto, Japan), and the mean of the two measurements was used for analysis. Serum levels of cholesterol were analyzed using enzymatic methods; glycated hemoglobin (HbA1c) was determined by immunoassay; and low-density lipoprotein cholesterol (LDL-C) levels were calculated using the Friedwald equation.

### Metabolomic measurements

Non-targeted mass spectrometry-based metabolomic profiling was performed using urine and plasma samples with capillary electrophoresis time-of-flight mass spectrometry (CE-TOFMS) as described previously [36–39]. Raw data were analyzed with our proprietary software called MasterHands, which has already been used in several CE-TOF-MS-based profiling studies [40–42]. The data analysis workflow starting with the raw data, included noise-filtering, baseline correction, peak detection, and integration of the peak area from sliced electropherograms. Based on the preliminary investigation described above non-target profiling, we decided to routinely measure the absolute concentrations of 123 urinary metabolites (69 cations and 54 anions) and 94 plasma metabolites (54 cations and 40 anions) that are expected to be consistently measured in most human samples and match the standards in the cohort. These urine and plasma metabolite measurements have been validated for measurement reliability in large-scale epidemiology studies [29, 30].

### Quantification of U-Cd and NAG

The U-Cd concentration was determined using atomic absorption spectrophotometry (graphite furnace atomic absorption spectrophotometer, Z-2710, Hitachi High-Tech Science Corporation, Japan). No pretreatment was performed, and samples of urine diluted in an aqueous solution containing a palladium matrix modifier were submitted to the AAS machine, and subsequently dried and ashed on the graphite furnace of the instrument. The Cd standard was purchased from Fujifilm Wako Pure Chemicals Co., Ltd. (100 mg/L, Fujifilm Wako Pure Chemical Co., Osaka, Japan). Palladium matrix modifier (for atomic absorption spectrometry, grade for atomic absorption spectrophotometer, Kanto Chemical Co., Tokyo, Japan) and nitric acid (ultrapure grade, Kanto Chemical Co., Tokyo, Japan) were purchased from Kanto Chemical Co. Ultrapure water purified by a pure water production system (Direct-Q UV, Merck Ltd., Tokyo, Japan) was used for pretreatment and analysis. Two measurements were conducted, and those with a relative standard deviation (RSD) of 15% or more were remeasured. The average of two measurements was used for analysis. For external quality control, we used the German external quality assessment scheme for analyses in biological materials (G-EQUAS) sponsored by the Institute and Out-Patient Clinic for Occupational, Social and Environmental Medicine of the University Erlangen-Nuremberg. We obtained a + rating at the environmental and occupational levels under the scheme. For internal precision control, Seronorm Trace Elements Urine (SERO AS, Billingstad, Norway) and Lyphochek Urine Metals Control (Bio-Rad Laboratories, CA, US) were used to check the accuracy of each analytical lot. The concentration of NAG in the urine was determined using a commercial clinical chemistry laboratory test (Colorimetric method; SRL, Tachikawa, Tokyo, Japan).

### Statistical analysis

U-Cd concentration was adjusted for urinary creatinine and classified into quartiles, and the effect of Q2–Q4 in U-Cd was evaluated using Q1 as a reference. NAG was handled as a continuous variable, and its concentration was adjusted for urinary creatinine. First, to clarify Cd exposure-related changes in NAG in this population, we carried out single and multiple regression analysis with NAG (IU g^−1^ cre) as a dependent variable and the quartiles of U-Cd as an independent variable (unadjusted model; ANOVA by general linear model).

Then, to explore the exposure-related change in urinary and plasma metabolites, single and multiple regression analysis was performed for 123 urine metabolites (69 cations and 54 anions) and 94 plasma metabolites (54 cations and 40 anions) as dependent variables and U-Cd (quartile) as an independent variable. Covariates included in the multiple regression analysis were age (years), sex, systolic blood pressure (SBP; mmHg), smoking (packs yr^−1^), rice intake (bowls wk^−1^), body mass index (BMI; kg m^−2^), HbA1c (%), LDL-C (mg dl^−1^), alcohol consumption (ethanol intake, g d^−1^), metabolic equivalent for tasks (METs, quartiles), educational history (<10 years, 10–12 years, and >12 years), dietary energy intake (kcal d^−1^ quartiles), urinary Na/K ratio, and uric acid (mg dl^−1^) (adjusted model; ANCOVA by general linear model). Since missing values for metabolites were created by being less than the measurement limit, values of half of the lowest detected values were input for those not detected [28, 30]. The regression equation is described in the following form.

Y=aX+b, Y=NAG or Metabolites, X=U-Cd

For sensitivity analysis, stratification with gender or age (≥65 years old or below) was done. We then excluded ex-smokers and current smokers to evaluate the effect of Cd exposure among non-smokers. We also excluded participants with estimated glomerular filtration rate (eGFR) <60 ml min^−1^/1.73 m^2^, those with HbA1c >6.5%, those who prescribed diabetes medications, and those with extremely high/low urinary creatinine concentration (>3.0 g L^−1^ or <0.3 g L^−1^). To confirm the results of main analysis, additional analysis with continuous U-Cd as an independent variable was performed. To adjust for multiple comparison, we calculated p values adjusted using the Benjamini–Hochberg procedure for decreasing the false discovery rate (FDR) and screened with the Jonckheere–Terpstra test at the 5% level.

Urinary metabolite levels were adjusted for creatinine levels. Urinary and plasma metabolites were log-transformed and normalized. A mixed model was selected to exclude the effects of batch measurements [43]. All statistical analyses were performed using R 3.5.2 (R Core Team 2018).

### Pathway-network analysis

We used Ingenuity Pathway Analysis (IPA) to visualize the metabolite networks linked to Cd exposure [44]. We uploaded the list of coefficient and p values for metabolites for multivariate analysis in the total study population into IPA and set the cutoff at *p* < 0.05. IPA generated a shortlist of interaction networks around the metabolites of interest merged into a single combined network image (network analysis). The upstream regulators and proteins were predicted from observed urinary metabolites (upstream analysis).

### Ethical approval

This study was approved by the Medical Ethics Committee of the School of Medicine, Keio University, Tokyo, Japan (approval no. 20110264). Written informed consent was obtained from all the study participants. All studies were performed according to relevant guidelines and regulations.

## 3. Results

Table 1 shows the characteristics of the study participants according to the quartiles of Cd exposure. Mean U-Cd concentration in total population was 2.65 ± 1.63 µg g^−1^) (Table 1). The characteristics for men and women were shown in S Table 1. Average U-Cd was 1.87 ± 1.04 µg g^−1^, and range of each quartiles were 0.10–1.13 µg g^−1^ (Q1), 1.13–1.68 µg g^−1^ (Q2), 1.68–2.33 µg g^−1^ (Q3) and 2.33–8.54 µg g^−1^ (Q4) for men, and those for women were 3.20 ± 1.74 µg g^−1^ (average), 0.13–2.04 µg g^−1^ (Q1), 2.04–2.90 µg g^−1^ (Q2); 2.90–4.08 µg g^−1^ (Q3) and 4.08–25.62 µg g^−1^ (Q4). Urinary Cd concentration in this population is in the same range as in other non-polluted areas in Japan [45, 46]. Cd exposure increased with age, NAG, rice-intake, and pack-year (whose variables are considered to be associated with Cd) in total population and in each sex. Other variables such as energy intake, HbA1c, LDL-C, and education history also differed between Cd exposure levels in total population and sex stratification (S Table 1). A positive association between Cd exposure and urinary NAG was observed in the multivariate regression analysis in total population. Adjusted difference (95%CI) of NAG compared to Q1 were 0.14 (−0.35, 0.63) µg/g creatinine for Q2, 0.81 (0.29, 1.32) for Q3 and 1.05 (0.52, 1.59) for Q4 (trend p < 0.001), indicating that urinary NAG concentrations increased from Q3. Those for men were −0.07 (−0.90, 0.76) for Q2, 0.27 (−0.60, 1.15) for Q3, and 2.03 (1.11, 2.96) for Q4 (trend p < 0.001), and those for women were 0.13 (−0.48, 0.73), 0.34 (−0.27, 0.95), and 0.95 (0.34, 1.57) (trend p = 0.001), respectively. In U-Cd below 2.31 µg/g Cre (= Q2) in total population and below 1.68 µg/g Cre (= Q2) in men and 2.90 µg/g Cre (= Q2) in women, there is not elevated in NAG.

**Table 1:** Characteristics in the total study population

**Variable**	**Total**				**P value**
**Urinary cadmium (U-Cd), Quartiles**	**Q1 (n = 859)**	**Q2 (n = 858)**	**Q3 (n = 858)**	**Q4 (n = 859)**	
U-Cd (µg/g cre)^a^	1.07 [0.10, 1.49]	1.92 [1.49, 2.31]	2.79 [2.31, 3.44]	4.47 [3.44, 25.62]	<0.001
NAG (IU/g cre)^b^	4.30 (3.67)	5.00 (3.73)	5.65 (5.07)	5.95 (4.99)	<0.001
eGFR (mL/min/1.73 m^2^)^b^	74.5 (13.3)	73.6 (12.7)	73.2 (12.3)	74.0 (12.0)	<0.001
Sex (male)^c^	596 (69.3)	450 (52.4)	30.4 (30.4)	105 (12.2)	<0.001
Age (years)^b^	55.9 (10.5)	61.7 (7.9)	63.2 (6.9)	64.1 (6.1)	<0.001
BMI (kg/m^2^)^b^	23.7 (3.4)	23.2 (3.2)	23.2 (3.1)	22.8 (3.4)	<0.001
Smoking (Yes)^c^	190 (22.1)	142 (16.6)	101 (11.8)	59 (6.9)	<0.001
Ex-smoking (Yes)^c^	326 (38.0)	269 (31.3)	167 (19.5)	85 (9.9)	<0.001
Pack year (only smoker)^b^	24.4 (17.7)	29.8 (19.1)	30.1 (20.9)	29.3 (22.0)	<0.001
Rice intake (bowl/week)^b^	18.4 (6.4)	18.6 (5.6)	18.7 (5.6)	19.0 (5.3)	0.004
Energy intake (kcal/day)^b^	1857 (435.1)	1827 (404.6)	1757 (394.2)	1672 (341.8)	<0.001
Ethanol intake (g/day)^b^	21.6 (28.8)	18.0 (26.8)	11.9 (23.8)	6.3 (16.7)	<0.001
Physical activity (METs·hour/week)^b^	26.6 (14.9)	27.3 (14.9)	27.5 (15.5)	26.7 (13.2)	0.353
SBP (mmHg)^b^	129.4 (19.0)	130.3 (19.0)	130.5 (19.5)	129.3 (20.0)	0.313
HbgA1c (%)^b^	5.66 (0.65)	5.71 (0.57)	5.70 (0.54)	5.70 (0.55)	<0.001
LDL-C (mg/dL)^b^	142.7 (34.2)	140.2 (34.4)	143.5 (31.6)	141.0 (31.2)	0.113
Urea acid (mg/dL)^b^	5.55 (1.44)	5.19 (1.32)	4.89 (1.27)	4.64 (1.13)	<0.001
Urinary Na/K ratio^b^	2.81 (1.61)	2.88 (1.55)	2.79 (1.44)	3.01 (1.50)	0.002
Education (years)^c^					
Less than or equal to 9 years (Yes)	102 (11.9)	163 (19.0)	194 (22.6)	234 (27.2)	<0.001
10 to 12 years (Yes)	476 (55.4)	478 (55.7)	478 (55.7)	467 (54.4)	0.919
More than 12 years (Yes)	276 (32.1)	215 (25.0)	178 (20.7)	164 (19.1)	<0.001

eGFR estimated glomerular filtration rate, BMI Body mass index, SBP systolic blood pressure, HbA1c Hemoglobin A1c, LDL-C low-density lipoprotein cholesterol, METs Metabolic equivalent for tasks.

^a^Reported as median (range)

^b^Reported as mean (standard deviation)

^c^Percent and numbers are shown

Figure 1 shows the association between Cd exposure and urine metabolites in the multivariate regression model (adjusted model) in the total population. Among urinary metabolites in the total population, 45 metabolites (19 cations; trimethylamine N-oxide, alanine [Ala], choline, taurine, 1-methylnicotinamide, asymmetric dimethylarginine [ADMA], uridine, adenosine, ethanolamine, piperidine, 5-aminolevulinate, allantoin, 7-methylguanine, N1-acetylspermidine, N8-acetylspermidine, Glycyl-L-leucine [Gly-Leu], N-epsilon-acetyllysine, S-Adenosylmethionine [SAM+], and 1-methyladenosine and 26 anions; lactate, malonate, 4-oxopentanoate, succinate, isethionate, 5-oxoproline, glutarate, malate, threonate, ethanolamine phosphate, 2-oxoglutarate, pimelate, urate, glycerophosphate, trans-aconitate, cis-aconitate, N-acetylaspartate, azelate, isocitrate, adipate, 3-hydroxy-3-methylglutarate, 2,3-pyridinedicarboxylate, suberate, gluconate, saccharate, and N-acetylneuraminate) showed associations with U-Cd in the unadjusted and adjusted models after adjusting for the multiplicity of comparison with FDR. There were 12 urinary metabolites (alanine, uridine, ethanolamine, piperidine, lactate, 5-oxoproline, ethanolamine phosphate, 2-oxoglutarate, glycerophosphate, cis-aconitate, azelate, and isocitrate) which showed consistent associations between Cd exposure from Q2 to Q4. All urinary metabolites were positively associated with Cd exposure, except for Ala and piperidine, which were negatively associated with Cd exposure. After gender stratification, 8 metabolites had consistently positive associations; Uridine, 5-Aminolvulinate, 1-Methyladenosine, Isethionate, Isethionate, Urate, Isocitrate and Suberate (S Fig. 2). The result by univariate model is shown in S Fig. 3. Statistical summary of results of the multivariate regression model between U-Cd and all urine metabolites (123 metabolites) was exhibited in S Table 2. The result by using a variable of continuous U-Cd is shown in S Table 3.

**Fig. 1A:**
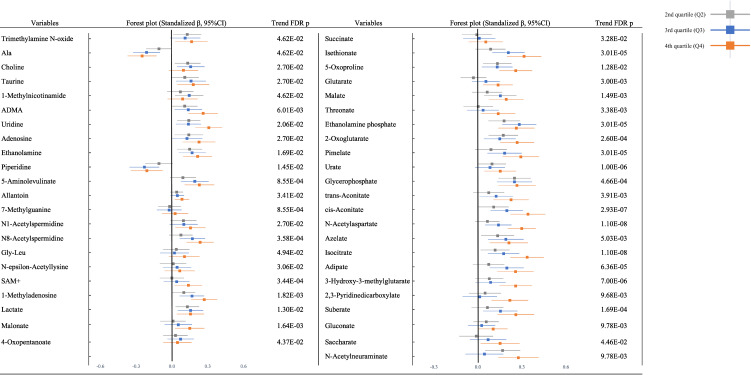
Association between urine metabolites and cadmium exposure in total population. (Standalized β, Q2–Q4 compared to Q1). Multivariate regression model was adjusted by age (years), sex, systolic blood pressure (mmHg), smoke (pack-year), rice intake (bowl/week), BMI (kg/m2), HbA1c (%), LDLc (mg/dL), alcohol consumption (ethanol intake: g/day), physical activity (METs, quartiles), educational history (less than 10 years, 10 to 12 years, more than 12 years), dietary energy intake (kcal, quartiles), urinary Na/K ratio and uric acid (mg/dL). Trend p values were adjusted by FDR (False Discovery Rate) in each qurtile (123 penalties per analysis).

**Fig. 1B:**
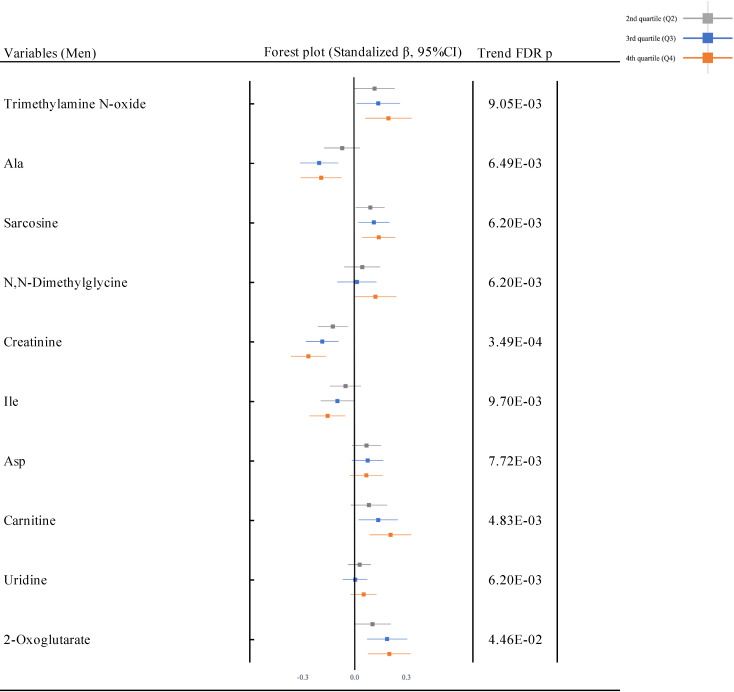
Association between plasma metabolites and cadmium exposure in total population. (Standalized β, Q2–Q4 compared to Q1). Multivariate regression model was adjusted by age (years), sex, systolic blood pressure (mmHg), smoke (brinkman index), rice intake (bowl/week), BMI (kg/m2), HbA1c (%), LDLc (mg/dL), alcohol consumption (ethanol intake: g/day), physical activity (METs, quartiles), educational history (less than 10 years, 10 to 12 years, more than 12 years), dietary energy intake (kcal, quartiles), urinary Na/K ratio and uric acid (mg/dL). P values were adjusted by FDR (False Discovery Rate) in each quartile (94 penalties per analysis).

Figure 2 shows the forest plots for the association between Cd exposure and plasma metabolites in the multivariate regression model (adjusted model) in the total population. Among the plasma metabolites in the total population, 10 metabolites (9 cations; trimethylamine *N*-oxide, Ala, sarcosine, *N*,*N*-dimethylglycine, creatinine, isoleucine [Ile], aspartic acid [Asp], carnitine, and uridine, and 1 anion; 2-oxoglutarate) were associated with U-Cd in the unadjusted and adjusted models after adjusting for the multiplicity of comparison with FDR. All plasma metabolites were positively associated with U-Cd, except for Ala, creatinine, and Ile, which were negatively associated with U-Cd. Similar patterns were observed after gender stratification (S Fig. 4). The univariate model is shown in S Fig. 5. Statistical summary of results of the multivariate regression model between U-Cd and all plasma metabolites (94 metabolites) was exhibited in S Table 4. The result by using a variable of continuous U-Cd is shown in S Table 5.

**Fig. 2A:**
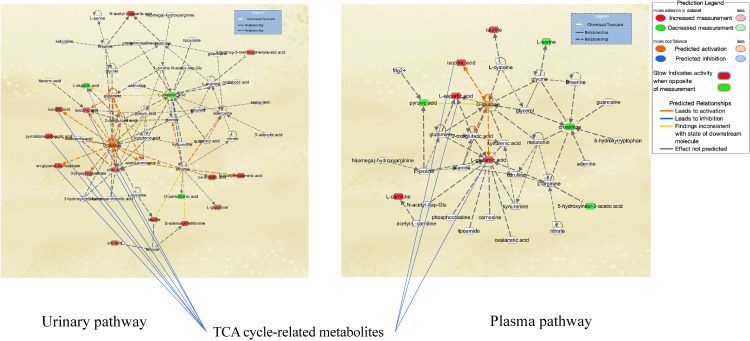
Urinary and plasma metabolic network characterizing cadmium exposure. Metabolites highlighted in strong colors passed the testing in p < 0.05 and were replicated for multivariate model (in Q4). Red means increased measurement, and Green means decreased measurement. Orange is predicted activation and Blue is predicted inhibition. Mammalian endogenous chemicals were extracted.

**Fig. 2B:**
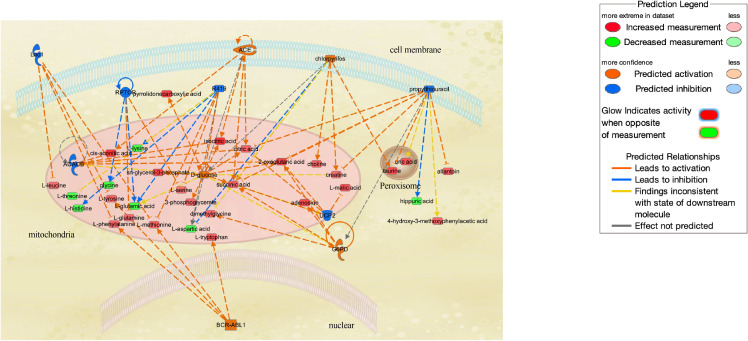
Upstream regulator and protein for observed urinary metabolites. Metabolites highlighted in strong colors passed the testing p < 0.05 and were replicated for multivariate (in Q4). Red means increased measurement, and Green means decreased measurement. Orange is predicted activation and Blue is predicted inhibition. Genes, RNAs and proteins were extracted. Chemical drugs and ligands were excluded.

Pathway-network analysis illustrated the biological relationships among metabolites due to Cd exposure (Figs. 2A and 2B). Network analysis revealed tricarboxylic acid (TCA) cycle-related metabolites and the association of Cd exposure with glucose metabolism in the urine and plasma (Fig. 2A). Analysis of upstream regulators and proteins showed changes in the pathways for ACACB, ACE, BCR-ABL1, G6PD, Lao1, RPTOR, and UCP2 in urinary metabolites (Fig. 2B). Pathway-network analysis displayed metabolites and regulator changes associated with mitochondrial disorders (ACACB, UCP2, and metabolites related to the TCA cycle).

The results of the sensitivity analysis showed a similar trend as that of the main analysis with ex-smokers and current smokers excluded (S Fig. 6–10); age-stratified participants, ≥65 years old or not; and high NAG and U-Cd excluded in the total population, men and women (data not shown). The increasing pattern in urinary metabolites (except for Ala and Piperidine) and the pattern in plasma metabolites did not change, maintaining their robustness. Other sensitivity analyses (excluding participants with urinary creatinine >3.0 g L^−1^ or <0.3 g L^−1^; excluding participants with estimated glomerular filtration rate (eGFR) <60 ml min^−1^/1.73 m^2^; excluding participants with HbA1c >6.5 or prescribed diabetes medications; excluding alcohol consumers were also not different from the main results (S Fig. 11–30)). The association of Cd exposure with urinary metabolites and plasma metabolites did not change even when ex-smokers and current smokers were excluded from the analysis, suggesting that Cd and metabolites may be related to pathways other than smoking (S Fig. 6–10).

## 4. Discussion

In this study where urinary NAG concentrations increased in the half of the study population (Q3 and Q4 of urinary Cd) within normal eGFR level, 45 urinary metabolites were significantly changed even after adjustment for potential confounders, and 12 urinary metabolites (alanine, uridine, ethanolamine, piperidine, lactate, 5-oxoproline, ethanolamine phosphate, 2-oxoglutarate, glycerophosphate, cis-aconitate, azelate, and isocitrate) showed consistent associations between Cd exposure from Q2 to Q4. According to the Human Metabolome Database [47], 7 out of 12 (alanine, uridine, lactate, 2-oxoglutarate, glycerophosphate, cis-aconitate, and isocitrate) are suggested to be mitochondrial metabolites. Pathway-network analysis of urinary metabolites (Fig. 2B) indicated potential changes in ACACB and UCP2, two mitochondrial proteins expressed in human renal proximal tubular epithelial cells. *In vitro* studies showed that ACACB controls the rate-limiting step in fatty acid uptake and oxidation by mitochondria, whereas its suppression rescues human proximal tubular cells from Cd-induced lipotoxicity via autophagy [48], and UCP2, an anion transporter, modulates the production of mitochondrial reactive oxygen species (ROS) and plays an essential role in protecting against proximal tubular cell apoptosis and mitochondrial respiratory function [49]. Thus, exposure-related changes in urinary metabolites we observed might reflect early signs of mitochondrial dysfunction due to Cd-induced tubular damage.

So far, two studies indicated the change in urinary metabolites with relation to Cd exposure [21, 24]. Ellis et al. studied with 180 members of the general UK population with low U-Cd concentrations (0.22 µg/g creatinine and 0.34 µg/g creatinine in men and women, respectively), and found that urinary citric acid measured by NMR was associated with U-Cd even after controlling for age, gender, and smoking status. Change in citric acid at lower U-Cd exposure levels is consistent with our results and suggest the potential occurrence of mitochondrial damage. Xu et al. examined in 33 relatively high Cd-exposed women (U-Cd µg/g cre >15, 5–15, <5) living in China, and observed significant the relationship between urinary Cd and urinary metabolites determined by GC-MS including 8 small molecular carbohydrates, 2 amino acids, 2 fatty acids, and 9 metabolic intermediates, including polyol, glycoside sugar acid, and aldehyde acid. The metabolic pathways identified were a variety of carbohydrate metabolism pathways such as the sorbitol pathway, ribose metabolism, galactose metabolism, the transformation of pentose to glucuronide, amino acid metabolism, and the tricarboxylic acid (TCA) cycle, which were consistent with the present study in identifying effects on amino acid metabolism, TCA cycle, etc.

The European Chemicals Agency proposed the application of metabolomics into risk assessment methods (European Chemicals Agency, 2016.). The MEtabolomics standaRds Initiative in Toxicology (MERIT) project reported [50] that the standardization for procedures, measurements and analysis of metabolomics such as defining the chemical exposures and the use of positive biological controls, sample size, the design number of sampling times, randomisation and batching of samples, and ways of QC samples is needed. From the epidemiological viewpoint [51], (1) a larger sample size is necessary to sufficiently adjust for confounders, and (2) QC measurement for metabolites in a large cohort was reported to be essential to reduce random errors and increase accuracy. In the present study, we performed statistical analysis with adjustment for potential important confounders (dietary energy intake, alcohol consumption, smoking, blood pressure, and blood glucose levels) or by various sensitivity analysis with sufficient sample size. Furthermore, it is noteworthy that measurement of urine and plasma metabolites were done with valid QC protocol throughout the assay [29, 30]. Furthermore, CE-MS can identify and quantify a wide range of metabolites [36–39]. CE-MS has been successfully used in numerous clinical applications in recent years compared to other platforms. It is because CE-MS lies in its higher quantification accuracy than other methods like NMR, LC-MS, and HPLC and in the availability of large comparable datasets that were all obtained using the same routine procedure for sample preparation, analysis, and subsequent data evaluation [52–56].

Cadmium exposure in the Japanese population occurs mainly through smoking and rice consumption [57]. Smoking rates have been declining in recent years in Japan [58], but continued efforts to reduce smoking rates are needed. In the case of Japan, rice is the main problem. This is often a problem due to the soil, and since the government is working on soil replacement initiatives [59], it is important to continue these efforts.

Another strength of this study is that plasma metabolites were quantified in the same population at the same time point. Ten plasma metabolites were associated with urinary Cd, and 90% of those are mitochondrial metabolites. A systematic review of clinical applications of metabolomics points out that using multiple biological fluids to analyze metabolic profiles provides greater insight into etiology [60]. In the present study, Cd exposure was negatively associated with plasma concentrations of Ala, creatinine, and Ile, which was reported that a leakage marker of glomeruli damage and hyperfiltration [61, 62]. In this study, we showed that leakage occurs to the extent that elevation of NAG does not occur, and there is the possibility that glomerular hyperfiltration and decrease of nephrons occur because of the decrease in plasma metabolites, creatinine and amino acids, and the broad elevation of urinary metabolites. Cd exposure has been suggested only for tubular disorders, but recent *in vivo* and *in vitro* studies have also focused on its effects on glomeruli at early, low concentrations exposure [63, 64]. Thus, combined analysis of urinary and plasma metabolites could lead to better description of toxicological profiles of Cd in humans with deeper mechanistic understanding.

Our study had some limitation, naturally. Since this is a cross-sectional study, reverse causality might be inevitable. Because of long biological half-life of Cd in urine, i.e. 10–30 years, urinary Cd is affected by past exposure, and there was a possibility that the changes in metabolites might reflect recent lifestyle factors such as smoking and food consumption independent of Cd. We thus performed various sensitivity and multivariate-adjusted analysis to confirm the results. We also did not include unmeasured confounding factors including other environmental and occupational chemicals. In this population, there are no obvious renal dysfunction has occurred, however, since urinary NAG is elevated, renal tubular dysfunction has begun, and renal dysfunction is expected to occur in the future. Follow-up investigation is needed for osteoporosis, cardiac disease and etc. in higher exposure population. Another follow-up study is also needed and on-going to evaluate how metabolomics can contribute to refine environmental health risk assessment methods.

## Conclusion

Our large-scale epidemiological study with well-standardized metabolomics in urine and plasma can detect Cd toxicity using metabolites. The results were robust using statistical adjustment of potential confounders or sensitivity analysis. Metabolomic profiling might detect the effects of Cd exposure on renal dysfunction at an earlier stage than NAG because metabolites in mitochondria sensitively reflect changes in the upstream regulators of tubular disorders.

## Supplementary information

**Additional file 1:** **S** **Figure 1** Overview of participants’ flow.

**Additional file 2:** **S** **Figure 2** Association between urine metabolites and cadmium exposure groups in multivariate regression model in men (Standalized β, Q2–Q4 compared to Q1).

**Additional file 3:** **S Figure 3** Association between urine metabolites and cadmium exposure groups in univariate regression model in total population (Standalized β, Q2–Q4 compared to Q1).

**Additional file 4:** **S Figure 4** Association between plasma metabolites and cadmium exposure groups in multivariate regression model in sex stratification (Standalized β, Q2–Q4 compared to Q1).

**Additional file 5:** **S Figure 5** Association between plasma metabolites and cadmium exposure groups in univariate regression model (Standalized β, Q2–Q4 compared to Q1).

**Additional file 6:** **S Figure 6** Association between urine metabolites and cadmium exposure groups in nonsmoker.

**Additional file 7:** **S Figure 7** Association between urine metabolites and cadmium exposure groups in multvariate regression model in nonsmoker men.

**Additional file 8:** **S Figure 8** Association between urine metabolites and cadmium exposure groups in multvariate regression model in nonsmoker women.

**Additional file 9:** **S Figure 9** Association between plasma metabolites and cadmium exposure groups in nonsmoker.

**Additional file 10:** **S Figure 10** Association between plasma metabolites and cadmium exposure groups in sex stratification.

**Additional file 11:** **S Figure 11** Association between urine metabolites and cadmium exposure groups excluding participants for urinary creatinine >3.0 g/L or <0.3 g/L.

**Additional file 12:** **S Figure 12** Association between urine metabolites and cadmium exposure groups excluding participants for urinary creatinine >3.0 g/L or <0.3 g/L in men.

**Additional file 13:** **S Figure 13** Association between urine metabolites and cadmium exposure groups excluding participants for urinary creatinine >3.0 g/L or <0.3 g/L in women.

**Additional file 14:** **S Figure 14** Association between plasma metabolites and cadmium exposure groups excluding participants for urinary creatinine >3.0 g/L or <0.3 g/L.

**Additional file 15:** **S Figure 15** Association between plasma metabolites and cadmium exposure groups excluding participants for urinary creatinine >3.0 g/L or <0.3 g/L in sex stratification.

**Additional file 16:** **S Figure 16** Association between urine metabolites and cadmium exposure groups excluding participants of eGFR is >60 mL/min/1.73 m2.

**Additional file 17:** **S Figure 17** Association between urine metabolites and cadmium exposure groups excluding participants of eGFR is >60 mL/min/1.73 m2 in men.

**Additional file 18:** **S Figure 18** Association between urine metabolites and cadmium exposure groups excluding participants of eGFR is >60 mL/min/1.73 m2 in women.

**Additional file 19:** **S Figure 19** Association between plasma metabolites and cadmium exposure groups excluding participants of eGFR is >60 mL/min/1.73 m2.

**Additional file 20:** **S Figure 20** Association between urine metabolites and cadmium exposure groups excluding participants of eGFR is >60 mL/min/1.73 m2 in sex stratification.

**Additional file 21:** **S Figure 21** Association between urine metabolites and cadmium exposure groups excluding participants of HbA1c is <6.5% or prescribing diabetes medication.

**Additional file 22:** **S Figure 22** Association between urine metabolites and cadmium exposure groups excluding participants of HbA1c is <6.5% or prescribing diabetes medication in men.

**Additional file 23:** **S Figure 23** Association between urine metabolites and cadmium exposure groups excluding participants of HbA1c is <6.5% or prescribing diabetes medication in women.

**Additional file 24:** **S Figure 24** Association between plasma metabolites and cadmium exposure groups excluding participants of HbA1c is <6.5% or prescribing diabetes medication.

**Additional file 25:** **S Figure 25** Association between urine metabolites and cadmium exposure groups excluding participants of HbA1c is <6.5% or prescribing diabetes medication in sex stratification.

**Additional file 26:** **S Figure 26** Association between urine metabolites and cadmium exposure groups excluding drinkers.

**Additional file 27:** **S Figure 27** Association between urine metabolites and cadmium exposure groups excluding drinkers in men.

**Additional file 28:** **S Figure 28** Association between urine metabolites and cadmium exposure groups excluding drinkers in women.

**Additional file 29:** **S Figure 29** Association between plasma metabolites and cadmium exposure groups excluding drinkers.

**Additional file 30:** **S Figure 30** Association between urine metabolites and cadmium exposure groups excluding drinkers in sex stratification.

**Additional file 31:** **S Table 1** Characteristics in the study population in sex stratification.

**Additional file 32:** **S Table 2** Statistical summary of the association in the multivariate regression model between Cd exposure and urine metabolites. (In total population).

**Additional file 33:** **S Table 3** Statistical summary of the association in the multivariate regression model between continuous U-Cd and urine metabolites. (In total population).

**Additional file 34:** **S Table 4** Statistical summary of the association in the multivariate regression model between Cd exposure and plasma metabolites. (In total population).

**Additional file 35:** **S Table 5** Statistical summary of the association in the multivariate regression model between continuous U-Cd and plasma metabolites. (In total population).

## Declaration

### Availability of data and material

The most relevant data are within the paper. Raw data cannot be made publicly available, as study participants did not consent to have their information freely accessible. Based on these terms of consent, the Ethics Committee for Tsuruoka Metabolomics Cohort Study inhibits any public data sharing because data contain potentially identifying or sensitive disease information. This committee includes representatives of Tsuruoka citizens, the administration of Tsuruoka City, a lawyer, and expert advisers. Data accession requests may be sent to the administration of the Ethics Committee for the Tsuruoka Metabolomics Cohort Study. The data will be shared after a review of the purpose and with permission from the ethics committee. Data requests can be made to Sei Harada, seiharada@keio.jp. The source code and analysis generated during the current study are not publicly available, but are available from the corresponding author upon reasonable request.

### Pre-print

There is a pre-print open work of this paper [65].
