# Supplementary figures and images for: A population-based urinary and plasma metabolomics study of environmental exposure to cadmium
Source: Environ Health Prev Med. 2024 Mar 30;29:22. doi: 10.1265/ehpm.23-00218 (PMC10992994; doi:10.1265/ehpm.23-00218)

# S Figure 1 Overview of participants' flow.

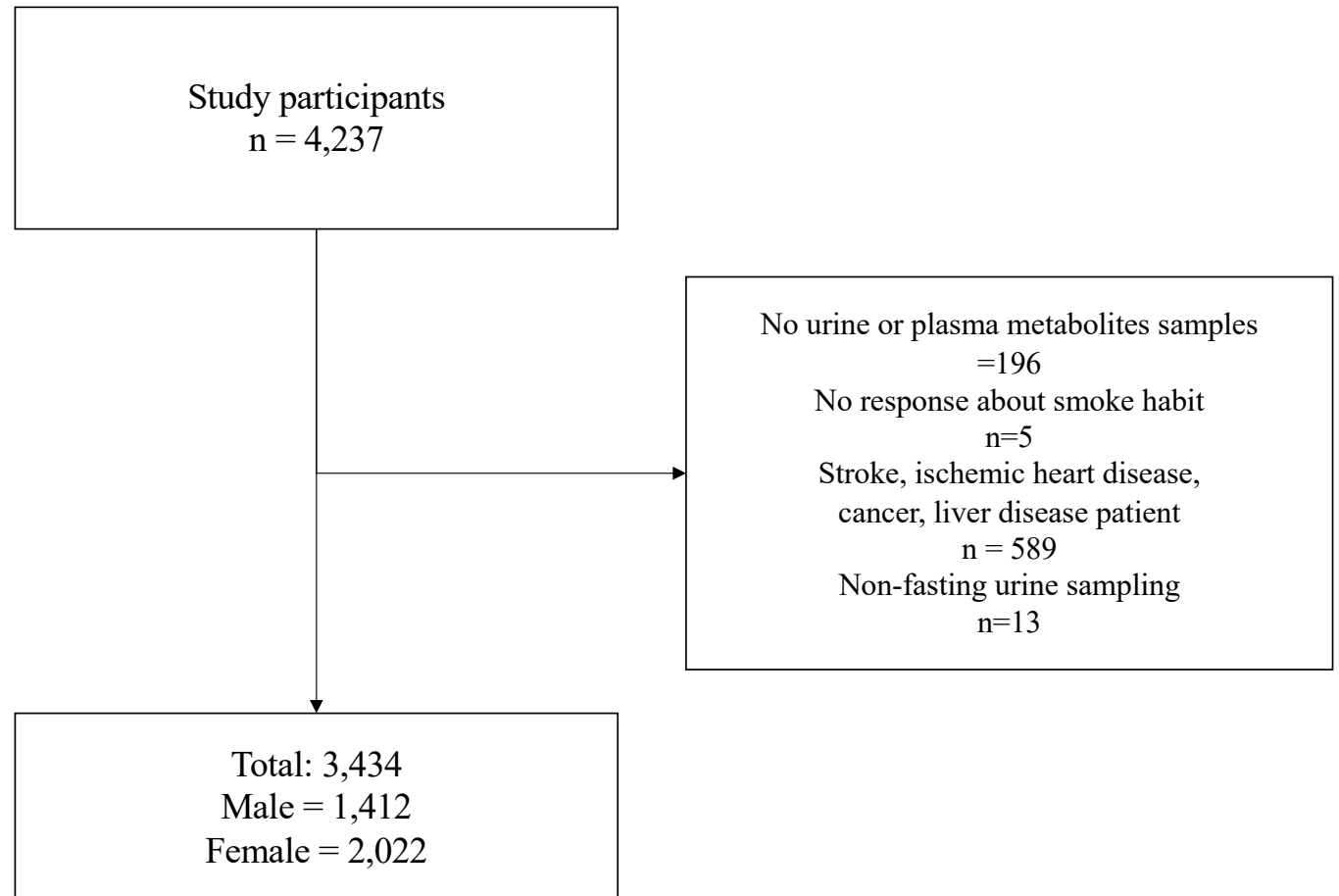

Supplement: Supplementary file 1 — Additional file 1: S Figure 1 Overview of participants’ flow. [file ehpm-29-022-s001.pdf]

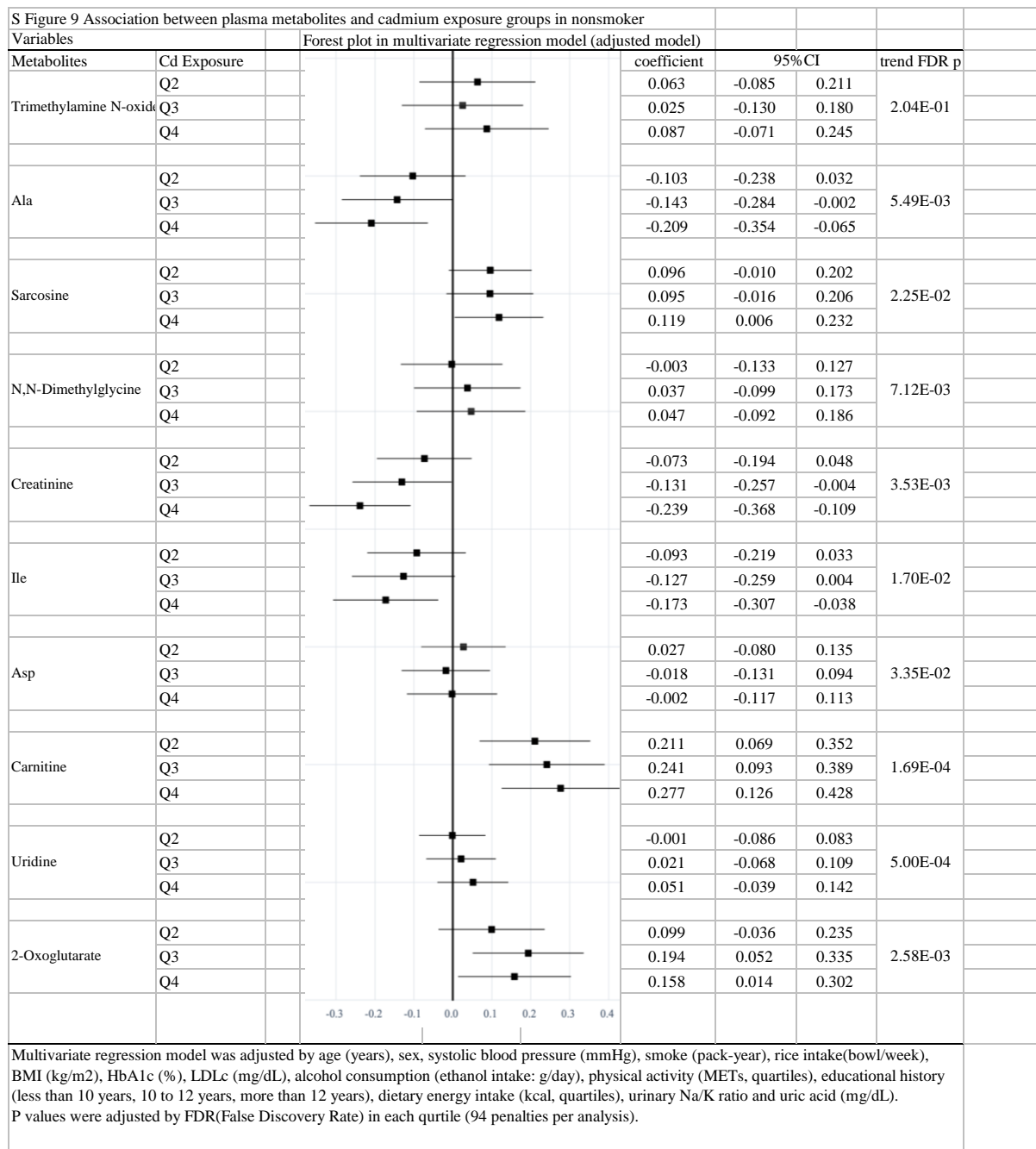

Supplement: Supplementary file 9 — Additional file 9: S Figure 9 Association between plasma metabolites and cadmium exposure groups in nonsmoker. [file ehpm-29-022-s009.pdf]

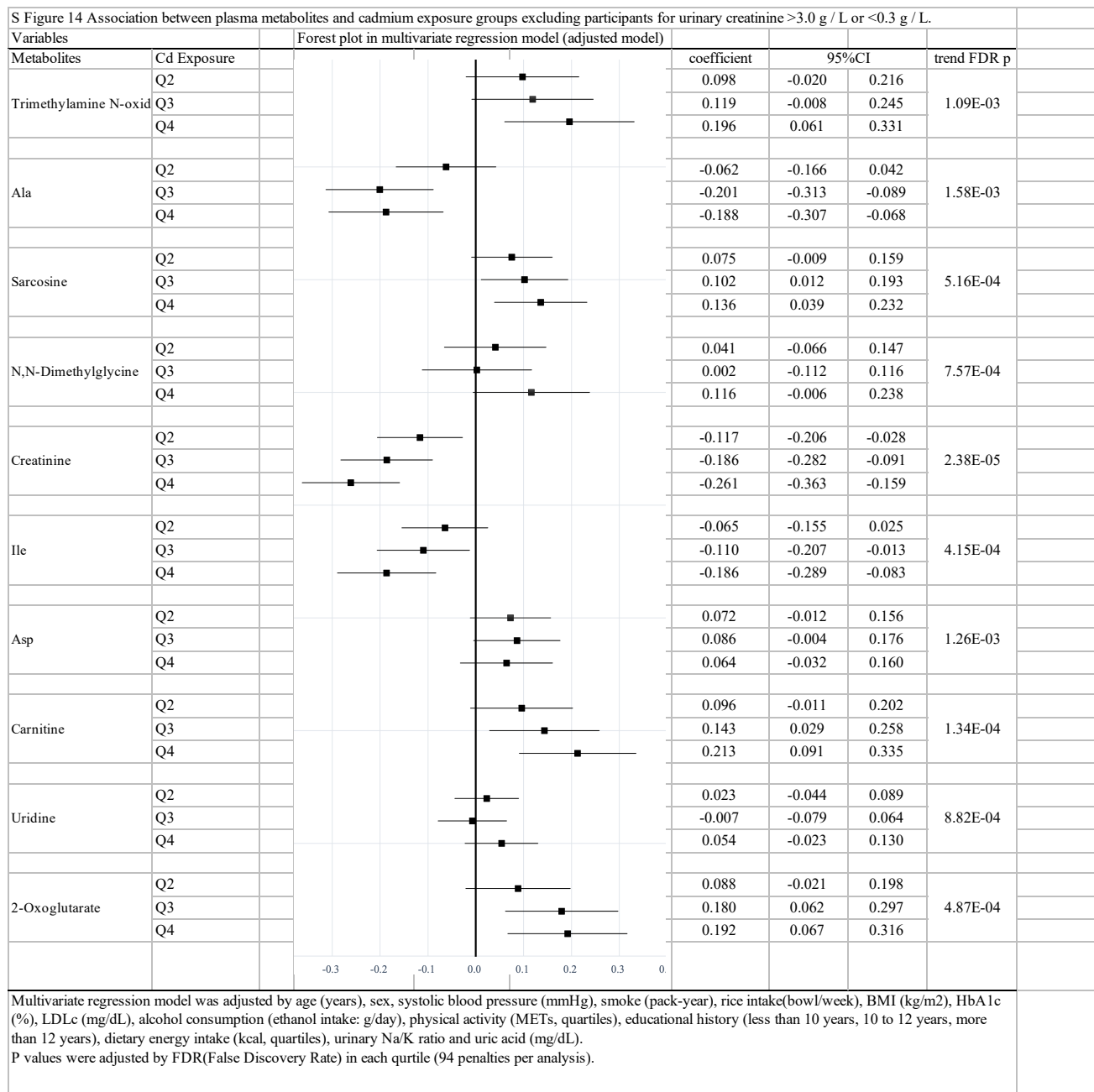

Supplement: Supplementary file 14 — Additional file 14: S Figure 14 Association between plasma metabolites and cadmium exposure groups excluding participants for urinary creatinine >3.0 g/L or <0.3 g/L. [file ehpm-29-022-s014.pdf]

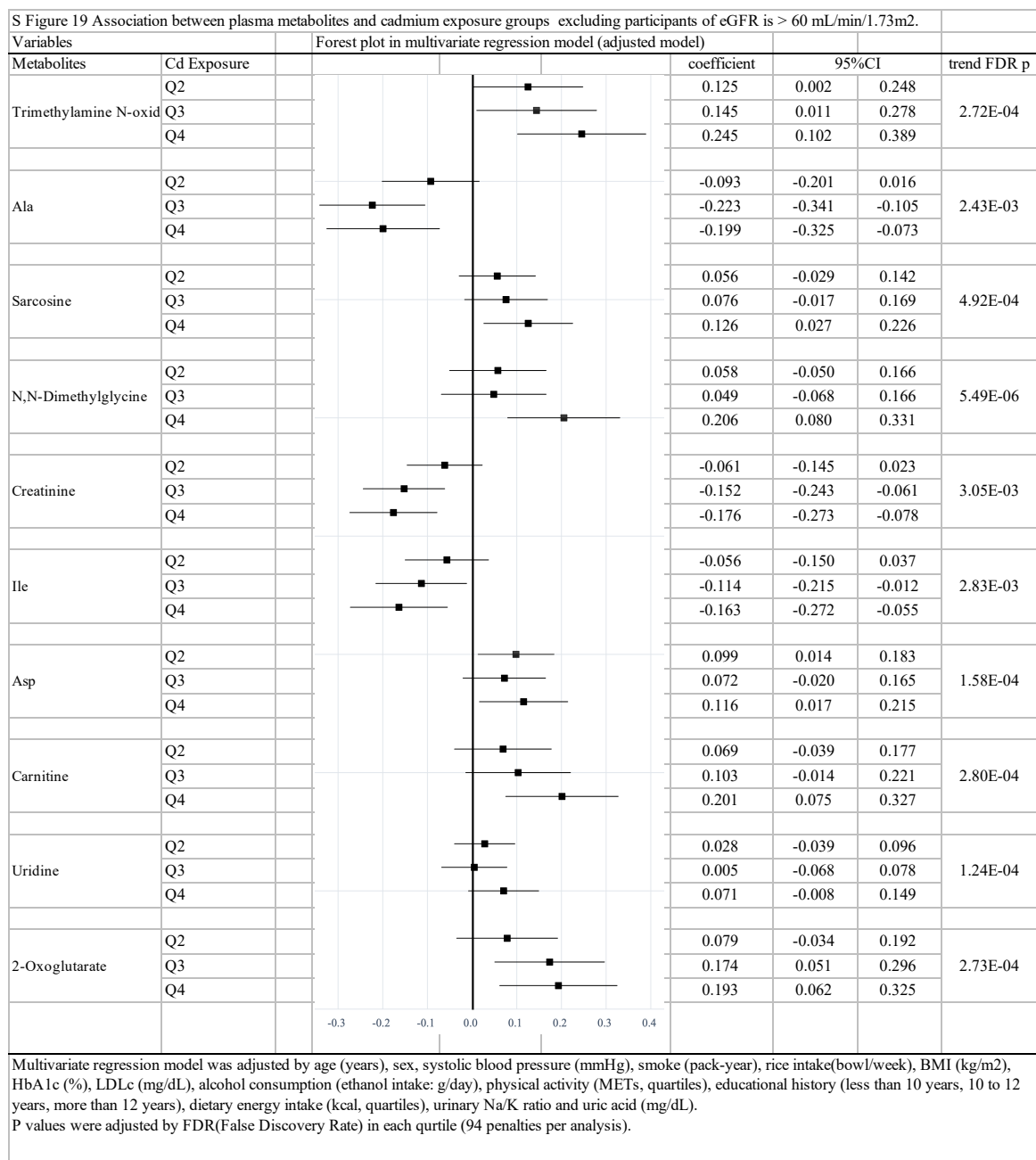

Supplement: Supplementary file 19 — Additional file 19: S Figure 19 Association between plasma metabolites and cadmium exposure groups excluding participants of eGFR is >60 mL/min/1.73 m2. [file ehpm-29-022-s019.pdf]

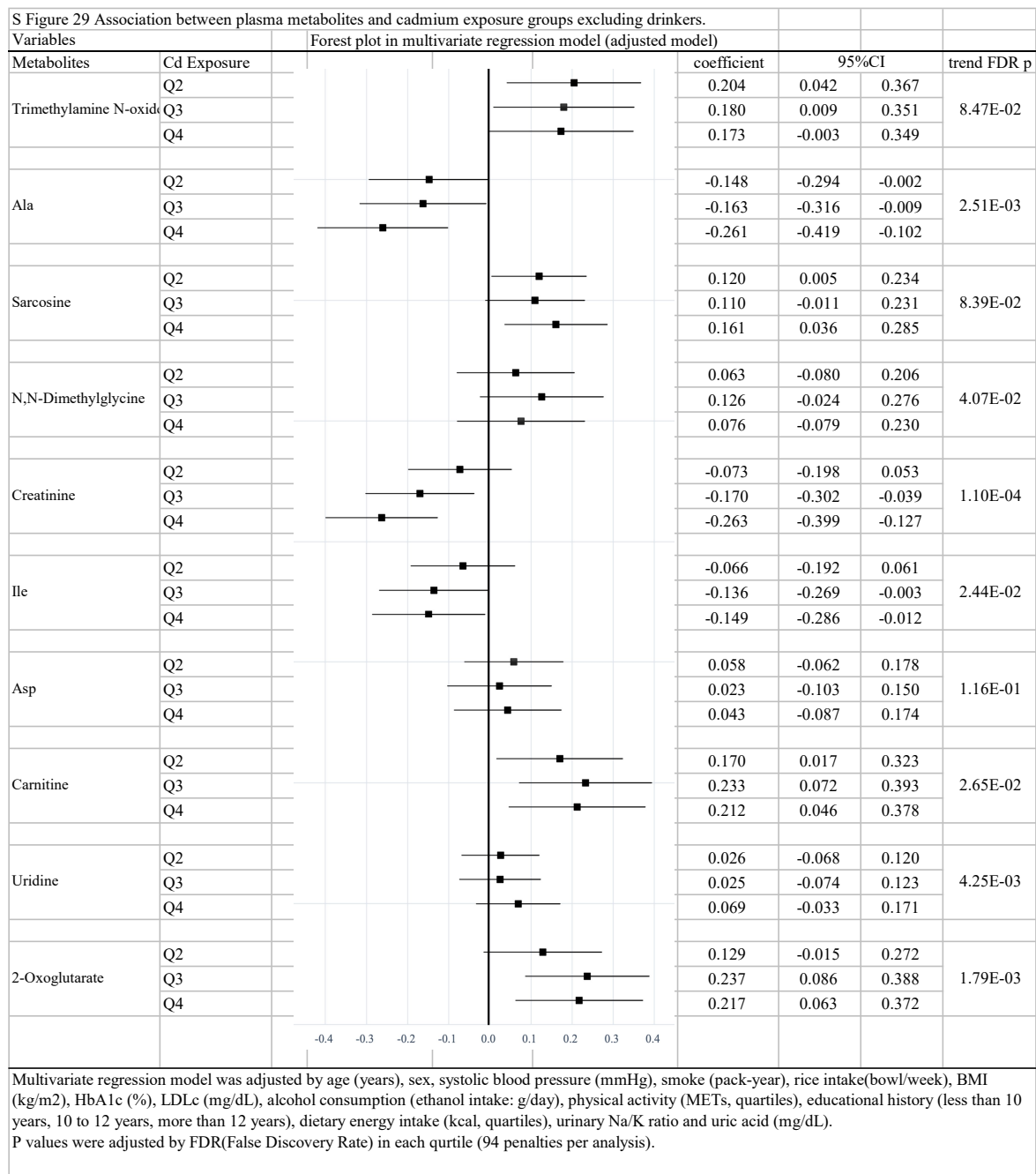

Supplement: Supplementary file 29 — Additional file 29: S Figure 29 Association between plasma metabolites and cadmium exposure groups excluding drinkers. [file ehpm-29-022-s029.pdf]
