# Supplementary material for: A population-based urinary and plasma metabolomics study of environmental exposure to cadmium
Source: Environ Health Prev Med. 2024 Mar 30;29:22. doi: 10.1265/ehpm.23-00218 (PMC10992994; doi:10.1265/ehpm.23-00218)
Supplement: Supplementary file 2 — Additional file 2: S Figure 2 Association between urine metabolites and cadmium exposure groups in multivariate regression model in men (Standalized β, Q2–Q4 compared to Q1). [file ehpm-29-022-s002.pdf]

S Figure 2 Association between urine metabolites and cadmium exposure groups in multivariate regression model in men (Standalized β, Q2-Q4 compared to Q1).

Variables	Trend FDR p	Variables	Trend FDR p
Trimethylamine N-oxide	1.53E-02	Succinate	5.01E-01
Ala	4.25E-02	Isethionate	2.42E-03
Choline	2.30E-02	5-Oxoproline	1.58E-02
Taurine	3.51E-01	Glutarate	1.05E-01
1-Methylnicotinamide	1.45E-02	Malate	7.28E-01
ADMA	2.16E-03	Threonate	1.24E-01
Uridine	1.46E-02	Ethanolamine phosphate	1.79E-05
Adenosine	2.34E-03	2-Oxoglutarate	4.89E-01
Ethanolamine	2.34E-03	Pimelate	3.69E-03
Piperidine	1.46E-02	Urate	2.11E-04
5-Aminolevulinate	1.38E-02	Glycerophosphate	1.30E-07
Allantoin	1.88E-01	trans-Aconitate	6.47E-02
7-Methylguanine	1.45E-02	cis-Aconitate	8.52E-02
N1-Acetylspermidine	1.91E-01	N-Acetylaspartate	7.99E-02
N8-Acetylspermidine	2.34E-01	Azelate	6.68E-02
Gly-Leu	7.16E-02	Isocitrate	1.77E-02
N-epsilon-Acetyllysine	3.74E-02	Adipate	6.56E-02
SAM+	6.29E-02	3-Hydroxy-3-methylglutarate	5.44E-01
1-Methyladenosine	1.26E-03	2,3-Pyridinedicarboxylate	8.45E-01
Lactate	4.29E-02	Suberate	4.64E-03
Malonate	9.46E-01	Gluconate	1.12E-01
4-Oxopentanoate	8.10E-02	Saccharate	5.37E-02
		N-Acetylneuraminate	5.37E-02

Forest plot (Standalized β, 95%CI)

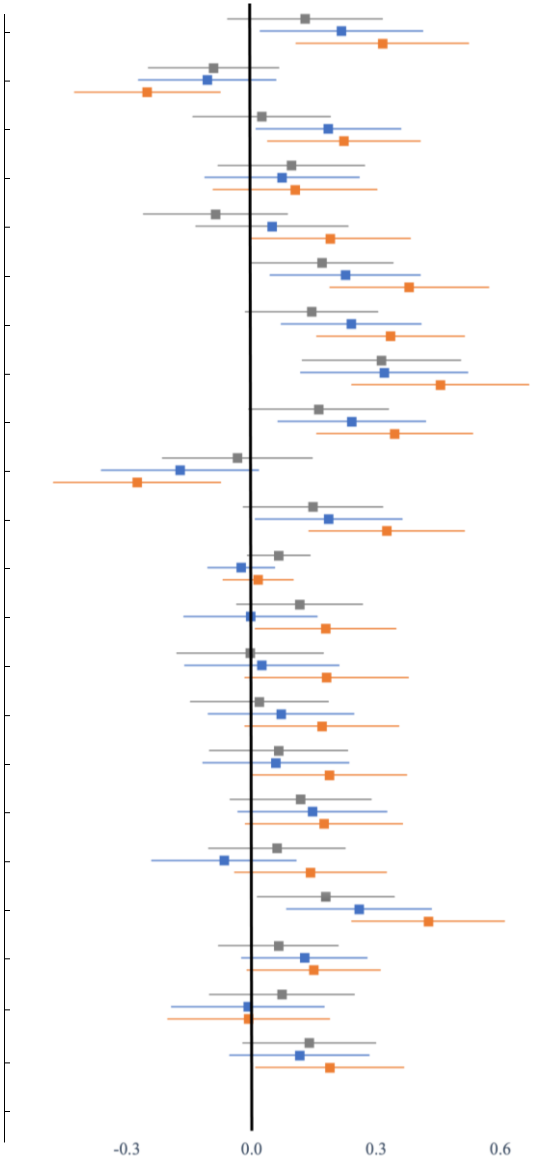

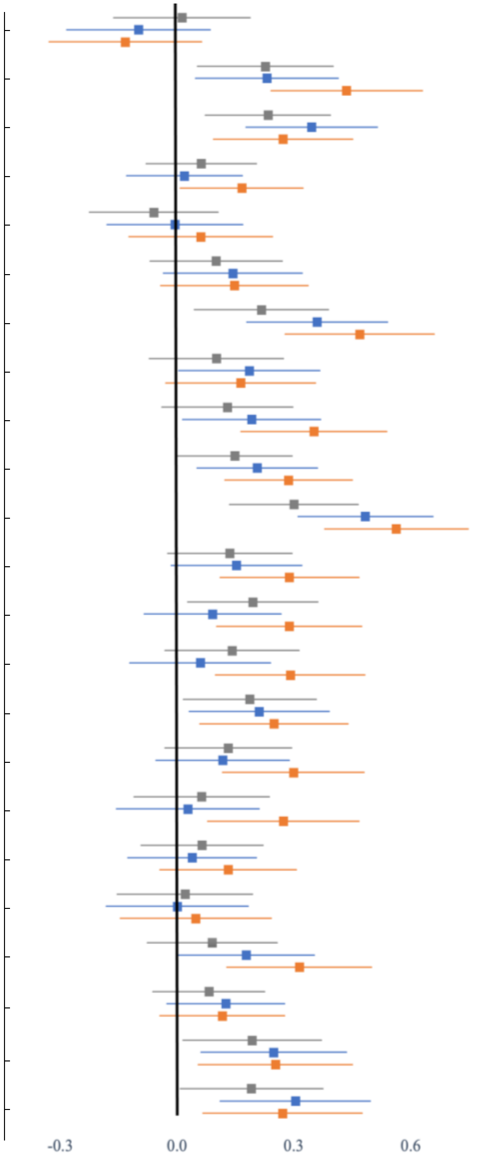

Multivariate regression model was adjusted by age (years),sex ,systolic blood pressure (mmHg), smoke (pack-year), rice intake(bowl/week), BMI (kg/m2), HbA1c (%), LDLc (mg/dL), alcohol consumption (ethanol intake: g/day), physical activity (METs, quartiles), educational history (less than 10 years, 10 to 12 years, more than 12 years), dietary energy intake (kcal, quartiles), urinary Na/K ratio and uric acid (mg/dL). Trend p values were adjusted by FDR(False Discovery Rate) in each qurtile (123 penalties per analysis).

S Figure 2 Association between urine metabolites and cadmium exposure groups in multvariate regression model in women (Standalized β, Q2-Q4 compared to Q1).

Variables	Forest plot (Standalized β, 95%CI)	Trend FDR p
Trimethylamine N-oxide		6.35E-01
Ala		4.95E-01
Choline		8.31E-01
Taurine		7.59E-02
1-Methylnicotinamide		7.79E-01
ADMA		7.59E-02
Uridine		4.53E-02
Adenosine		2.95E-01
Ethanolamine		7.79E-01
Piperidine		5.92E-01
5-Aminolevulinate		9.11E-03
Allantoin		3.79E-01
7-Methylguanine		2.48E-01
N1-Acetylspermidine		7.35E-01
N8-Acetylspermidine		2.84E-03
Gly-Leu		2.95E-01
N-epsilon-Acetyllysine		1.18E-01
SAM+		8.42E-03
1-Methyladenosine		4.53E-02
Lactate		2.38E-01
Malonate		2.28E-03
4-Oxopentanoate		8.65E-01

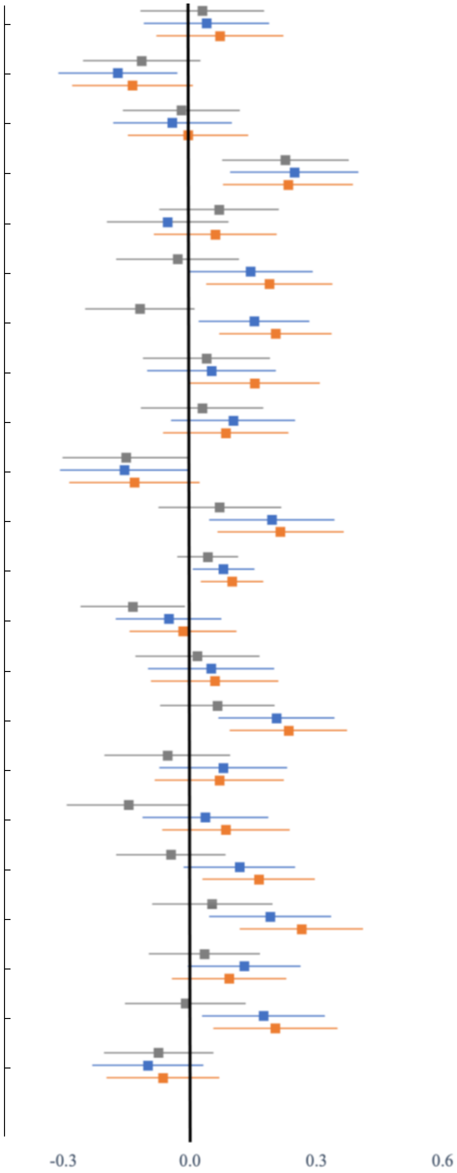

Variables	Forest plot (Standalized β, 95%CI)	Trend FDR p
Succinate		3.73E-03
Isethionate		3.49E-03
5-Oxoproline		1.06E-01
Glutarate		2.48E-01
Malate		2.53E-03
Threonate		2.16E-02
Ethanolamine phosphate		8.91E-02
2-Oxoglutarate		2.53E-03
Pimelate		1.03E-03
Urate		7.26E-03
Glycerophosphate		7.65E-01
trans-Aconitate		2.10E-01
cis-Aconitate		2.28E-03
N-Acetylaspartate		9.17E-06
Azelate		1.58E-02
Isocitrate		2.67E-05
Adipate		2.37E-03
3-Hydroxy-3-methylglutarate		2.53E-03
2,3-Pyridinedicarboxylate		1.58E-02
Suberate		2.53E-03
Gluconate		2.10E-01
Saccharate		1.23E-01
N-Acetylneuraminate		9.33E-02

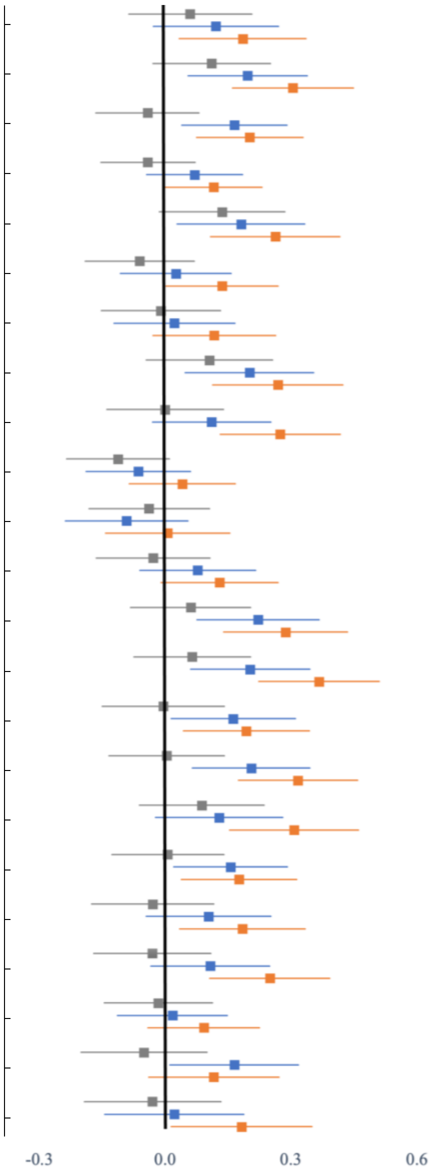

Multivariate regression model was adjusted by age (years),sex ,systolic blood pressure (mmHg), smoke (pack-year), rice intake(bowl/week), BMI (kg/m2), HbA1c (%), LDLc (mg/dL), alcohol consumption (ethanol intake: g/day), physical activity (METs, quartiles), educational history (less than 10 years, 10 to 12 years, more than 12 years), dietary energy intake (kcal, quartiles), urinary Na/K ratio and uric acid (mg/dL). Trend p values were adjusted by FDR(False Discovery Rate) in each qurtile (123 penalties per analysis).
