# Supplementary material for: A population-based urinary and plasma metabolomics study of environmental exposure to cadmium
Source: Environ Health Prev Med. 2024 Mar 30;29:22. doi: 10.1265/ehpm.23-00218 (PMC10992994; doi:10.1265/ehpm.23-00218)
Supplement: Supplementary file 3 — Additional file 3: S Figure 3 Association between urine metabolites and cadmium exposure groups in univariate regression model in total population (Standalized β, Q2–Q4 compared to Q1). [file ehpm-29-022-s003.pdf]

S Figure 3 Association between urine metabolites and cadmium exposure groups in univariate regression model in total population (Standalized β, Q2-Q4 compared to Q1).

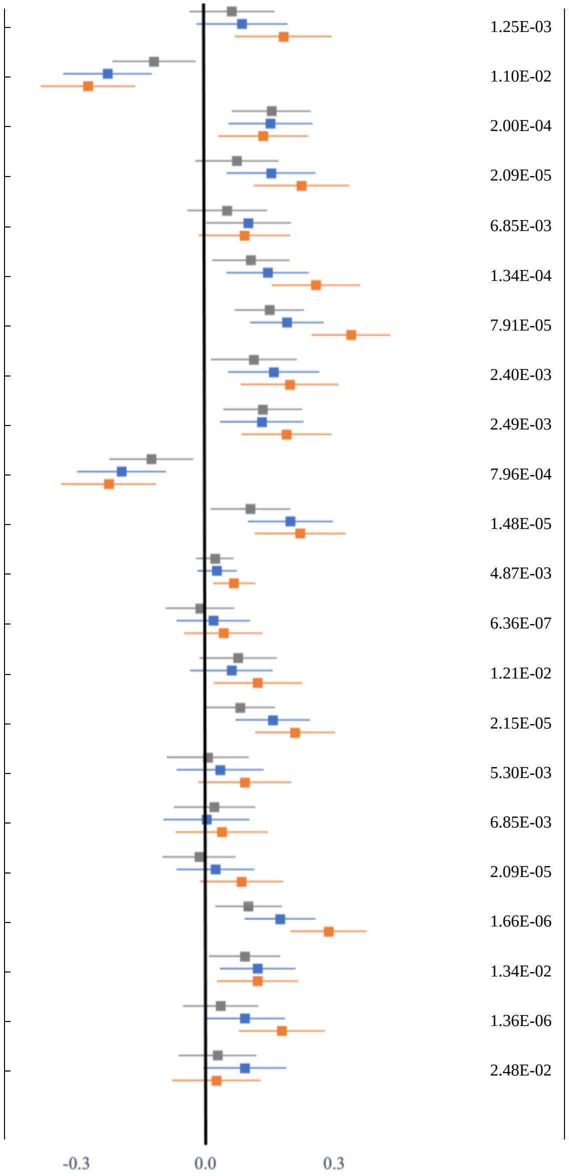


| Variables | Trend FDR p |
|---|---|
| Trimethylamine N-oxide | 1.25E-03 |
| Ala | 1.10E-02 |
| Choline | 2.00E-04 |
| Taurine | 2.09E-05 |
| 1-Methylnicotinamide | 6.85E-03 |
| ADMA | 1.34E-04 |
| Uridine | 7.91E-05 |
| Adenosine | 2.40E-03 |
| Ethanolamine | 2.49E-03 |
| Piperidine | 7.96E-04 |
| 5-Aminolevulinate | 1.48E-05 |
| Allantoin | 4.87E-03 |
| 7-Methylguanine | 6.36E-07 |
| N1-Acetylspermidine | 1.21E-02 |
| N8-Acetylspermidine | 2.15E-05 |
| Gly-Leu | 5.30E-03 |
| N-epsilon-Acetyllysine | 6.85E-03 |
| SAM+ | 2.09E-05 |
| 1-Methyladenosine | 1.66E-06 |
| Lactate | 1.34E-02 |
| Malonate | 1.36E-06 |
| 4-Oxopentanoate | 2.48E-02 |

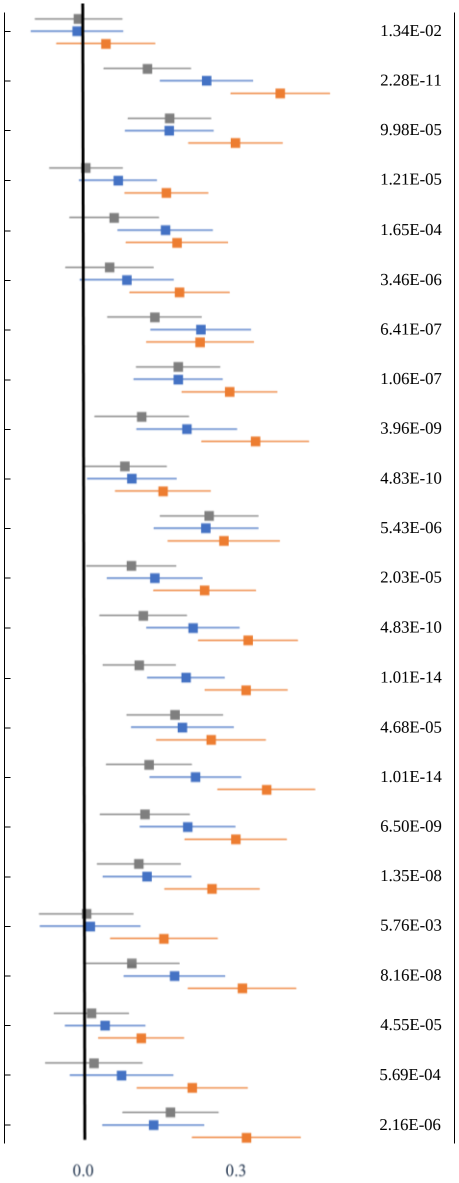


| Variables | Trend FDR p |
|---|---|
| Succinate | 1.34E-02 |
| Isethionate | 2.28E-11 |
| 5-Oxoproline | 9.98E-05 |
| Glutarate | 1.21E-05 |
| Malate | 1.65E-04 |
| Threonate | 3.46E-06 |
| Ethanolamine phosphate | 6.41E-07 |
| 2-Oxoglutarate | 1.06E-07 |
| Pimelate | 3.96E-09 |
| Urate | 4.83E-10 |
| Glycerophosphate | 5.43E-06 |
| trans-Aconitate | 2.03E-05 |
| cis-Aconitate | 4.83E-10 |
| N-Acetylaspartate | 1.01E-14 |
| Azelate | 4.68E-05 |
| Isocitrate | 1.01E-14 |
| Adipate | 6.50E-09 |
| 3-Hydroxy-3-methylglutarate | 1.35E-08 |
| 2,3-Pyridinedicarboxylate | 5.76E-03 |
| Suberate | 8.16E-08 |
| Gluconate | 4.55E-05 |
| Saccharate | 5.69E-04 |
| N-Acetylneuraminate | 2.16E-06 |

Multivariate regression model was adjusted by age (years),sex ,systolic blood pressure (mmHg), smoke (pack-year), rice intake(bowl/week), BMI (kg/m2), HbA1c (%), LDLc (mg/dL), alcohol consumption (ethanol intake: g/day), physical activity (METs, quartiles), educational history (less than 10 years, 10 to 12 years, more than 12 years), dietary energy intake (kcal, quartiles), urinary Na/K ratio and uric acid (mg/dL). Trend p values were adjusted by FDR(False Discovery Rate) in each qurtile (123 penalties per analysis).
