# Supplementary material for: A population-based urinary and plasma metabolomics study of environmental exposure to cadmium
Source: Environ Health Prev Med. 2024 Mar 30;29:22. doi: 10.1265/ehpm.23-00218 (PMC10992994; doi:10.1265/ehpm.23-00218)
Supplement: Supplementary file 4 — Additional file 4: S Figure 4 Association between plasma metabolites and cadmium exposure groups in multivariate regression model in sex stratification (Standalized β, Q2–Q4 compared to Q1). [file ehpm-29-022-s004.pdf]

S Figure 4 Association between plasma metabolites and cadmium exposure groups in multivariate regression model in sex stratification (Standalized β, Q2-Q4 compared to Q1).

Variables (Men)	Forest plot (Standalized β, 95%CI).	Trend FDR p	Variables (Women)	Forest plot (Standalized β, 95%CI).	Trend FDR p
Trimethylamine N-oxide		1.42E-02	Trimethylamine N-oxide		2.22E-01
Ala		1.47E-01	Ala		4.05E-02
Sarcosine		3.57E-01	Sarcosine		5.79E-02
N,N-Dimethylglycine		4.79E-01	N,N-Dimethylglycine		5.79E-02
Creatinine		1.06E-02	Creatinine		9.29E-03
Ile		5.12E-01	Ile		5.79E-02
Asp		3.57E-01	Asp		1.48E-02
Carnitine		4.79E-01	Carnitine		1.01E-02
Uridine		6.56E-01	Uridine		9.29E-03
2-Oxoglutarate		2.61E-01	2-Oxoglutarate		6.60E-01

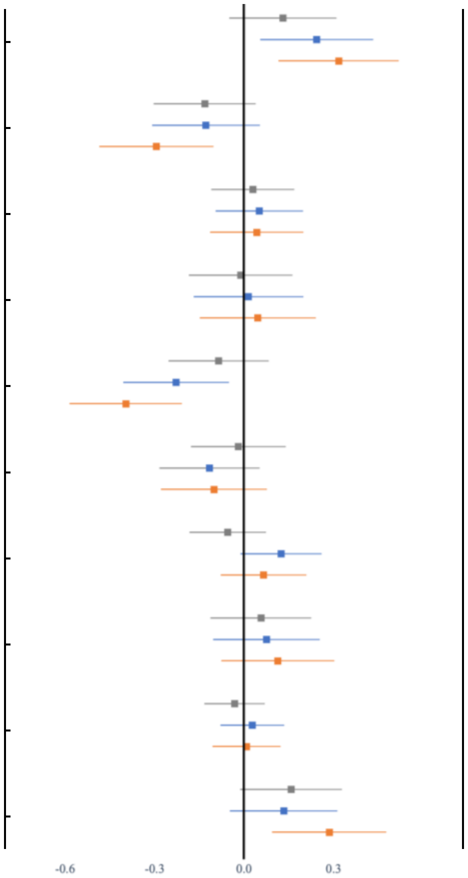

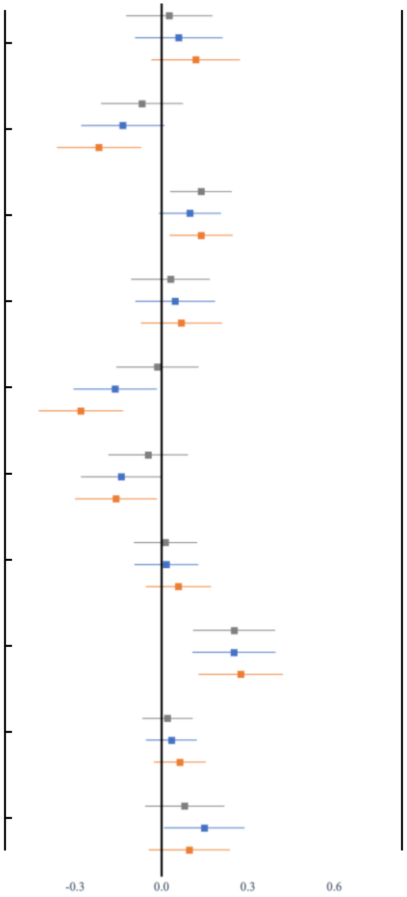

Multivariate regression model was adjusted by age (years), sex, systolic blood pressure (mmHg), smoke (brinkman index), rice intake(bowl/week), BMI (kg/m2), HbA1c (%), LDLc (mg/dL), alcohol consumption (ethanol intake: g/day), physical activity (METs, quartiles), educational history (less than 10 years, 10 to 12 years, more than 12 years), dietary energy intake (kcal, quartiles), urinary Na/K ratio and uric acid (mg/dL).
P values were adjusted by FDR(False Discovery Rate) in each qurtile (94 penalties per analysis).
