# Supplementary material for: A population-based urinary and plasma metabolomics study of environmental exposure to cadmium
Source: Environ Health Prev Med. 2024 Mar 30;29:22. doi: 10.1265/ehpm.23-00218 (PMC10992994; doi:10.1265/ehpm.23-00218)
Supplement: Supplementary file 5 — Additional file 5: S Figure 5 Association between plasma metabolites and cadmium exposure groups in univariate regression model (Standalized β, Q2–Q4 compared to Q1). [file ehpm-29-022-s005.pdf]

S Figure 5 Association between plasma metabolites and cadmium exposure groups in univariate regression model (Standalized β, Q2-Q4 compared to Q1).

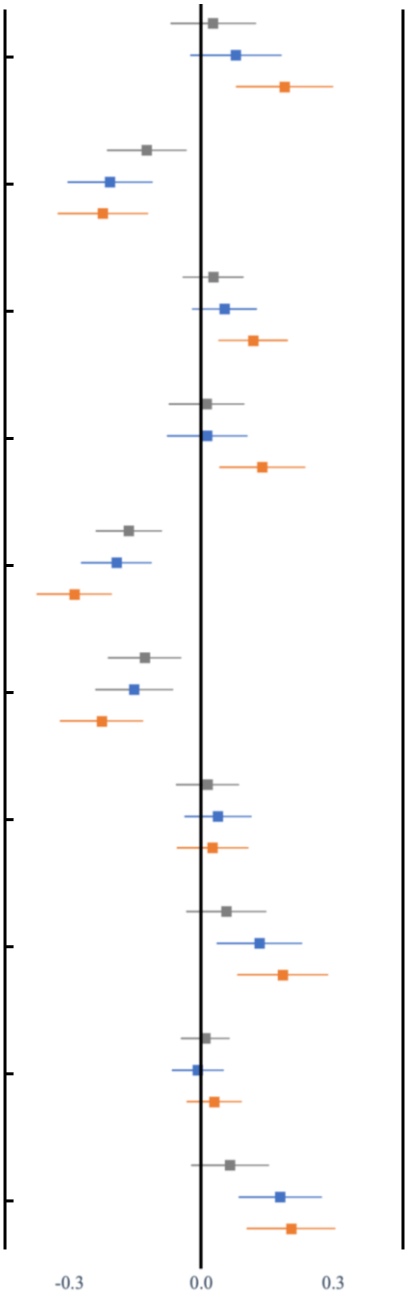

Variables (Men)	Forest plot (Standalized β, 95%CI	Trend FDR p
Trimethylamine N-oxide		1.21E-03
Ala		2.29E-04
Sarcosine		5.00E-03
N,N-Dimethylglycine		7.17E-05
Creatinine		1.52E-06
Ile		1.45E-04
Asp		2.18E-02
Carnitine		2.29E-04
Uridine		4.31E-03
2-Oxoglutarate		8.10E-05

Multivariate regression model was adjusted by age (years), sex, systolic blood pressure (mmHg), smoke (brinkman index), rice intake(bowl/week), BMI (kg/m2), HbA1c (%), LDLc (mg/dL), alcohol consumption (ethanol intake: g/day), physical activity (METs, quartiles), educational history (less than 10 years, 10 to 12 years, more than 12 years), dietary energy intake (kcal, quartiles), urinary Na/K ratio and uric acid (mg/dL).
P values were adjusted by FDR(False Discovery Rate) in each qurtile (94 penalties per analysis).
