# Supplementary material for: A population-based urinary and plasma metabolomics study of environmental exposure to cadmium
Source: Environ Health Prev Med. 2024 Mar 30;29:22. doi: 10.1265/ehpm.23-00218 (PMC10992994; doi:10.1265/ehpm.23-00218)
Supplement: Supplementary file 6 — Additional file 6: S Figure 6 Association between urine metabolites and cadmium exposure groups in nonsmoker. [file ehpm-29-022-s006.pdf]

S Figure 6 Association between urine metabolites and cadmium exposure groups in nonsmoker

Forest plot in multivariate regression model (adjusted model)

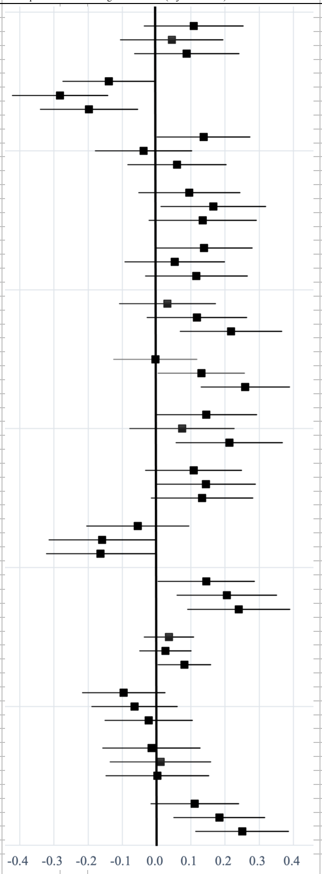

Variables: Metabolites	Cd Exposure	Standardized β	95%CI		trend FDR p
	Q2	0.108	-0.036	0.253	
Trimethylamine N-oxide	Q3	0.044	-0.105	0.194	3.32E-01
	Q4	0.088	-0.065	0.241	
	Q2	-0.139	-0.274	-0.005	
Ala	Q3	-0.282	-0.422	-0.143	1.93E-02
	Q4	-0.198	-0.340	-0.055	
	Q2	0.137	0.002	0.273	
Choline	Q3	-0.038	-0.179	0.102	3.79E-01
	Q4	0.059	-0.084	0.203	
	Q2	0.095	-0.053	0.243	
Taurine	Q3	0.166	0.012	0.319	6.37E-02
	Q4	0.135	-0.022	0.291	
	Q2	0.139	-0.002	0.279	
1-Methylnicotinamide	Q3	0.053	-0.092	0.199	1.17E-01
	Q4	0.116	-0.033	0.265	
	Q2	0.032	-0.108	0.172	
ADMA	Q3	0.118	-0.028	0.263	5.73E-03
	Q4	0.217	0.069	0.366	
	Q2	-0.004	-0.126	0.118	
Uridine	Q3	0.131	0.004	0.257	8.44E-04
	Q4	0.259	0.130	0.388	
	Q2	0.145	-0.002	0.292	
Adenosine	Q3	0.074	-0.078	0.227	2.53E-02
	Q4	0.212	0.057	0.368	
	Q2	0.108	-0.032	0.248	
Ethanolamine	Q3	0.144	-0.002	0.289	1.96E-01
	Q4	0.133	-0.016	0.282	
	Q2	-0.055	-0.205	0.094	
Piperidine	Q3	-0.159	-0.314	-0.004	4.13E-02
	Q4	-0.164	-0.322	-0.005	
	Q2	0.145	0.004	0.286	
5-Aminolevulinate	Q3	0.205	0.059	0.351	5.03E-04
	Q4	0.240	0.091	0.390	
	Q2	0.036	-0.036	0.109	
Allantoin	Q3	0.026	-0.049	0.101	3.10E-02
	Q4	0.081	0.005	0.158	
	Q2	-0.096	-0.217	0.025	
7-Methylguanine	Q3	-0.064	-0.189	0.061	1.00E-02
	Q4	-0.023	-0.151	0.105	
	Q2	-0.015	-0.157	0.127	
N1-Acetylspermidine	Q3	0.011	-0.136	0.159	7.09E-01
	Q4	0.002	-0.148	0.153	
	Q2	0.111	-0.017	0.240	
N8-Acetylspermidine	Q3	0.183	0.050	0.316	2.69E-04
	Q4	0.250	0.114	0.386	

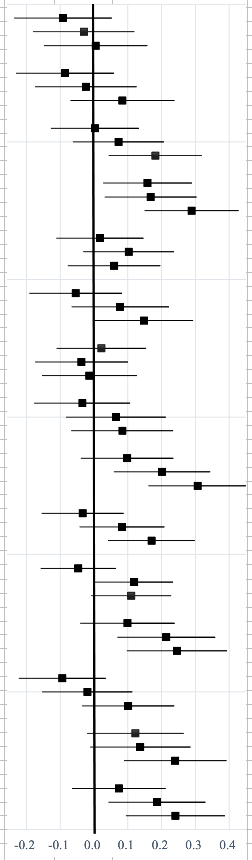

Variables: Metabolites	Cd Exposure	Standardized β	95%CI		trend FDR p
	Q2	-0.092	-0.235	0.052	
Gly-Leu	Q3	-0.030	-0.179	0.119	2.47E-01
	Q4	0.005	-0.147	0.157	
	Q2	-0.086	-0.230	0.058	
N-epsilon-Acetyllysine	Q3	-0.024	-0.174	0.126	2.88E-02
	Q4	0.084	-0.069	0.237	
	Q2	0.003	-0.126	0.132	
SAM+	Q3	0.073	-0.061	0.206	6.65E-05
	Q4	0.182	0.045	0.318	
	Q2	0.158	0.027	0.288	
1-Methyladenosine	Q3	0.167	0.032	0.303	1.05E-03
	Q4	0.289	0.150	0.427	
	Q2	0.018	-0.111	0.146	
Lactate	Q3	0.103	-0.031	0.236	1.40E-01
	Q4	0.059	-0.077	0.196	
	Q2	-0.055	-0.191	0.082	
Malonate	Q3	0.076	-0.066	0.218	1.18E-03
	Q4	0.148	0.003	0.292	
	Q2	0.022	-0.109	0.153	
4-Oxopentanoate	Q3	-0.037	-0.174	0.099	6.55E-01
	Q4	-0.013	-0.153	0.126	
	Q2	-0.035	-0.176	0.106	
Succinate	Q3	0.065	-0.082	0.212	1.35E-02
	Q4	0.084	-0.067	0.234	
	Q2	0.098	-0.038	0.234	
Isethionate	Q3	0.202	0.060	0.344	2.07E-04
	Q4	0.307	0.162	0.452	
	Q2	-0.033	-0.154	0.087	
5-Oxoproline	Q3	0.083	-0.042	0.208	1.45E-01
	Q4	0.170	0.042	0.298	
	Q2	-0.046	-0.157	0.064	
Glutarate	Q3	0.118	0.003	0.233	5.60E-02
	Q4	0.110	-0.007	0.228	
	Q2	0.099	-0.040	0.238	
Malate	Q3	0.214	0.070	0.358	1.58E-04
	Q4	0.245	0.098	0.393	
	Q2	-0.094	-0.221	0.034	
Threonate	Q3	-0.020	-0.153	0.113	1.62E-02
	Q4	0.101	-0.035	0.237	
	Q2	0.122	-0.020	0.264	
Ethanolamine phosphate	Q3	0.137	-0.011	0.285	5.85E-04
	Q4	0.240	0.090	0.391	
	Q2	0.074	-0.064	0.211	
2-Oxoglutarate	Q3	0.186	0.043	0.329	2.64E-04
	Q4	0.241	0.095	0.387	

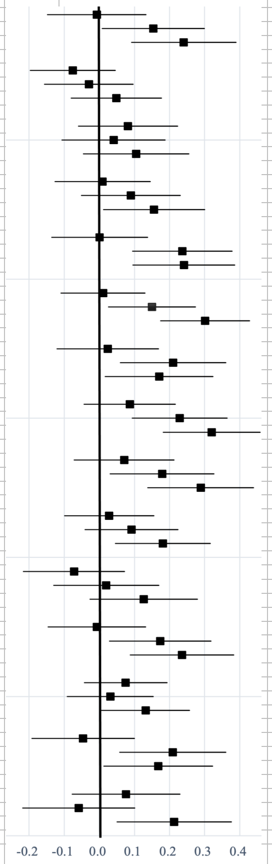

Variables: Metabolites	Cd Exposure	Standardized β	95%CI		trend FDR p
	Q2	-0.007	-0.148	0.133	
Pimelate	Q3	0.154	0.008	0.299	2.42E-04
	Q4	0.240	0.092	0.389	
	Q2	-0.076	-0.197	0.045	
Urate	Q3	-0.030	-0.156	0.096	7.20E-04
	Q4	0.049	-0.080	0.177	
	Q2	0.082	-0.060	0.223	
Glycerophosphate	Q3	0.041	-0.106	0.188	1.11E-01
	Q4	0.105	-0.045	0.255	
	Q2	0.009	-0.126	0.145	
trans-Aconitate	Q3	0.090	-0.052	0.231	4.34E-02
	Q4	0.156	0.011	0.300	
	Q2	0.001	-0.136	0.137	
cis-Aconitate	Q3	0.237	0.094	0.379	2.25E-04
	Q4	0.241	0.096	0.386	
	Q2	0.010	-0.109	0.130	
N-Acetylaspartate	Q3	0.150	0.026	0.274	1.86E-07
	Q4	0.301	0.174	0.428	
	Q2	0.024	-0.121	0.168	
Azelate	Q3	0.210	0.060	0.361	1.12E-02
	Q4	0.170	0.017	0.324	
	Q2	0.087	-0.043	0.216	
Isocitrate	Q3	0.229	0.094	0.364	7.59E-07
	Q4	0.320	0.182	0.458	
	Q2	0.071	-0.071	0.213	
Adipate	Q3	0.179	0.031	0.326	1.02E-04
	Q4	0.289	0.138	0.440	
	Q2	0.028	-0.100	0.155	
3-Hydroxy-3-methylglutarate	Q3	0.092	-0.040	0.225	6.11E-04
	Q4	0.181	0.046	0.316	
	Q2	-0.072	-0.216	0.072	
2,3-Pyridinedicarboxylate	Q3	0.020	-0.130	0.169	3.69E-02
	Q4	0.126	-0.027	0.280	
	Q2	-0.007	-0.146	0.132	
Suberate	Q3	0.173	0.029	0.318	4.72E-04
	Q4	0.235	0.088	0.383	
	Q2	0.075	-0.043	0.193	
Gluconate	Q3	0.031	-0.092	0.154	3.88E-02
	Q4	0.132	0.006	0.257	
	Q2	-0.046	-0.192	0.100	
Saccharate	Q3	0.209	0.057	0.361	5.27E-03
	Q4	0.168	0.013	0.323	
	Q2	0.076	-0.078	0.229	
N-Acetylneuraminate	Q3	-0.059	-0.219	0.101	5.52E-02
	Q4	0.213	0.050	0.376	

Multivariate regression model was adjusted by age (years),sex ,systolic blood pressure (mmHg), smoke (pack-year), rice intake(bowl/week), BMI (kg/m2), HbA1c (%), LDLc (mg/dL), alcohol consumption (ethanol intake: g/day), physical activity (METs, quartiles), educational history (less than 10 years, 10 to 12 years, more than 12 years), dietary energy intake (kcal, quartiles), urinary Na/K ratio and uric acid (mg/dL).
Trend p values were adjusted by FDR(False Discovery Rate) in each qurtile (123 penalties per analysis).
