# Supplementary material for: A population-based urinary and plasma metabolomics study of environmental exposure to cadmium
Source: Environ Health Prev Med. 2024 Mar 30;29:22. doi: 10.1265/ehpm.23-00218 (PMC10992994; doi:10.1265/ehpm.23-00218)
Supplement: Supplementary file 7 — Additional file 7: S Figure 7 Association between urine metabolites and cadmium exposure groups in multvariate regression model in nonsmoker men. [file ehpm-29-022-s007.pdf]

S Figure 7 Association between urine metabolites and cadmium exposure groups in multivariate regression model in nonsmoker men.

Forest plot in multivariate regression model (adjusted model)

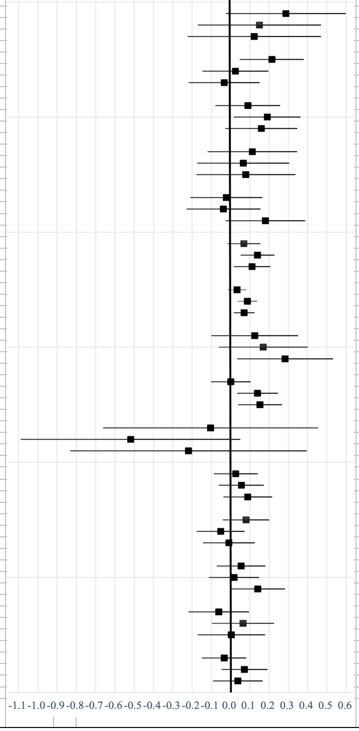

Variables: Metabolites	Cd Exposure	Standardized β	95%CI		trend FDR p
Trimethylamine N-oxide	Q2	0.287	-0.023	0.597	5.85E-01
	Q3	0.149	-0.170	0.468	
	Q4	0.123	-0.222	0.468	
Ala	Q2	0.214	0.049	0.379	1.77E-01
	Q3	0.025	-0.144	0.195	
	Q4	-0.033	-0.216	0.150	
Choline	Q2	0.090	-0.077	0.256	5.79E-02
	Q3	0.190	0.018	0.362	
	Q4	0.160	-0.025	0.346	
Taurine	Q2	0.112	-0.118	0.343	6.19E-01
	Q3	0.065	-0.172	0.302	
	Q4	0.079	-0.177	0.335	
1-Methylnicotinamide	Q2	-0.021	-0.207	0.164	6.39E-02
	Q3	-0.037	-0.227	0.154	
	Q4	0.181	-0.025	0.386	
ADMA	Q2	0.070	-0.015	0.154	9.75E-03
	Q3	0.140	0.054	0.227	
	Q4	0.111	0.018	0.204	
Uridine	Q2	0.034	-0.013	0.081	1.52E-02
	Q3	0.088	0.039	0.136	
	Q4	0.070	0.018	0.123	
Adenosine	Q2	0.126	-0.098	0.350	1.62E-02
	Q3	0.169	-0.061	0.398	
	Q4	0.283	0.035	0.531	
Ethanolamine	Q2	0.002	-0.100	0.103	4.56E-04
	Q3	0.140	0.035	0.244	
	Q4	0.153	0.040	0.265	
Piperidine	Q2	-0.104	-0.660	0.452	2.97E-01
	Q3	-0.519	-1.087	0.049	
	Q4	-0.219	-0.831	0.394	
5-Aminolevulinate	Q2	0.026	-0.086	0.139	2.07E-01
	Q3	0.056	-0.060	0.171	
	Q4	0.089	-0.036	0.214	
Allantoin	Q2	0.080	-0.039	0.199	4.13E-01
	Q3	-0.052	-0.175	0.072	
	Q4	-0.009	-0.142	0.124	
7-Methylguanine	Q2	0.055	-0.069	0.179	1.05E-02
	Q3	0.018	-0.111	0.146	
	Q4	0.142	0.003	0.281	
N1-Acetylspermidine	Q2	-0.061	-0.216	0.094	5.57E-01
	Q3	0.065	-0.096	0.225	
	Q4	0.004	-0.169	0.177	
N8-Acetylspermidine	Q2	-0.033	-0.148	0.081	2.45E-01
	Q3	0.072	-0.046	0.190	
	Q4	0.038	-0.090	0.166	

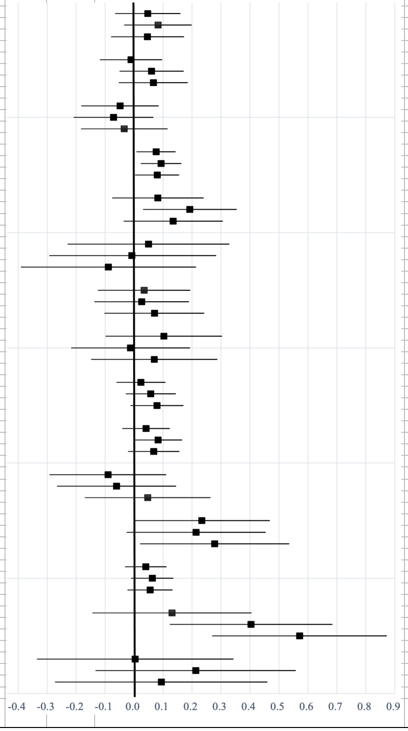

Variables: Metabolites	Cd Exposure	Standardized β	95%CI		trend FDR p
Gly-Leu	Q2	0.047	-0.065	0.159	1.82E-01
	Q3	0.082	-0.033	0.198	
	Q4	0.046	-0.079	0.171	
N-epsilon-Acetyllysine	Q2	-0.010	-0.117	0.096	6.71E-02
	Q3	0.061	-0.049	0.170	
	Q4	0.066	-0.052	0.185	
SAM+	Q2	-0.049	-0.182	0.084	5.56E-01
	Q3	-0.071	-0.208	0.066	
	Q4	-0.034	-0.182	0.114	
1-Methyladenosine	Q2	0.076	0.009	0.142	1.82E-02
	Q3	0.093	0.024	0.162	
	Q4	0.079	0.005	0.154	
Lactate	Q2	0.082	-0.075	0.240	7.37E-02
	Q3	0.193	0.032	0.353	
	Q4	0.135	-0.035	0.305	
Malonate	Q2	0.049	-0.229	0.328	2.89E-01
	Q3	-0.008	-0.292	0.276	
	Q4	-0.089	-0.390	0.212	
4-Oxopentanoate	Q2	0.034	-0.124	0.193	4.35E-01
	Q3	0.026	-0.136	0.188	
	Q4	0.070	-0.102	0.242	
Succinate	Q2	0.102	-0.098	0.302	8.15E-01
	Q3	-0.012	-0.217	0.192	
	Q4	0.069	-0.147	0.286	
Isethionate	Q2	0.024	-0.060	0.107	9.56E-02
	Q3	0.058	-0.028	0.143	
	Q4	0.079	-0.012	0.170	
5-Oxoproline	Q2	0.041	-0.040	0.123	2.78E-01
	Q3	0.082	-0.001	0.165	
	Q4	0.067	-0.020	0.155	
Glutarate	Q2	-0.090	-0.291	0.110	9.98E-01
	Q3	-0.061	-0.265	0.144	
	Q4	0.047	-0.169	0.263	
Malate	Q2	0.234	0.000	0.467	9.71E-02
	Q3	0.214	-0.025	0.453	
	Q4	0.278	0.021	0.535	
Threonate	Q2	0.040	-0.031	0.111	4.72E-01
	Q3	0.062	-0.010	0.135	
	Q4	0.055	-0.022	0.132	
Ethanolamine phosphate	Q2	0.131	-0.143	0.404	5.25E-05
	Q3	0.404	0.124	0.683	
	Q4	0.571	0.271	0.872	
2-Oxoglutarate	Q2	0.004	-0.334	0.342	4.41E-01
	Q3	0.213	-0.133	0.558	
	Q4	0.094	-0.272	0.459	

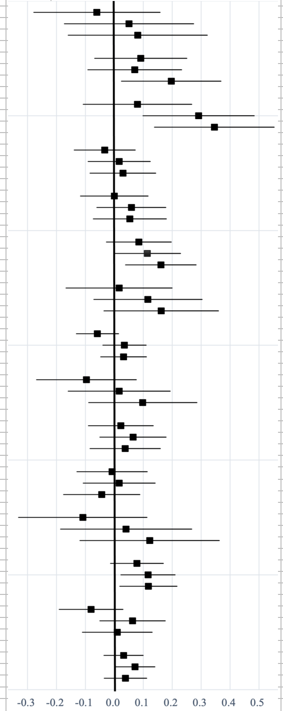

Variables: Metabolites	Cd Exposure	Standardized β	95%CI		trend FDR p
Pimelate	Q2	-0.060	-0.279	0.158	2.78E-01
	Q3	0.051	-0.173	0.274	
	Q4	0.081	-0.160	0.322	
Urate	Q2	0.091	-0.068	0.251	4.78E-02
	Q3	0.071	-0.092	0.233	
	Q4	0.197	0.025	0.369	
Glycerophosphate	Q2	0.080	-0.108	0.268	1.96E-04
	Q3	0.292	0.100	0.484	
	Q4	0.346	0.139	0.553	
trans-Aconitate	Q2	-0.033	-0.139	0.073	6.68E-01
	Q3	0.017	-0.091	0.125	
	Q4	0.030	-0.084	0.144	
cis-Aconitate	Q2	0.000	-0.117	0.117	6.33E-01
	Q3	0.059	-0.060	0.179	
	Q4	0.054	-0.073	0.180	
N-Acetylaspartate	Q2	0.085	-0.028	0.198	1.00E-01
	Q3	0.114	-0.001	0.229	
	Q4	0.161	0.039	0.283	
Azelate	Q2	0.017	-0.167	0.200	1.03E-01
	Q3	0.116	-0.071	0.304	
	Q4	0.162	-0.036	0.361	
Isocitrate	Q2	-0.058	-0.132	0.015	2.13E-01
	Q3	0.035	-0.039	0.110	
	Q4	0.032	-0.047	0.111	
Adipate	Q2	-0.097	-0.269	0.076	1.63E-01
	Q3	0.017	-0.160	0.193	
	Q4	0.098	-0.089	0.286	
3-Hydroxy-3-methylglutarate	Q2	0.023	-0.090	0.135	8.76E-01
	Q3	0.065	-0.050	0.180	
	Q4	0.038	-0.084	0.159	
2,3-Pyridinedicarboxylate	Q2	-0.007	-0.129	0.115	3.89E-01
	Q3	0.017	-0.107	0.142	
	Q4	-0.043	-0.175	0.089	
Suberate	Q2	-0.109	-0.331	0.114	1.29E-01
	Q3	0.041	-0.186	0.268	
	Q4	0.122	-0.119	0.364	
Gluconate	Q2	0.078	-0.014	0.170	2.24E-01
	Q3	0.117	0.023	0.210	
	Q4	0.118	0.019	0.217	
Saccharate	Q2	-0.080	-0.191	0.030	3.05E-01
	Q3	0.063	-0.050	0.176	
	Q4	0.011	-0.110	0.131	
N-Acetylneuraminate	Q2	0.032	-0.036	0.100	3.15E-01
	Q3	0.072	0.002	0.141	
	Q4	0.039	-0.035	0.113	

Multivariate regression model was adjusted by age (years),sex ,systolic blood pressure (mmHg), smoke (pack-year), rice intake(bowl/week), BMI (kg/m2), HbA1c (%), LDLc (mg/dL), alcohol consumption (ethanol intake: g/day), physical activity (METs, quartiles), educational history (less than 10 years, 10 to 12 years, more than 12 years), dietary energy intake (kcal, quartiles), urinary Na/K ratio and uric acid (mg/dL).
Trend p values were adjusted by FDR(False Discovery Rate) in each qurtile (123 penalties per analysis).
