# Supplementary material for: A population-based urinary and plasma metabolomics study of environmental exposure to cadmium
Source: Environ Health Prev Med. 2024 Mar 30;29:22. doi: 10.1265/ehpm.23-00218 (PMC10992994; doi:10.1265/ehpm.23-00218)
Supplement: Supplementary file 8 — Additional file 8: S Figure 8 Association between urine metabolites and cadmium exposure groups in multvariate regression model in nonsmoker women. [file ehpm-29-022-s008.pdf]

S Figure 8 Association between urine metabolites and cadmium exposure groups in multvariate regression model in nonsmoker women.

Forest plot in multivariate regression model (adjusted model)

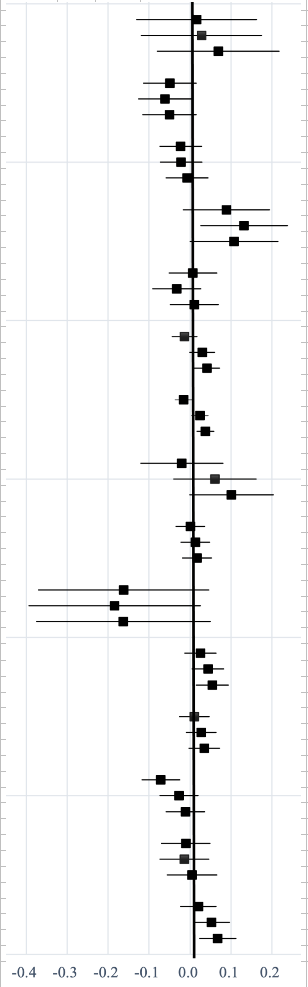

Metabolites	Cd Exposure	Standardized β	95%CI		trend FDR p
	Q2	0.016	-0.130	0.162	
Trimethylamine N-oxide	Q3	0.028	-0.119	0.175	2.56E-01
	Q4	0.069	-0.080	0.218	
	Q2	-0.049	-0.113	0.015	
Ala	Q3	-0.061	-0.125	0.004	3.21E-01
	Q4	-0.050	-0.115	0.016	
	Q2	-0.023	-0.073	0.028	
Choline	Q3	-0.022	-0.073	0.029	5.70E-01
	Q4	-0.007	-0.059	0.044	
	Q2	0.089	-0.017	0.194	
Taurine	Q3	0.132	0.026	0.238	3.83E-02
	Q4	0.108	0.000	0.215	
	Q2	0.007	-0.051	0.065	
1-Methylnicotinamide	Q3	-0.032	-0.091	0.026	5.70E-01
	Q4	0.011	-0.049	0.070	
	Q2	-0.013	-0.044	0.017	
ADMA	Q3	0.030	-0.001	0.060	3.86E-03
	Q4	0.041	0.010	0.072	
	Q2	-0.016	-0.036	0.004	
Uridine	Q3	0.024	0.004	0.044	4.09E-04
	Q4	0.037	0.017	0.058	
	Q2	-0.020	-0.120	0.080	
Adenosine	Q3	0.061	-0.039	0.162	1.55E-02
	Q4	0.101	-0.001	0.203	
	Q2	0.001	-0.034	0.036	
Ethanolamine	Q3	0.013	-0.022	0.048	4.89E-01
	Q4	0.017	-0.018	0.053	
	Q2	-0.162	-0.370	0.046	
Piperidine	Q3	-0.184	-0.393	0.025	2.34E-01
	Q4	-0.163	-0.374	0.049	
	Q2	0.026	-0.012	0.064	
5-Aminolevulinate	Q3	0.044	0.005	0.082	1.63E-03
	Q4	0.054	0.016	0.093	
	Q2	0.011	-0.026	0.047	
Allantoin	Q3	0.027	-0.009	0.063	2.14E-02
	Q4	0.035	-0.002	0.072	
	Q2	-0.071	-0.118	-0.025	
7-Methylguanine	Q3	-0.026	-0.073	0.020	2.85E-02
	Q4	-0.011	-0.059	0.036	
	Q2	-0.010	-0.069	0.049	
N1-Acetylspermidine	Q3	-0.014	-0.073	0.046	7.75E-01
	Q4	0.005	-0.056	0.065	
	Q2	0.021	-0.023	0.064	
N8-Acetylspermidine	Q3	0.052	0.009	0.096	3.75E-04
	Q4	0.068	0.024	0.112	

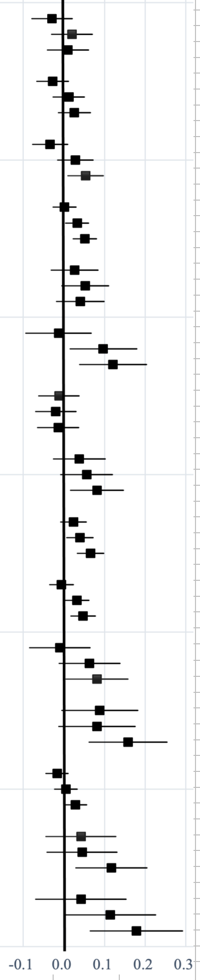

Metabolites	Cd Exposure	Standardized β	95%CI		trend FDR p
	Q2	-0.029	-0.079	0.021	
Gly-Leu	Q3	0.020	-0.030	0.070	1.38E-01
	Q4	0.010	-0.041	0.061	
	Q2	-0.028	-0.067	0.011	
N-epsilon-Acetyllysine	Q3	0.012	-0.027	0.051	1.63E-02
	Q4	0.026	-0.014	0.066	
	Q2	-0.034	-0.077	0.009	
SAM+	Q3	0.028	-0.015	0.072	1.41E-04
	Q4	0.053	0.009	0.098	
	Q2	0.002	-0.027	0.030	
1-Methyladenosine	Q3	0.033	0.004	0.061	2.23E-03
	Q4	0.052	0.023	0.080	
	Q2	0.027	-0.031	0.084	
Lactate	Q3	0.053	-0.005	0.111	1.98E-01
	Q4	0.040	-0.018	0.099	
	Q2	-0.013	-0.093	0.068	
Malonate	Q3	0.096	0.015	0.178	7.13E-05
	Q4	0.121	0.039	0.204	
	Q2	-0.012	-0.062	0.037	
4-Oxopentanoate	Q3	-0.020	-0.070	0.030	8.59E-01
	Q4	-0.014	-0.065	0.037	
	Q2	0.038	-0.026	0.102	
Succinate	Q3	0.056	-0.008	0.120	6.69E-04
	Q4	0.082	0.017	0.147	
	Q2	0.024	-0.008	0.056	
Isethionate	Q3	0.040	0.007	0.072	1.71E-03
	Q4	0.066	0.033	0.098	
	Q2	-0.006	-0.035	0.024	
5-Oxoproline	Q3	0.032	0.002	0.062	9.43E-02
	Q4	0.047	0.017	0.077	
	Q2	-0.010	-0.084	0.065	
Glutarate	Q3	0.063	-0.012	0.138	1.43E-01
	Q4	0.081	0.005	0.158	
	Q2	0.088	-0.005	0.182	
Malate	Q3	0.082	-0.013	0.176	1.29E-03
	Q4	0.158	0.062	0.254	
	Q2	-0.017	-0.044	0.011	
Threonate	Q3	0.005	-0.023	0.033	4.63E-03
	Q4	0.029	0.000	0.057	
	Q2	0.042	-0.044	0.128	
Ethanolamine phosphate	Q3	0.045	-0.041	0.131	2.98E-03
	Q4	0.117	0.029	0.205	
	Q2	0.042	-0.069	0.154	
2-Oxoglutarate	Q3	0.114	0.002	0.226	8.83E-04
	Q4	0.178	0.064	0.292	

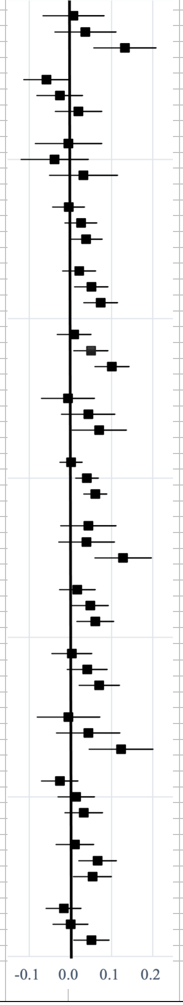

Metabolites	Cd Exposure	Standardized β	95%CI		trend FDR p
	Q2	0.008	-0.066	0.082	
Pimelate	Q3	0.037	-0.038	0.111	5.72E-04
	Q4	0.132	0.057	0.207	
	Q2	-0.058	-0.113	-0.002	
Urate	Q3	-0.026	-0.081	0.030	5.31E-04
	Q4	0.020	-0.037	0.076	
	Q2	-0.005	-0.085	0.076	
Glycerophosphate	Q3	-0.038	-0.119	0.043	2.98E-01
	Q4	0.032	-0.051	0.114	
	Q2	-0.004	-0.043	0.035	
trans-Aconitate	Q3	0.026	-0.014	0.065	1.36E-01
	Q4	0.038	-0.002	0.077	
	Q2	0.021	-0.019	0.061	
cis-Aconitate	Q3	0.051	0.010	0.091	1.55E-03
	Q4	0.073	0.033	0.114	
	Q2	0.009	-0.031	0.050	
N-Acetylaspartate	Q3	0.050	0.009	0.091	5.05E-07
	Q4	0.101	0.059	0.143	
	Q2	-0.006	-0.070	0.059	
Azelate	Q3	0.043	-0.021	0.108	2.71E-02
	Q4	0.070	0.004	0.136	
	Q2	0.002	-0.026	0.029	
Isocitrate	Q3	0.040	0.013	0.068	2.95E-06
	Q4	0.060	0.032	0.088	
	Q2	0.044	-0.024	0.111	
Adipate	Q3	0.039	-0.028	0.107	1.64E-03
	Q4	0.128	0.059	0.197	
	Q2	0.017	-0.027	0.061	
3-Hydroxy-3-methylglutarate	Q3	0.048	0.004	0.092	7.23E-04
	Q4	0.061	0.016	0.105	
	Q2	0.004	-0.045	0.052	
2,3-Pyridinedicarboxylate	Q3	0.041	-0.008	0.089	2.17E-03
	Q4	0.070	0.021	0.119	
	Q2	-0.005	-0.081	0.072	
Suberate	Q3	0.043	-0.033	0.120	2.91E-03
	Q4	0.123	0.045	0.201	
	Q2	-0.025	-0.070	0.019	
Gluconate	Q3	0.014	-0.030	0.059	4.01E-02
	Q4	0.033	-0.013	0.078	
	Q2	0.011	-0.034	0.057	
Saccharate	Q3	0.066	0.020	0.112	1.34E-02
	Q4	0.054	0.007	0.100	
	Q2	-0.016	-0.058	0.026	
N-Acetylneuraminate	Q3	0.001	-0.042	0.043	2.11E-02
	Q4	0.051	0.008	0.095	

Multivariate regression model was adjusted by age (years),sex ,systolic blood pressure (mmHg), smoke (pack-year), rice intake(bowl/week), BMI (kg/m2), HbA1c (%), LDLc (mg/dL), alcohol consumption (ethanol intake: g/day), physical activity (METs, quartiles), educational history (less than 10 years, 10 to 12 years, more than 12 years), dietary energy intake (kcal, quartiles), urinary Na/K ratio and uric acid (mg/dL).
Trend p values were adjusted by FDR(False Discovery Rate) in each qurtile (123 penalties per analysis).
