# Supplementary material for: A population-based urinary and plasma metabolomics study of environmental exposure to cadmium
Source: Environ Health Prev Med. 2024 Mar 30;29:22. doi: 10.1265/ehpm.23-00218 (PMC10992994; doi:10.1265/ehpm.23-00218)
Supplement: Supplementary file 10 — Additional file 10: S Figure 10 Association between plasma metabolites and cadmium exposure groups in sex stratification. [file ehpm-29-022-s010.pdf]

S Figure 10 Association between plasma metabolites and cadmium exposure groups in sex stratification.

Men Variables

Forest plot in univariate regression model (adjusted model)

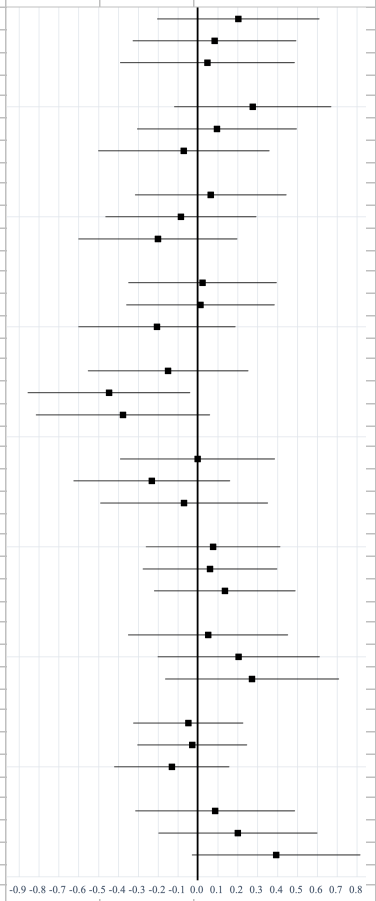

Metabolites	Cd Exposure	Standardized β	95%CI		trend FDR p
Trimethylamine N-oxide	Q2	0.202	-0.204	0.608	
	Q3	0.083	-0.327	0.493	8.91E-01
	Q4	0.047	-0.391	0.485	
Ala	Q2	0.275	-0.119	0.669	
	Q3	0.094	-0.306	0.495	3.22E-01
	Q4	-0.072	-0.501	0.358	
Sarcosine	Q2	0.063	-0.316	0.443	
	Q3	-0.087	-0.465	0.292	1.90E-01
	Q4	-0.202	-0.601	0.196	
N,N-Dimethylglycine	Q2	0.022	-0.350	0.393	
	Q3	0.011	-0.360	0.383	4.67E-01
	Q4	-0.207	-0.601	0.187	
Creatinine	Q2	-0.151	-0.553	0.252	
	Q3	-0.448	-0.856	-0.041	7.96E-02
	Q4	-0.378	-0.815	0.058	
Ile	Q2	-0.003	-0.391	0.385	
	Q3	-0.233	-0.626	0.160	5.23E-01
	Q4	-0.070	-0.491	0.350	
Asp	Q2	0.075	-0.262	0.413	
	Q3	0.060	-0.277	0.396	8.34E-01
	Q4	0.135	-0.220	0.489	
Carnitine	Q2	0.050	-0.350	0.451	
	Q3	0.204	-0.202	0.610	1.19E-01
	Q4	0.271	-0.165	0.707	
Uridine	Q2	-0.050	-0.325	0.226	
	Q3	-0.029	-0.304	0.245	9.83E-01
	Q4	-0.132	-0.420	0.156	
2-Oxoglutarate	Q2	0.086	-0.315	0.486	
	Q3	0.200	-0.198	0.599	8.79E-02
	Q4	0.392	-0.030	0.815	

Women Variables

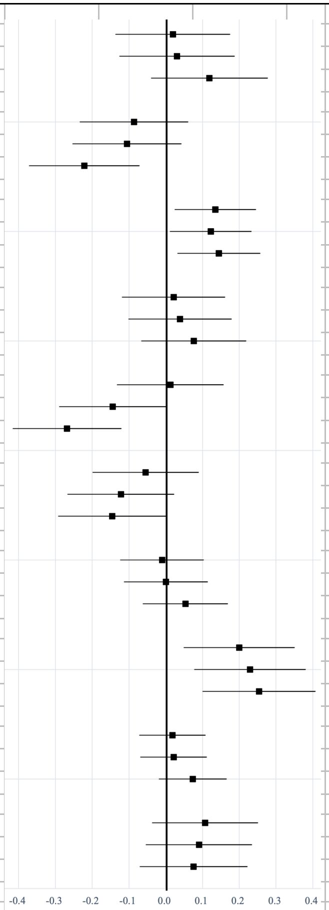

Metabolites	Cd Exposure	Standardized β	95%CI		trend FDR p
Trimethylamine N-oxide	Q2	0.020	-0.136	0.175	
	Q3	0.031	-0.125	0.187	1.05E-01
	Q4	0.119	-0.039	0.277	
Ala	Q2	-0.086	-0.233	0.061	
	Q3	-0.105	-0.252	0.042	7.76E-03
	Q4	-0.222	-0.371	-0.072	
Sarcosine	Q2	0.135	0.025	0.245	
	Q3	0.122	0.012	0.233	5.59E-03
	Q4	0.145	0.033	0.257	
N,N-Dimethylglycine	Q2	0.021	-0.118	0.161	
	Q3	0.039	-0.101	0.179	6.82E-03
	Q4	0.076	-0.066	0.218	
Creatinine	Q2	0.012	-0.132	0.157	
	Q3	-0.145	-0.289	0.000	4.78E-04
	Q4	-0.268	-0.415	-0.121	
Ile	Q2	-0.054	-0.198	0.089	
	Q3	-0.122	-0.266	0.022	3.09E-02
	Q4	-0.145	-0.292	0.001	
Asp	Q2	-0.010	-0.123	0.103	
	Q3	0.000	-0.113	0.114	1.23E-03
	Q4	0.054	-0.061	0.169	
Carnitine	Q2	0.200	0.050	0.350	
	Q3	0.229	0.079	0.380	1.06E-03
	Q4	0.254	0.101	0.407	
Uridine	Q2	0.018	-0.071	0.108	
	Q3	0.021	-0.068	0.111	3.98E-04
	Q4	0.074	-0.018	0.165	
2-Oxoglutarate	Q2	0.107	-0.036	0.250	
	Q3	0.090	-0.053	0.234	1.28E-01
	Q4	0.076	-0.070	0.222	

Multivariate regression model was adjusted by age (years), sex, systolic blood pressure (mmHg), smoke (pack-year), rice intake(bowl/week), BMI (kg/m2), HbA1c (%), LDLc (mg/dL), alcohol consumption (ethanol intake: g/day), physical activity (METs, quartiles), educational history (less than 10 years, 10 to 12 years, more than 12 years), dietary energy intake (kcal, quartiles), urinary Na/K ratio and uric acid (mg/dL).

P values were adjusted by FDR(False Discovery Rate) in each qurtile (94 penalties per analysis).
