# Supplementary material for: A population-based urinary and plasma metabolomics study of environmental exposure to cadmium
Source: Environ Health Prev Med. 2024 Mar 30;29:22. doi: 10.1265/ehpm.23-00218 (PMC10992994; doi:10.1265/ehpm.23-00218)
Supplement: Supplementary file 11 — Additional file 11: S Figure 11 Association between urine metabolites and cadmium exposure groups excluding participants for urinary creatinine >3.0 g/L or <0.3 g/L. [file ehpm-29-022-s011.pdf]

S Figure 11 Association between urine metabolites and cadmium exposure groups excluding participants for urinary creatinine >3.0 g / L or <0.3 g / L.

Forest plot in multivariate regression model (adjusted model)

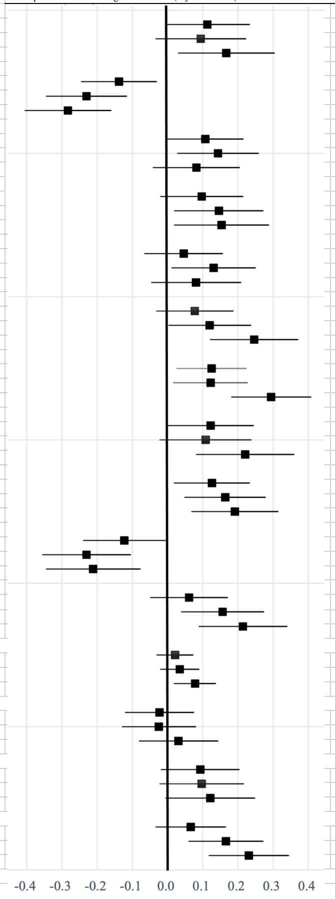

Metabolites	Cd Exposure	Standardized β	95%CI		trend FDR p
Trimethylamine N-oxide	Q2	0.113	-0.006	0.232	
	Q3	0.094	-0.034	0.222	9.56E-03
	Q4	0.167	0.030	0.303	
Ala	Q2	-0.138	-0.245	-0.031	
	Q3	-0.231	-0.345	-0.116	1.09E-04
	Q4	-0.283	-0.406	-0.161	
Choline	Q2	0.107	-0.001	0.214	
	Q3	0.143	0.028	0.258	1.38E-02
	Q4	0.082	-0.041	0.205	
Taurine	Q2	0.097	-0.020	0.214	
	Q3	0.146	0.020	0.271	1.52E-02
	Q4	0.153	0.019	0.288	
1-Methylnicotinamide	Q2	0.046	-0.065	0.157	
	Q3	0.131	0.012	0.250	5.04E-03
	Q4	0.081	-0.046	0.208	
ADMA	Q2	0.077	-0.031	0.186	
	Q3	0.120	0.004	0.236	4.81E-04
	Q4	0.246	0.121	0.370	
Uridine	Q2	0.125	0.026	0.223	
	Q3	0.122	0.017	0.227	2.37E-03
	Q4	0.294	0.182	0.407	
Adenosine	Q2	0.122	0.001	0.244	
	Q3	0.108	-0.022	0.238	3.91E-03
	Q4	0.221	0.082	0.360	
Ethanolamine	Q2	0.126	0.019	0.233	
	Q3	0.164	0.049	0.278	3.70E-03
	Q4	0.191	0.069	0.314	
Piperidine	Q2	-0.123	-0.239	-0.006	
	Q3	-0.230	-0.355	-0.105	6.55E-03
	Q4	-0.211	-0.345	-0.078	
5-Aminolevulinate	Q2	0.061	-0.048	0.170	
	Q3	0.156	0.039	0.274	1.24E-04
	Q4	0.214	0.089	0.339	
Allantoin	Q2	0.021	-0.031	0.072	
	Q3	0.035	-0.021	0.090	4.27E-02
	Q4	0.077	0.018	0.136	
7-Methylguanine	Q2	-0.023	-0.121	0.075	
	Q3	-0.024	-0.129	0.080	6.94E-04
	Q4	0.031	-0.080	0.143	
N1-Acetylspermidine	Q2	0.093	-0.018	0.204	
	Q3	0.097	-0.022	0.215	2.57E-02
	Q4	0.121	-0.005	0.248	
N8-Acetylspermidine	Q2	0.066	-0.033	0.165	
	Q3	0.166	0.060	0.272	6.31E-06
	Q4	0.231	0.117	0.344	

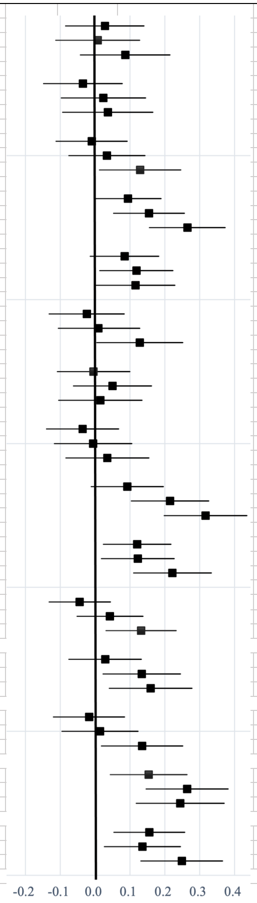

Metabolites	Cd Exposure	Standardized β	95%CI		trend FDR p
Gly-Leu	Q2	0.028	-0.085	0.140	
	Q3	0.007	-0.113	0.128	2.28E-02
	Q4	0.086	-0.043	0.215	
N-epsilon-Acetyllysine	Q2	-0.035	-0.148	0.078	
	Q3	0.023	-0.098	0.145	5.08E-02
	Q4	0.036	-0.094	0.166	
SAM+	Q2	-0.010	-0.112	0.092	
	Q3	0.034	-0.076	0.143	4.91E-05
	Q4	0.129	0.012	0.246	
1-Methyladenosine	Q2	0.094	-0.002	0.189	
	Q3	0.155	0.053	0.256	3.18E-05
	Q4	0.265	0.156	0.374	
Lactate	Q2	0.084	-0.014	0.182	
	Q3	0.118	0.013	0.224	1.86E-02
	Q4	0.116	0.003	0.229	
Malonate	Q2	-0.024	-0.132	0.084	
	Q3	0.010	-0.106	0.125	2.98E-03
	Q4	0.128	0.004	0.252	
4-Oxopentanoate	Q2	-0.005	-0.109	0.100	
	Q3	0.050	-0.062	0.162	1.69E-01
	Q4	0.014	-0.106	0.134	
Succinate	Q2	-0.036	-0.140	0.068	
	Q3	-0.006	-0.118	0.105	6.53E-02
	Q4	0.035	-0.084	0.154	
Isethionate	Q2	0.093	-0.011	0.196	
	Q3	0.214	0.103	0.326	1.88E-06
	Q4	0.317	0.198	0.436	
5-Oxoproline	Q2	0.120	0.023	0.218	
	Q3	0.122	0.018	0.227	3.21E-02
	Q4	0.222	0.110	0.333	
Glutarate	Q2	-0.044	-0.132	0.044	
	Q3	0.043	-0.052	0.137	4.93E-03
	Q4	0.132	0.031	0.233	
Malate	Q2	0.028	-0.075	0.132	
	Q3	0.134	0.022	0.245	2.02E-03
	Q4	0.159	0.040	0.278	
Threonate	Q2	-0.018	-0.120	0.084	
	Q3	0.014	-0.095	0.124	2.95E-03
	Q4	0.135	0.018	0.252	
Ethanolamine phosphate	Q2	0.153	0.043	0.264	
	Q3	0.264	0.146	0.382	8.19E-06
	Q4	0.244	0.118	0.370	
2-Oxoglutarate	Q2	0.155	0.053	0.258	
	Q3	0.136	0.026	0.245	2.36E-04
	Q4	0.248	0.131	0.366	

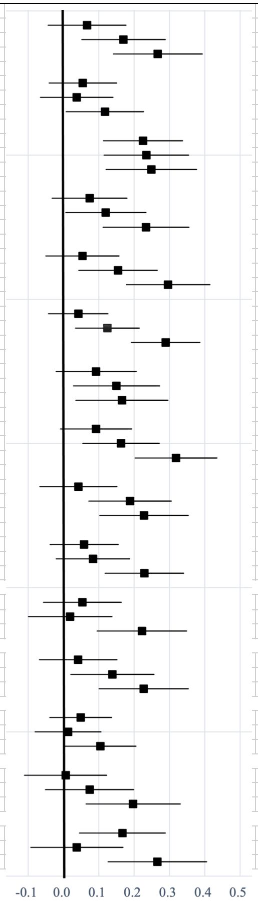

Metabolites	Cd Exposure	Standardized β	95%CI		trend FDR p
Pimelate	Q2	0.067	-0.043	0.177	
	Q3	0.170	0.052	0.288	1.38E-05
	Q4	0.267	0.141	0.393	
Urate	Q2	0.055	-0.041	0.151	
	Q3	0.038	-0.065	0.141	3.30E-05
	Q4	0.118	0.007	0.228	
Glycerophosphate	Q2	0.225	0.114	0.337	
	Q3	0.235	0.115	0.355	1.23E-04
	Q4	0.249	0.121	0.377	
trans-Aconitate	Q2	0.074	-0.032	0.180	
	Q3	0.120	0.007	0.234	9.98E-04
	Q4	0.234	0.113	0.356	
cis-Aconitate	Q2	0.054	-0.050	0.157	
	Q3	0.155	0.044	0.266	8.95E-07
	Q4	0.297	0.178	0.416	
N-Acetylaspartate	Q2	0.042	-0.042	0.127	
	Q3	0.124	0.034	0.215	2.34E-09
	Q4	0.290	0.193	0.386	
Azelate	Q2	0.093	-0.021	0.207	
	Q3	0.151	0.028	0.273	1.70E-02
	Q4	0.166	0.035	0.297	
Isocitrate	Q2	0.093	-0.008	0.194	
	Q3	0.164	0.055	0.272	1.31E-08
	Q4	0.319	0.203	0.435	
Adipate	Q2	0.043	-0.067	0.152	
	Q3	0.189	0.072	0.306	9.85E-05
	Q4	0.228	0.103	0.354	
3-Hydroxy-3-methylglutarate	Q2	0.059	-0.038	0.156	
	Q3	0.084	-0.020	0.188	1.99E-05
	Q4	0.229	0.118	0.341	
2,3-Pyridinedicarboxylate	Q2	0.054	-0.056	0.165	
	Q3	0.019	-0.099	0.138	1.77E-03
	Q4	0.222	0.096	0.349	
Suberate	Q2	0.042	-0.068	0.152	
	Q3	0.139	0.021	0.257	2.86E-04
	Q4	0.228	0.101	0.354	
Gluconate	Q2	0.049	-0.038	0.137	
	Q3	0.013	-0.081	0.107	1.23E-02
	Q4	0.105	0.005	0.206	
Saccharate	Q2	0.006	-0.111	0.123	3.26E-03
	Q3	0.074	-0.051	0.199	
	Q4	0.198	0.064	0.332	
N-Acetylneuraminate	Q2	0.167	0.045	0.288	5.82E-03
	Q3	0.038	-0.093	0.169	
	Q4	0.266	0.126	0.406	

Multivariate regression model was adjusted by age (years),sex ,systolic blood pressure (mmHg), smoke (pack-year), rice intake(bowl/week), BMI (kg/m2), HbA1c (%), LDLc (mg/dL), alcohol consumption (ethanol intake: g/day), physical activity (METs, quartiles), educational history (less than 10 years, 10 to 12 years, more than 12 years), dietary energy intake (kcal, quartiles), urinary Na/K ratio and uric acid (mg/dL).
Trend p values were adjusted by FDR(False Discovery Rate) in each qurtile (123 penalties per analysis).
