# Supplementary material for: A population-based urinary and plasma metabolomics study of environmental exposure to cadmium
Source: Environ Health Prev Med. 2024 Mar 30;29:22. doi: 10.1265/ehpm.23-00218 (PMC10992994; doi:10.1265/ehpm.23-00218)
Supplement: Supplementary file 12 — Additional file 12: S Figure 12 Association between urine metabolites and cadmium exposure groups excluding participants for urinary creatinine >3.0 g/L or <0.3 g/L in men. [file ehpm-29-022-s012.pdf]

S Figure 12 Association between urine metabolites and cadmium exposure groups excluding participants for urinary creatinine >3.0 g / L or <0.3 g / L in men.

Forest plot in multivariate regression model (adjusted model)

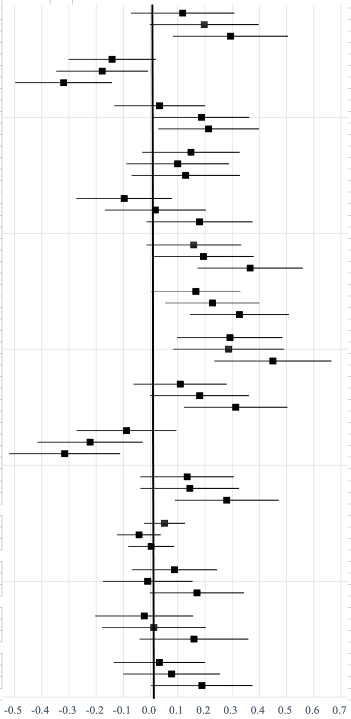

Metabolites	Cd Exposure	Standardized β	95%CI		trend FDR p
	Q2	0.118	-0.071	0.307	
Trimethylamine N-oxide	Q3	0.197	-0.002	0.396	5.41E-03
	Q4	0.294	0.084	0.505	
	Q2	-0.141	-0.301	0.018	
Ala	Q3	-0.178	-0.346	-0.010	1.15E-03
	Q4	-0.319	-0.496	-0.142	
	Q2	0.033	-0.132	0.199	
Choline	Q3	0.187	0.012	0.362	5.58E-03
	Q4	0.213	0.029	0.397	
	Q2	0.148	-0.030	0.327	
Taurine	Q3	0.100	-0.088	0.288	2.29E-01
	Q4	0.129	-0.069	0.327	
	Q2	-0.097	-0.272	0.078	
1-Methylnicotinamide	Q3	0.018	-0.167	0.203	3.85E-03
	Q4	0.180	-0.015	0.374	
	Q2	0.159	-0.014	0.333	
ADMA	Q3	0.195	0.011	0.378	2.27E-04
	Q4	0.366	0.174	0.559	
	Q2	0.167	0.004	0.329	
Uridine	Q3	0.227	0.055	0.399	7.72E-03
	Q4	0.326	0.145	0.507	
	Q2	0.292	0.099	0.485	
Adenosine	Q3	0.287	0.084	0.490	2.71E-04
	Q4	0.450	0.235	0.664	
	Q2	0.109	-0.062	0.279	
Ethanolamine	Q3	0.181	0.000	0.361	8.93E-04
	Q4	0.313	0.123	0.503	
	Q2	-0.088	-0.271	0.094	
Piperidine	Q3	-0.222	-0.414	-0.030	1.14E-03
	Q4	-0.315	-0.518	-0.112	
	Q2	0.134	-0.037	0.305	
5-Aminolevulinate	Q3	0.144	-0.036	0.325	8.81E-03
	Q4	0.280	0.090	0.470	
	Q2	0.051	-0.024	0.126	
Allantoin	Q3	-0.043	-0.122	0.036	2.46E-02
	Q4	0.002	-0.081	0.086	
	Q2	0.088	-0.067	0.244	
7-Methylguanine	Q3	-0.010	-0.174	0.154	3.88E-03
	Q4	0.171	-0.002	0.343	
	Q2	-0.024	-0.203	0.155	
N1-Acetylspermidine	Q3	0.013	-0.176	0.202	1.01E-01
	Q4	0.160	-0.039	0.359	
	Q2	0.032	-0.135	0.199	
N8-Acetylspermidine	Q3	0.077	-0.099	0.254	5.46E-02
	Q4	0.189	0.003	0.374	

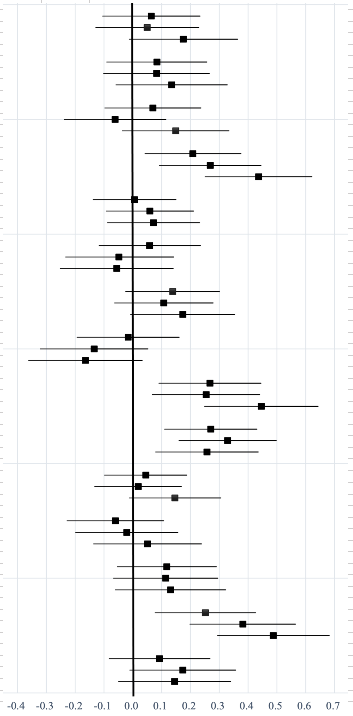

Metabolites	Cd Exposure	Standardized β	95%CI		trend FDR p
	Q2	0.064	-0.105	0.233	
Gly-Leu	Q3	0.050	-0.129	0.229	4.14E-02
	Q4	0.175	-0.013	0.364	
	Q2	0.083	-0.091	0.258	
N-epsilon-Acetyllysine	Q3	0.082	-0.101	0.266	6.99E-02
	Q4	0.134	-0.059	0.328	
	Q2	0.070	-0.097	0.236	
SAM+	Q3	-0.061	-0.238	0.115	2.60E-02
	Q4	0.149	-0.037	0.334	
	Q2	0.208	0.042	0.375	
1-Methyladenosine	Q3	0.269	0.093	0.445	1.05E-05
	Q4	0.436	0.251	0.622	
	Q2	0.006	-0.138	0.150	
Lactate	Q3	0.059	-0.092	0.211	5.68E-02
	Q4	0.072	-0.089	0.232	
	Q2	0.059	-0.118	0.235	
Malonate	Q3	-0.048	-0.234	0.138	5.84E-01
	Q4	-0.056	-0.252	0.140	
	Q2	0.138	-0.025	0.300	
4-Oxopentanoate	Q3	0.108	-0.063	0.279	5.21E-02
	Q4	0.173	-0.008	0.354	
	Q2	-0.016	-0.193	0.161	
Succinate	Q3	-0.134	-0.321	0.052	1.86E-01
	Q4	-0.164	-0.362	0.033	
	Q2	0.268	0.090	0.445	
Isethionate	Q3	0.254	0.067	0.441	1.96E-04
	Q4	0.446	0.249	0.643	
	Q2	0.270	0.110	0.431	
5-Oxoproline	Q3	0.329	0.160	0.498	1.33E-02
	Q4	0.257	0.079	0.436	
	Q2	0.044	-0.098	0.187	
Glutarate	Q3	0.018	-0.132	0.168	6.75E-02
	Q4	0.146	-0.013	0.305	
	Q2	-0.061	-0.229	0.108	
Malate	Q3	-0.021	-0.199	0.156	6.13E-01
	Q4	0.051	-0.136	0.238	
	Q2	0.118	-0.054	0.290	
Threonate	Q3	0.114	-0.068	0.295	8.82E-02
	Q4	0.131	-0.061	0.323	
	Q2	0.252	0.077	0.426	
Ethanolamine phosphate	Q3	0.382	0.198	0.565	3.75E-07
	Q4	0.488	0.294	0.682	
	Q2	0.092	-0.082	0.267	
2-Oxoglutarate	Q3	0.173	-0.011	0.357	2.88E-01
	Q4	0.145	-0.049	0.340	

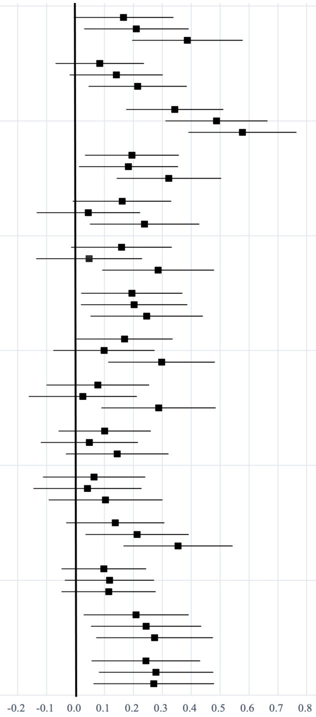

Metabolites	Cd Exposure	Standardized β	95%CI		trend FDR p
	Q2	0.167	-0.004	0.337	
Pimelate	Q3	0.211	0.031	0.391	1.27E-04
	Q4	0.387	0.197	0.577	
	Q2	0.084	-0.068	0.236	
Urate	Q3	0.141	-0.019	0.301	2.77E-04
	Q4	0.215	0.047	0.384	
	Q2	0.343	0.177	0.510	
Glycerophosphate	Q3	0.488	0.312	0.663	1.83E-09
	Q4	0.578	0.392	0.763	
	Q2	0.195	0.034	0.357	
trans-Aconitate	Q3	0.184	0.014	0.353	8.01E-03
	Q4	0.323	0.143	0.503	
	Q2	0.161	-0.008	0.330	
cis-Aconitate	Q3	0.045	-0.133	0.223	8.25E-02
	Q4	0.239	0.051	0.427	
	Q2	0.159	-0.014	0.332	
N-Acetylaspartate	Q3	0.047	-0.135	0.229	3.75E-02
	Q4	0.286	0.093	0.478	
	Q2	0.195	0.021	0.369	
Azelate	Q3	0.203	0.020	0.386	2.94E-02
	Q4	0.247	0.053	0.440	
	Q2	0.170	0.004	0.335	
Isocitrate	Q3	0.099	-0.075	0.273	6.11E-03
	Q4	0.298	0.114	0.482	
	Q2	0.077	-0.099	0.254	
Adipate	Q3	0.025	-0.161	0.211	1.56E-02
	Q4	0.288	0.091	0.484	
	Q2	0.101	-0.058	0.259	
3-Hydroxy-3-methylglutarate	Q3	0.048	-0.118	0.215	2.73E-01
	Q4	0.145	-0.032	0.321	
	Q2	0.065	-0.112	0.241	
2,3-Pyridinedicarboxylate	Q3	0.042	-0.144	0.227	3.79E-01
	Q4	0.104	-0.092	0.300	
	Q2	0.138	-0.031	0.307	
Suberate	Q3	0.213	0.036	0.391	2.03E-04
	Q4	0.354	0.167	0.542	
	Q2	0.098	-0.047	0.243	
Gluconate	Q3	0.118	-0.035	0.271	7.08E-02
	Q4	0.115	-0.047	0.277	
	Q2	0.210	0.029	0.390	
Saccharate	Q3	0.244	0.054	0.434	9.85E-03
	Q4	0.273	0.073	0.474	
	Q2	0.243	0.057	0.430	
N-Acetylneuraminate	Q3	0.279	0.082	0.475	2.44E-02
	Q4	0.271	0.064	0.478	

Multivariate regression model was adjusted by age (years),sex ,systolic blood pressure (mmHg), smoke (pack-year), rice intake(bowl/week), BMI (kg/m2), HbA1c (%), LDLc (mg/dL), alcohol consumption (ethanol intake: g/day), physical activity (METs, quartiles), educational history (less than 10 years, 10 to 12 years, more than 12 years), dietary energy intake (kcal, quartiles), urinary Na/K ratio and uric acid (mg/dL).
Trend p values were adjusted by FDR(False Discovery Rate) in each qurtile (123 penalties per analysis).
