# Supplementary material for: A population-based urinary and plasma metabolomics study of environmental exposure to cadmium
Source: Environ Health Prev Med. 2024 Mar 30;29:22. doi: 10.1265/ehpm.23-00218 (PMC10992994; doi:10.1265/ehpm.23-00218)
Supplement: Supplementary file 13 — Additional file 13: S Figure 13 Association between urine metabolites and cadmium exposure groups excluding participants for urinary creatinine >3.0 g/L or <0.3 g/L in women. [file ehpm-29-022-s013.pdf]

S Figure 13 Association between urine metabolites and cadmium exposure groups excluding participants for urinary creatinine >3.0 g / L or <0.3 g / L in women.

Forest plot in multivariate regression model (adjusted model)

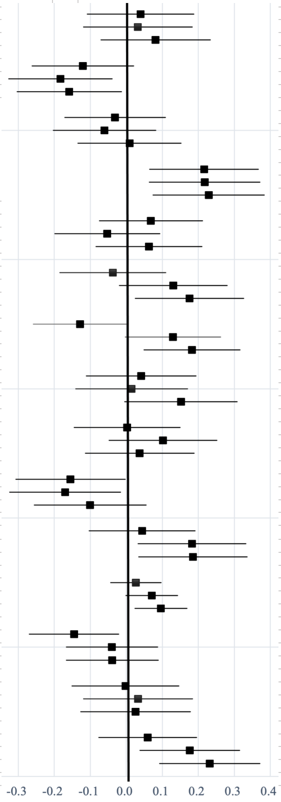

Metabolites	Cd Exposure	Standardized β	95%CI		trend FDR p
Trimethylamine N-oxide	Q2	0.039	-0.109	0.187	1.90E-01
	Q3	0.032	-0.119	0.183	
	Q4	0.081	-0.071	0.233	
Ala	Q2	-0.121	-0.262	0.020	8.42E-02
	Q3	-0.183	-0.327	-0.040	
	Q4	-0.159	-0.304	-0.014	
Choline	Q2	-0.031	-0.171	0.108	2.57E-01
	Q3	-0.061	-0.203	0.082	
	Q4	0.009	-0.135	0.152	
Taurine	Q2	0.216	0.065	0.366	6.94E-03
	Q3	0.218	0.064	0.371	
	Q4	0.229	0.074	0.384	
1-Methylnicotinamide	Q2	0.068	-0.075	0.211	2.38E-01
	Q3	-0.053	-0.199	0.093	
	Q4	0.063	-0.084	0.210	
ADMA	Q2	-0.038	-0.184	0.109	7.20E-03
	Q3	0.130	-0.019	0.280	
	Q4	0.175	0.025	0.326	
Uridine	Q2	-0.129	-0.259	0.001	4.02E-03
	Q3	0.129	-0.003	0.262	
	Q4	0.182	0.049	0.316	
Adenosine	Q2	0.041	-0.111	0.194	7.33E-02
	Q3	0.015	-0.141	0.170	
	Q4	0.152	-0.005	0.308	
Ethanolamine	Q2	0.002	-0.145	0.149	4.70E-01
	Q3	0.102	-0.048	0.251	
	Q4	0.037	-0.114	0.188	
Piperidine	Q2	-0.155	-0.307	-0.003	4.29E-01
	Q3	-0.170	-0.325	-0.016	
	Q4	-0.100	-0.256	0.055	
5-Aminolevulinate	Q2	0.044	-0.103	0.191	9.72E-04
	Q3	0.182	0.033	0.332	
	Q4	0.185	0.034	0.336	
Allantoin	Q2	0.027	-0.044	0.097	2.66E-01
	Q3	0.071	-0.001	0.142	
	Q4	0.096	0.024	0.168	
7-Methylguanine	Q2	-0.145	-0.270	-0.021	3.84E-02
	Q3	-0.040	-0.167	0.087	
	Q4	-0.039	-0.167	0.089	
N1-Acetylspermidine	Q2	-0.002	-0.151	0.146	3.45E-01
	Q3	0.033	-0.119	0.184	
	Q4	0.026	-0.127	0.178	
N8-Acetylspermidine	Q2	0.060	-0.076	0.195	2.12E-05
	Q3	0.176	0.038	0.315	
	Q4	0.232	0.092	0.371	

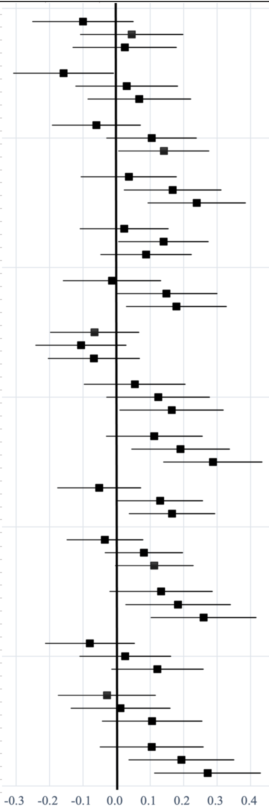

Metabolites	Cd Exposure	Standardized β	95%CI		trend FDR p
Gly-Leu	Q2	-0.100	-0.250	0.050	1.06E-01
	Q3	0.045	-0.108	0.198	
	Q4	0.025	-0.130	0.179	
N-epsilon-Acetyllysine	Q2	-0.158	-0.307	-0.009	1.89E-02
	Q3	0.030	-0.122	0.182	
	Q4	0.068	-0.085	0.221	
SAM+	Q2	-0.060	-0.192	0.071	5.70E-04
	Q3	0.105	-0.029	0.238	
	Q4	0.141	0.006	0.276	
1-Methyladenosine	Q2	0.036	-0.106	0.179	3.17E-03
	Q3	0.167	0.023	0.312	
	Q4	0.239	0.093	0.385	
Lactate	Q2	0.023	-0.109	0.154	8.55E-02
	Q3	0.140	0.006	0.274	
	Q4	0.088	-0.047	0.223	
Malonate	Q2	-0.014	-0.159	0.131	8.92E-04
	Q3	0.148	0.000	0.296	
	Q4	0.178	0.029	0.327	
4-Oxopentanoate	Q2	-0.065	-0.198	0.067	6.26E-01
	Q3	-0.106	-0.241	0.028	
	Q4	-0.068	-0.203	0.068	
Succinate	Q2	0.054	-0.096	0.204	3.03E-03
	Q3	0.124	-0.029	0.278	
	Q4	0.164	0.010	0.319	
Isethionate	Q2	0.112	-0.031	0.255	1.90E-03
	Q3	0.191	0.045	0.337	
	Q4	0.287	0.140	0.434	
5-Oxoproline	Q2	-0.052	-0.175	0.072	1.78E-01
	Q3	0.130	0.004	0.257	
	Q4	0.165	0.038	0.293	
Glutarate	Q2	-0.035	-0.148	0.079	2.28E-01
	Q3	0.082	-0.034	0.197	
	Q4	0.113	-0.004	0.229	
Malate	Q2	0.132	-0.020	0.285	3.18E-04
	Q3	0.184	0.028	0.339	
	Q4	0.260	0.103	0.417	
Threonate	Q2	-0.080	-0.213	0.053	1.37E-02
	Q3	0.026	-0.110	0.161	
	Q4	0.122	-0.015	0.258	
Ethanolamine phosphate	Q2	-0.029	-0.174	0.116	5.26E-02
	Q3	0.012	-0.136	0.160	
	Q4	0.106	-0.043	0.255	
2-Oxoglutarate	Q2	0.105	-0.049	0.258	4.09E-04
	Q3	0.193	0.037	0.350	
	Q4	0.271	0.113	0.429	

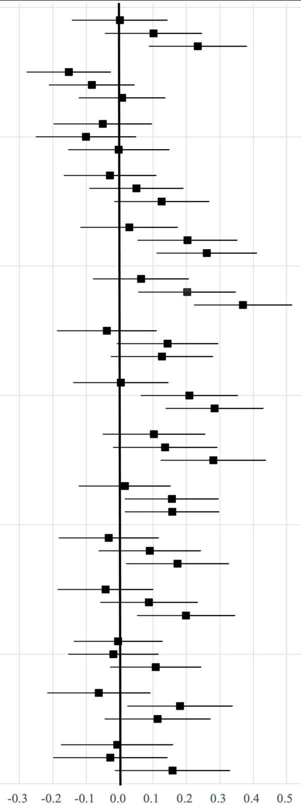

Metabolites	Cd Exposure	Standardized β	95%CI		trend FDR p
Pimelate	Q2	0.001	-0.141	0.143	5.73E-04
	Q3	0.102	-0.043	0.246	
	Q4	0.234	0.088	0.380	
Urate	Q2	-0.152	-0.277	-0.027	6.54E-03
	Q3	-0.083	-0.211	0.044	
	Q4	0.007	-0.121	0.136	
Glycerophosphate	Q2	-0.050	-0.197	0.096	6.98E-01
	Q3	-0.101	-0.250	0.049	
	Q4	-0.003	-0.153	0.148	
trans-Aconitate	Q2	-0.029	-0.166	0.109	1.29E-01
	Q3	0.050	-0.090	0.190	
	Q4	0.126	-0.015	0.267	
cis-Aconitate	Q2	0.028	-0.117	0.174	2.43E-04
	Q3	0.204	0.055	0.352	
	Q4	0.261	0.111	0.410	
N-Acetylaspartate	Q2	0.064	-0.078	0.206	2.40E-07
	Q3	0.202	0.057	0.347	
	Q4	0.369	0.224	0.515	
Azelate	Q2	-0.039	-0.187	0.110	3.38E-02
	Q3	0.143	-0.008	0.294	
	Q4	0.127	-0.026	0.279	
Isocitrate	Q2	0.004	-0.138	0.146	1.49E-05
	Q3	0.209	0.064	0.353	
	Q4	0.284	0.138	0.430	
Adipate	Q2	0.103	-0.050	0.255	1.14E-03
	Q3	0.136	-0.019	0.292	
	Q4	0.281	0.124	0.437	
3-Hydroxy-3-methylglutarate	Q2	0.015	-0.121	0.152	1.57E-03
	Q3	0.156	0.017	0.295	
	Q4	0.157	0.017	0.297	
2,3-Pyridinedicarboxylate	Q2	-0.032	-0.181	0.117	7.43E-03
	Q3	0.090	-0.061	0.242	
	Q4	0.174	0.021	0.327	
Suberate	Q2	-0.042	-0.184	0.100	5.11E-03
	Q3	0.088	-0.057	0.233	
	Q4	0.199	0.053	0.345	
Gluconate	Q2	-0.004	-0.136	0.127	1.86E-01
	Q3	-0.019	-0.153	0.115	
	Q4	0.108	-0.027	0.244	
Saccharate	Q2	-0.062	-0.216	0.091	6.47E-02
	Q3	0.181	0.025	0.337	
	Q4	0.114	-0.044	0.272	
N-Acetylneuraminate	Q2	-0.007	-0.174	0.160	1.26E-01
	Q3	-0.028	-0.198	0.142	
	Q4	0.159	-0.013	0.330	

Multivariate regression model was adjusted by age (years),sex ,systolic blood pressure (mmHg), smoke (pack-year), rice intake(bowl/week), BMI (kg/m2), HbA1c (%), LDLc (mg/dL), alcohol consumption (ethanol intake: g/day), physical activity (METs, quartiles), educational history (less than 10 years, 10 to 12 years, more than 12 years), dietary energy intake (kcal, quartiles), urinary Na/K ratio and uric acid (mg/dL).
Trend p values were adjusted by FDR(False Discovery Rate) in each qurtile (123 penalties per analysis).
