# Supplementary material for: A population-based urinary and plasma metabolomics study of environmental exposure to cadmium
Source: Environ Health Prev Med. 2024 Mar 30;29:22. doi: 10.1265/ehpm.23-00218 (PMC10992994; doi:10.1265/ehpm.23-00218)
Supplement: Supplementary file 15 — Additional file 15: S Figure 15 Association between plasma metabolites and cadmium exposure groups excluding participants for urinary creatinine >3.0 g/L or <0.3 g/L in sex stratification. [file ehpm-29-022-s015.pdf]

S Figure 15 Association between plasma metabolites and cadmium exposure groups excluding participants for urinary creatinine >3.0 g / L or <0.3 g / L in sex stratification.

Men Variables

Forest plot in univariate regression model (adjusted model)

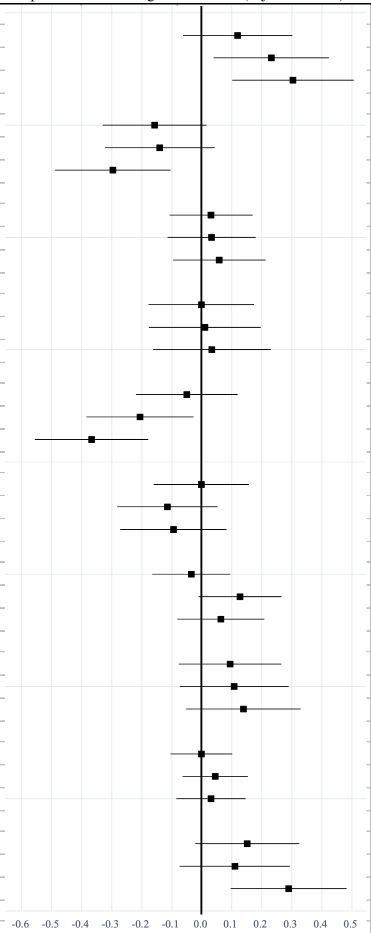

Metabolites	Cd Exposure	Standardized β	95%CI		trend FDR p
Trimethylamine N-oxide	Q2	0.119	-0.062	0.300	8.85E-04
	Q3	0.232	0.040	0.423	
	Q4	0.304	0.102	0.505	
Ala	Q2	-0.158	-0.330	0.015	1.38E-02
	Q3	-0.140	-0.322	0.042	
	Q4	-0.297	-0.489	-0.105	
Sarcosine	Q2	0.031	-0.107	0.168	1.11E-01
	Q3	0.033	-0.113	0.179	
	Q4	0.058	-0.096	0.212	
N,N-Dimethylglycine	Q2	-0.002	-0.177	0.173	2.51E-01
	Q3	0.010	-0.175	0.195	
	Q4	0.033	-0.162	0.229	
Creatinine	Q2	-0.050	-0.218	0.118	5.22E-04
	Q3	-0.206	-0.384	-0.028	
	Q4	-0.367	-0.555	-0.179	
Ile	Q2	-0.001	-0.159	0.156	2.40E-01
	Q3	-0.115	-0.282	0.052	
	Q4	-0.095	-0.271	0.081	
Asp	Q2	-0.035	-0.165	0.094	1.02E-01
	Q3	0.127	-0.010	0.264	
	Q4	0.063	-0.081	0.208	
Carnitine	Q2	0.094	-0.076	0.264	8.79E-02
	Q3	0.108	-0.072	0.288	
	Q4	0.138	-0.052	0.328	
Uridine	Q2	-0.001	-0.103	0.101	3.76E-01
	Q3	0.045	-0.063	0.153	
	Q4	0.030	-0.084	0.144	
2-Oxoglutarate	Q2	0.151	-0.021	0.323	1.76E-02
	Q3	0.110	-0.073	0.293	
	Q4	0.289	0.097	0.482	

Women Variables

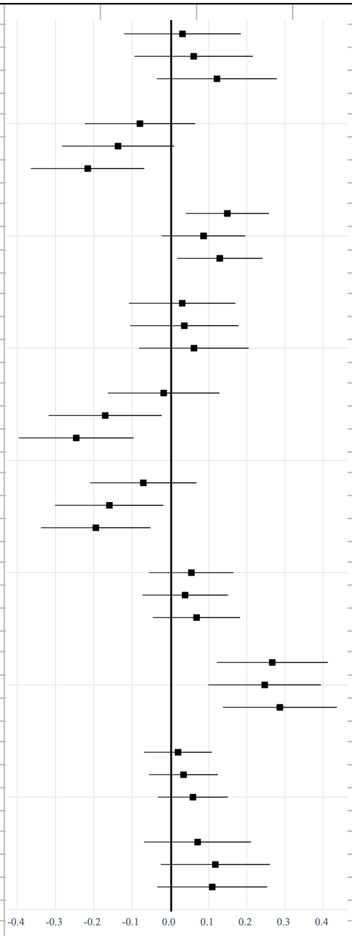

Metabolites	Cd Exposure	Standardized β	95%CI		trend FDR p
Trimethylamine N-oxide	Q2	0.032	-0.120	0.184	5.16E-02
	Q3	0.061	-0.093	0.216	
	Q4	0.122	-0.034	0.278	
Ala	Q2	-0.079	-0.223	0.065	5.65E-03
	Q3	-0.136	-0.282	0.010	
	Q4	-0.216	-0.364	-0.068	
Sarcosine	Q2	0.149	0.041	0.257	1.91E-02
	Q3	0.087	-0.022	0.196	
	Q4	0.130	0.019	0.241	
N,N-Dimethylglycine	Q2	0.031	-0.107	0.170	7.81E-03
	Q3	0.037	-0.104	0.178	
	Q4	0.062	-0.081	0.204	
Creatinine	Q2	-0.017	-0.163	0.128	2.33E-03
	Q3	-0.170	-0.318	-0.023	
	Q4	-0.246	-0.396	-0.097	
Ile	Q2	-0.071	-0.209	0.068	2.06E-03
	Q3	-0.159	-0.300	-0.018	
	Q4	-0.195	-0.337	-0.052	
Asp	Q2	0.055	-0.055	0.165	5.49E-04
	Q3	0.039	-0.073	0.151	
	Q4	0.069	-0.044	0.182	
Carnitine	Q2	0.267	0.123	0.411	4.99E-04
	Q3	0.247	0.100	0.394	
	Q4	0.287	0.138	0.435	
Uridine	Q2	0.020	-0.068	0.109	1.31E-04
	Q3	0.034	-0.055	0.124	
	Q4	0.059	-0.031	0.150	
2-Oxoglutarate	Q2	0.072	-0.068	0.211	6.72E-02
	Q3	0.118	-0.024	0.260	
	Q4	0.110	-0.033	0.253	

Multivariate regression model was adjusted by age (years), sex, systolic blood pressure (mmHg), smoke (pack-year), rice intake(bowl/week), BMI (kg/m2), HbA1c (%), LDLc (mg/dL), alcohol consumption (ethanol intake: g/day), physical activity (METs, quartiles), educational history (less than 10 years, 10 to 12 years, more than 12 years), dietary energy intake (kcal, quartiles), urinary Na/K ratio and uric acid (mg/dL).
P values were adjusted by FDR(False Discovery Rate) in each qurtile (94 penalties per analysis).
