# Supplementary material for: A population-based urinary and plasma metabolomics study of environmental exposure to cadmium
Source: Environ Health Prev Med. 2024 Mar 30;29:22. doi: 10.1265/ehpm.23-00218 (PMC10992994; doi:10.1265/ehpm.23-00218)
Supplement: Supplementary file 16 — Additional file 16: S Figure 16 Association between urine metabolites and cadmium exposure groups excluding participants of eGFR is >60 mL/min/1.73 m2. [file ehpm-29-022-s016.pdf]

S Figure 16 Association between urine metabolites and cadmium exposure groups excluding participants of eGFR is > 60 mL/min/1.73m2.

Forest plot in multivariate regression model (adjusted model)

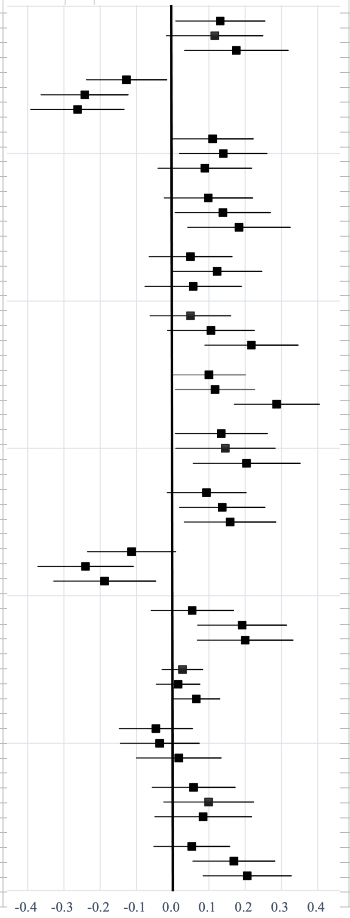

Metabolites	Cd Exposure	Standardized β	95%CI		trend FDR p
Trimethylamine N-oxide	Q2	0.131	0.008	0.254	1.32E-02
	Q3	0.116	-0.017	0.249	
	Q4	0.175	0.033	0.318	
Ala	Q2	-0.128	-0.239	-0.017	3.49E-04
	Q3	-0.243	-0.363	-0.123	
	Q4	-0.263	-0.392	-0.134	
Choline	Q2	0.110	-0.001	0.222	4.69E-02
	Q3	0.139	0.019	0.260	
	Q4	0.088	-0.041	0.218	
Taurine	Q2	0.098	-0.024	0.220	1.10E-02
	Q3	0.138	0.006	0.270	
	Q4	0.183	0.041	0.325	
1-Methylnicotinamide	Q2	0.049	-0.066	0.164	5.57E-02
	Q3	0.122	-0.002	0.246	
	Q4	0.056	-0.077	0.190	
ADMA	Q2	0.049	-0.062	0.160	4.88E-03
	Q3	0.106	-0.014	0.226	
	Q4	0.217	0.088	0.346	
Uridine	Q2	0.099	-0.002	0.201	4.04E-03
	Q3	0.117	0.007	0.227	
	Q4	0.287	0.169	0.405	
Adenosine	Q2	0.134	0.007	0.261	1.04E-02
	Q3	0.145	0.008	0.283	
	Q4	0.204	0.056	0.351	
Ethanolamine	Q2	0.093	-0.016	0.202	4.79E-02
	Q3	0.136	0.019	0.254	
	Q4	0.158	0.032	0.285	
Piperidine	Q2	-0.114	-0.235	0.008	1.40E-02
	Q3	-0.241	-0.372	-0.109	
	Q4	-0.188	-0.329	-0.047	
5-Aminolevulinate	Q2	0.054	-0.060	0.167	5.61E-04
	Q3	0.191	0.068	0.314	
	Q4	0.199	0.067	0.331	
Allantoin	Q2	0.027	-0.030	0.083	5.83E-02
	Q3	0.015	-0.046	0.075	
	Q4	0.065	-0.001	0.130	
7-Methylguanine	Q2	-0.047	-0.148	0.054	4.50E-03
	Q3	-0.036	-0.145	0.073	
	Q4	0.016	-0.101	0.134	
N1-Acetylspermidine	Q2	0.057	-0.057	0.172	1.67E-01
	Q3	0.099	-0.025	0.223	
	Q4	0.084	-0.049	0.217	
N8-Acetylspermidine	Q2	0.052	-0.052	0.157	4.81E-04
	Q3	0.169	0.056	0.282	
	Q4	0.205	0.084	0.327	

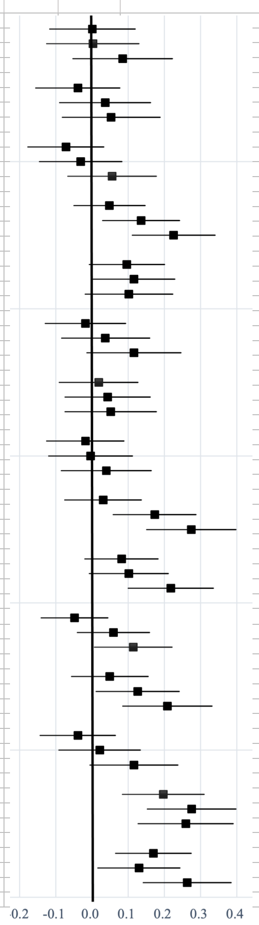

Metabolites	Cd Exposure	Standardized β	95%CI		trend FDR p
Gly-Leu	Q2	0.001	-0.118	0.119	5.46E-02
	Q3	0.002	-0.126	0.130	
	Q4	0.085	-0.053	0.222	
N-epsilon-Acetyllysine	Q2	-0.039	-0.156	0.077	4.00E-02
	Q3	0.036	-0.090	0.162	
	Q4	0.053	-0.083	0.188	
SAM+	Q2	-0.072	-0.178	0.033	5.27E-03
	Q3	-0.031	-0.145	0.083	
	Q4	0.055	-0.067	0.178	
1-Methyladenosine	Q2	0.048	-0.050	0.147	1.11E-03
	Q3	0.135	0.029	0.242	
	Q4	0.225	0.111	0.340	
Lactate	Q2	0.096	-0.008	0.201	2.30E-03
	Q3	0.116	0.003	0.229	
	Q4	0.102	-0.020	0.223	
Malonate	Q2	-0.018	-0.130	0.093	8.03E-10
	Q3	0.036	-0.085	0.157	
	Q4	0.116	-0.014	0.245	
4-Oxopentanoate	Q2	0.018	-0.091	0.127	7.40E-02
	Q3	0.043	-0.075	0.161	
	Q4	0.052	-0.075	0.178	
Succinate	Q2	-0.018	-0.126	0.089	6.29E-08
	Q3	-0.004	-0.120	0.112	
	Q4	0.039	-0.085	0.164	
Isethionate	Q2	0.030	-0.076	0.137	1.12E-16
	Q3	0.173	0.058	0.288	
	Q4	0.274	0.151	0.398	
5-Oxoproline	Q2	0.082	-0.020	0.183	2.43E-06
	Q3	0.102	-0.008	0.211	
	Q4	0.218	0.100	0.336	
Glutarate	Q2	-0.049	-0.141	0.044	3.74E-05
	Q3	0.059	-0.041	0.159	
	Q4	0.114	0.006	0.222	
Malate	Q2	0.049	-0.057	0.155	9.97E-18
	Q3	0.126	0.011	0.241	
	Q4	0.208	0.084	0.332	
Threonate	Q2	-0.039	-0.144	0.065	1.41E-11
	Q3	0.021	-0.092	0.134	
	Q4	0.116	-0.006	0.237	
Ethanolamine phosphate	Q2	0.197	0.084	0.310	1.71E-13
	Q3	0.275	0.153	0.397	
	Q4	0.259	0.127	0.391	
2-Oxoglutarate	Q2	0.170	0.065	0.275	9.85E-21
	Q3	0.130	0.016	0.243	
	Q4	0.263	0.141	0.385	

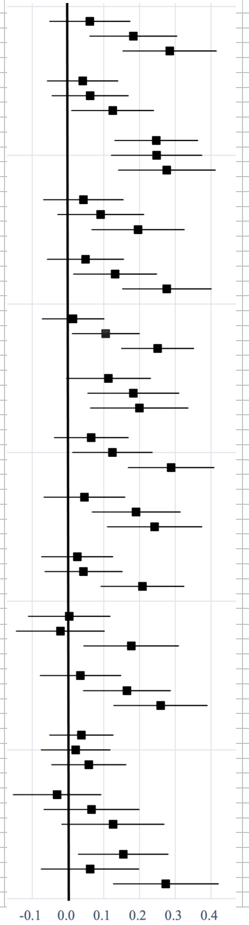

Metabolites	Cd Exposure	Standardized β	95%CI		trend FDR p
Pimelate	Q2	0.061	-0.051	0.173	3.75E-09
	Q3	0.183	0.061	0.304	
	Q4	0.285	0.154	0.416	
Urate	Q2	0.041	-0.058	0.140	4.45E-13
	Q3	0.062	-0.045	0.169	
	Q4	0.125	0.010	0.240	
Glycerophosphate	Q2	0.247	0.131	0.364	8.33E-10
	Q3	0.248	0.122	0.374	
	Q4	0.277	0.141	0.412	
trans-Aconitate	Q2	0.043	-0.067	0.154	3.60E-03
	Q3	0.092	-0.028	0.212	
	Q4	0.196	0.067	0.325	
cis-Aconitate	Q2	0.049	-0.058	0.156	1.35E-17
	Q3	0.132	0.017	0.248	
	Q4	0.277	0.153	0.401	
N-Acetylaspartate	Q2	0.015	-0.072	0.101	9.16E-34
	Q3	0.106	0.012	0.200	
	Q4	0.251	0.150	0.352	
Azelate	Q2	0.114	-0.004	0.231	3.35E-04
	Q3	0.183	0.056	0.310	
	Q4	0.200	0.063	0.336	
Isocitrate	Q2	0.066	-0.037	0.169	4.34E-25
	Q3	0.124	0.013	0.236	
	Q4	0.288	0.169	0.408	
Adipate	Q2	0.046	-0.067	0.160	2.30E-14
	Q3	0.191	0.068	0.314	
	Q4	0.243	0.111	0.375	
3-Hydroxy-3-methylglutarate	Q2	0.026	-0.074	0.126	1.02E-17
	Q3	0.044	-0.064	0.152	
	Q4	0.209	0.093	0.325	
2,3-Pyridinedicarboxylate	Q2	0.004	-0.110	0.118	3.28E-12
	Q3	-0.021	-0.145	0.102	
	Q4	0.177	0.045	0.310	
Suberate	Q2	0.035	-0.077	0.148	2.86E-07
	Q3	0.165	0.044	0.287	
	Q4	0.259	0.128	0.390	
Gluconate	Q2	0.038	-0.051	0.127	3.98E-12
	Q3	0.022	-0.074	0.118	
	Q4	0.059	-0.045	0.162	
Saccharate	Q2	-0.030	-0.153	0.093	2.86E-05
	Q3	0.066	-0.067	0.199	
	Q4	0.126	-0.017	0.269	
N-Acetylneuraminate	Q2	0.155	0.029	0.281	1.80E-10
	Q3	0.062	-0.074	0.198	
	Q4	0.274	0.128	0.421	

Multivariate regression model was adjusted by age (years),sex ,systolic blood pressure (mmHg), smoke (pack-year), rice intake(bowl/week), BMI (kg/m2), HbA1c (%), LDLc (mg/dL), alcohol consumption (ethanol intake: g/day), physical activity (METs, quartiles), educational history (less than 10 years, 10 to 12 years, more than 12 years), dietary energy intake (kcal, quartiles), urinary Na/K ratio and uric acid (mg/dL).
Trend p values were adjusted by FDR(False Discovery Rate) in each qurtile (123 penalties per analysis).
