# Supplementary material for: A population-based urinary and plasma metabolomics study of environmental exposure to cadmium
Source: Environ Health Prev Med. 2024 Mar 30;29:22. doi: 10.1265/ehpm.23-00218 (PMC10992994; doi:10.1265/ehpm.23-00218)
Supplement: Supplementary file 17 — Additional file 17: S Figure 17 Association between urine metabolites and cadmium exposure groups excluding participants of eGFR is >60 mL/min/1.73 m2 in men. [file ehpm-29-022-s017.pdf]

S Figure 17 Association between urine metabolites and cadmium exposure groups excluding participants of eGFR is > 60 mL/min/1.73m2 in men.

Forest plot in multivariate regression model (adjusted model)

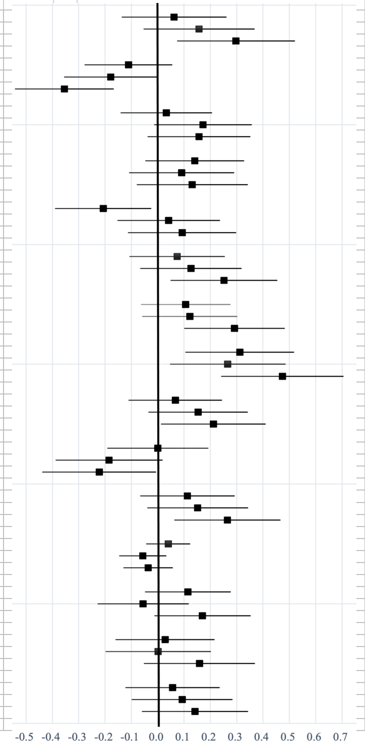

Variables					
Metabolites	Cd Exposure	Standardized β	95%CI		trend FDR p
	Q2	0.062	-0.135	0.260	
Trimethylamine N-oxide	Q3	0.157	-0.052	0.367	5.74E-03
	Q4	0.297	0.074	0.520	
	Q2	-0.111	-0.277	0.055	
Ala	Q3	-0.178	-0.354	-0.002	2.54E-04
	Q4	-0.355	-0.541	-0.168	
	Q2	0.033	-0.140	0.206	
Choline	Q3	0.172	-0.013	0.356	8.45E-02
	Q4	0.156	-0.038	0.351	
	Q2	0.141	-0.046	0.327	
Taurine	Q3	0.091	-0.107	0.289	2.36E-01
	Q4	0.131	-0.078	0.341	
	Q2	-0.207	-0.389	-0.025	
1-Methylnicotinamide	Q3	0.042	-0.152	0.236	2.46E-02
	Q4	0.093	-0.112	0.297	
	Q2	0.074	-0.106	0.253	
ADMA	Q3	0.126	-0.065	0.318	9.25E-03
	Q4	0.252	0.049	0.454	
	Q2	0.107	-0.062	0.275	
Uridine	Q3	0.122	-0.058	0.302	2.23E-02
	Q4	0.292	0.102	0.481	
	Q2	0.312	0.106	0.517	
Adenosine	Q3	0.266	0.047	0.484	5.33E-04
	Q4	0.473	0.242	0.704	
	Q2	0.067	-0.109	0.243	
Ethanolamine	Q3	0.153	-0.034	0.341	2.88E-02
	Q4	0.212	0.014	0.410	
	Q2	0.000	-0.191	0.191	
Piperidine	Q3	-0.185	-0.387	0.018	1.28E-02
	Q4	-0.222	-0.437	-0.007	
	Q2	0.113	-0.066	0.292	
5-Aminolevulinate	Q3	0.152	-0.039	0.342	1.77E-02
	Q4	0.264	0.064	0.465	
	Q2	0.040	-0.042	0.123	
Allantoin	Q3	-0.057	-0.145	0.031	1.74E-01
	Q4	-0.036	-0.129	0.057	
	Q2	0.115	-0.047	0.277	
7-Methylguanine	Q3	-0.055	-0.228	0.117	2.99E-02
	Q4	0.170	-0.012	0.352	
	Q2	0.028	-0.159	0.215	
N1-Acetylspermidine	Q3	0.002	-0.197	0.201	3.68E-01
	Q4	0.158	-0.052	0.368	
	Q2	0.057	-0.121	0.235	
N8-Acetylspermidine	Q3	0.093	-0.097	0.283	4.42E-01
	Q4	0.142	-0.059	0.342	

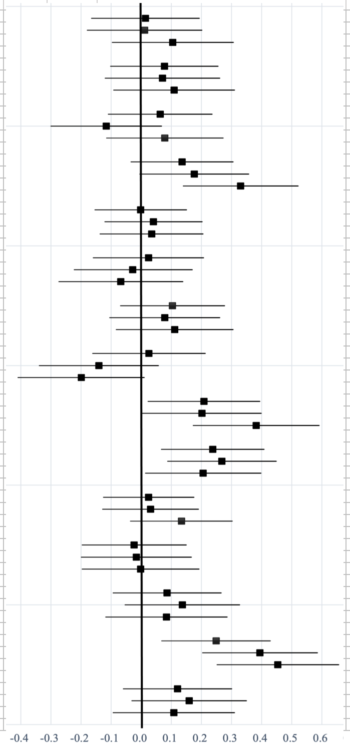

Variables					
Metabolites	Cd Exposure	Standardized β	95%CI		trend FDR p
	Q2	0.014	-0.165	0.193	
Gly-Leu	Q3	0.011	-0.179	0.202	2.54E-01
	Q4	0.105	-0.097	0.306	
	Q2	0.077	-0.102	0.256	
N-epsilon-Acetyllysine	Q3	0.070	-0.120	0.261	1.41E-01
	Q4	0.109	-0.092	0.310	
	Q2	0.062	-0.110	0.235	
SAM+	Q3	-0.117	-0.301	0.068	3.20E-01
	Q4	0.078	-0.116	0.272	
	Q2	0.135	-0.034	0.305	
1-Methyladenosine	Q3	0.176	-0.005	0.357	1.40E-03
	Q4	0.330	0.139	0.521	
	Q2	-0.002	-0.154	0.151	
Lactate	Q3	0.041	-0.121	0.202	2.39E-01
	Q4	0.034	-0.137	0.206	
	Q2	0.024	-0.160	0.208	
Malonate	Q3	-0.029	-0.223	0.165	5.08E-01
	Q4	-0.068	-0.274	0.138	
	Q2	0.104	-0.069	0.277	
4-Oxopentanoate	Q3	0.078	-0.105	0.261	2.36E-01
	Q4	0.111	-0.083	0.306	
	Q2	0.025	-0.162	0.212	
Succinate	Q3	-0.141	-0.339	0.057	4.97E-02
	Q4	-0.200	-0.410	0.010	
	Q2	0.208	0.022	0.395	
Isethionate	Q3	0.202	0.005	0.399	2.00E-03
	Q4	0.382	0.173	0.591	
	Q2	0.238	0.067	0.409	
5-Oxoproline	Q3	0.268	0.087	0.449	3.12E-02
	Q4	0.206	0.014	0.398	
	Q2	0.024	-0.126	0.175	
Glutarate	Q3	0.031	-0.128	0.190	1.24E-01
	Q4	0.133	-0.036	0.302	
	Q2	-0.023	-0.196	0.150	
Malate	Q3	-0.016	-0.200	0.167	8.77E-01
	Q4	-0.002	-0.196	0.192	
	Q2	0.086	-0.094	0.266	
Threonate	Q3	0.136	-0.054	0.327	2.11E-01
	Q4	0.084	-0.118	0.286	
	Q2	0.248	0.068	0.429	
Ethanolamine phosphate	Q3	0.394	0.203	0.585	6.40E-06
	Q4	0.454	0.251	0.657	
	Q2	0.120	-0.060	0.300	
2-Oxoglutarate	Q3	0.159	-0.032	0.350	4.80E-01
	Q4	0.108	-0.094	0.311	

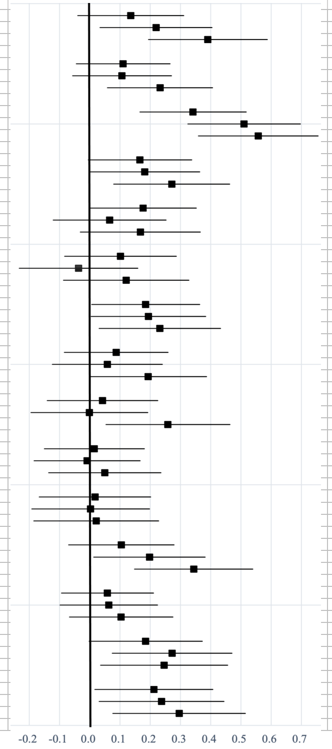

Variables					
Metabolites	Cd Exposure	Standardized β	95%CI		trend FDR p
	Q2	0.136	-0.040	0.311	
Pimelate	Q3	0.219	0.034	0.405	9.82E-05
	Q4	0.391	0.194	0.588	
	Q2	0.111	-0.044	0.266	
Urate	Q3	0.107	-0.057	0.271	1.68E-03
	Q4	0.233	0.059	0.407	
	Q2	0.342	0.166	0.518	
Glycerophosphate	Q3	0.511	0.324	0.698	6.18E-08
	Q4	0.558	0.360	0.756	
	Q2	0.166	-0.005	0.337	
trans-Aconitate	Q3	0.183	0.001	0.364	4.30E-02
	Q4	0.271	0.079	0.464	
	Q2	0.177	0.000	0.353	
cis-Aconitate	Q3	0.066	-0.121	0.253	4.78E-01
	Q4	0.168	-0.031	0.366	
	Q2	0.102	-0.083	0.287	
N-Acetylaspartate	Q3	-0.037	-0.233	0.159	6.72E-01
	Q4	0.120	-0.087	0.328	
	Q2	0.185	0.006	0.364	
Azelate	Q3	0.194	0.005	0.384	3.86E-02
	Q4	0.232	0.031	0.433	
	Q2	0.088	-0.084	0.260	
Isocitrate	Q3	0.058	-0.123	0.240	1.07E-01
	Q4	0.194	0.001	0.387	
	Q2	0.043	-0.140	0.225	
Adipate	Q3	-0.001	-0.194	0.193	2.80E-02
	Q4	0.259	0.054	0.464	
	Q2	0.015	-0.150	0.181	
3-Hydroxy-3-methylglutarate	Q3	-0.008	-0.184	0.167	9.22E-01
	Q4	0.050	-0.136	0.236	
	Q2	0.018	-0.167	0.202	
2,3-Pyridinedicarboxylate	Q3	0.003	-0.192	0.198	9.21E-01
	Q4	0.022	-0.185	0.229	
	Q2	0.105	-0.070	0.279	
Suberate	Q3	0.198	0.013	0.383	3.09E-04
	Q4	0.344	0.148	0.540	
	Q2	0.059	-0.094	0.211	
Gluconate	Q3	0.063	-0.099	0.224	1.36E-01
	Q4	0.104	-0.067	0.276	
	Q2	0.185	-0.002	0.373	
Saccharate	Q3	0.273	0.074	0.471	1.87E-02
	Q4	0.247	0.036	0.457	
	Q2	0.212	0.017	0.408	
N-Acetylneuraminate	Q3	0.238	0.031	0.445	2.01E-02
	Q4	0.296	0.076	0.516	

Multivariate regression model was adjusted by age (years),sex ,systolic blood pressure (mmHg), smoke (pack-year), rice intake(bowl/week), BMI (kg/m2), HbA1c (%), LDLc (mg/dL), alcohol consumption (ethanol intake: g/day), physical activity (METs, quartiles), educational history (less than 10 years, 10 to 12 years, more than 12 years), dietary energy intake (kcal, quartiles), urinary Na/K ratio and uric acid (mg/dL).
Trend p values were adjusted by FDR(False Discovery Rate) in each qurtile (123 penalties per analysis).
