# Supplementary material for: A population-based urinary and plasma metabolomics study of environmental exposure to cadmium
Source: Environ Health Prev Med. 2024 Mar 30;29:22. doi: 10.1265/ehpm.23-00218 (PMC10992994; doi:10.1265/ehpm.23-00218)
Supplement: Supplementary file 18 — Additional file 18: S Figure 18 Association between urine metabolites and cadmium exposure groups excluding participants of eGFR is >60 mL/min/1.73 m2 in women. [file ehpm-29-022-s018.pdf]

S Figure 18 Association between urine metabolites and cadmium exposure groups excluding participants of eGFR is > 60 mL/min/1.73m2 in women.

Forest plot in multivariate regression model (adjusted model)

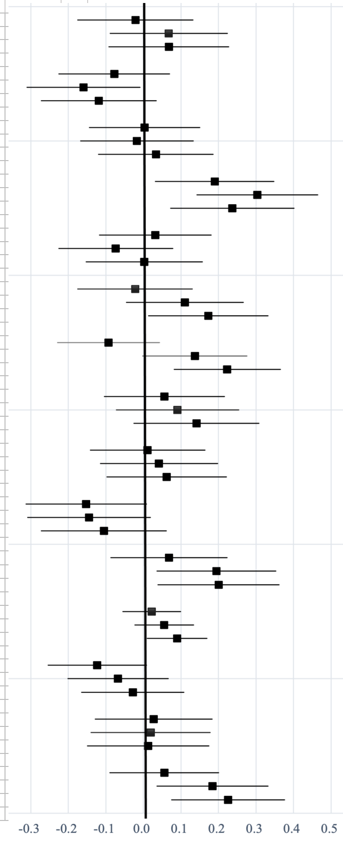

| Metabolites | Cd Exposure | Standardized β | 95%CI | | trend FDR p |
|---|---|---|---|---|---|
| Trimethylamine N-oxide | Q2 | -0.021 | -0.175 | 0.132 | 1.46E-01 |
| | Q3 | 0.068 | -0.089 | 0.224 | |
| | Q4 | 0.068 | -0.092 | 0.228 | |
| Ala | Q2 | -0.078 | -0.225 | 0.070 | 1.89E-01 |
| | Q3 | -0.160 | -0.310 | -0.009 | |
| | Q4 | -0.119 | -0.272 | 0.035 | |
| Choline | Q2 | 0.003 | -0.144 | 0.150 | 3.77E-01 |
| | Q3 | -0.018 | -0.168 | 0.133 | |
| | Q4 | 0.033 | -0.120 | 0.186 | |
| Taurine | Q2 | 0.190 | 0.032 | 0.348 | 6.16E-03 |
| | Q3 | 0.304 | 0.143 | 0.465 | |
| | Q4 | 0.237 | 0.072 | 0.401 | |
| 1-Methylnicotinamide | Q2 | 0.032 | -0.117 | 0.181 | 9.75E-01 |
| | Q3 | -0.074 | -0.226 | 0.078 | |
| | Q4 | 0.002 | -0.152 | 0.157 | |
| ADMA | Q2 | -0.022 | -0.175 | 0.131 | 4.43E-02 |
| | Q3 | 0.111 | -0.045 | 0.267 | |
| | Q4 | 0.173 | 0.014 | 0.332 | |
| Uridine | Q2 | -0.093 | -0.229 | 0.043 | 6.81E-03 |
| | Q3 | 0.137 | -0.002 | 0.276 | |
| | Q4 | 0.223 | 0.082 | 0.365 | |
| Adenosine | Q2 | 0.056 | -0.104 | 0.216 | 8.77E-02 |
| | Q3 | 0.091 | -0.073 | 0.254 | |
| | Q4 | 0.141 | -0.025 | 0.308 | |
| Ethanolamine | Q2 | 0.011 | -0.142 | 0.164 | 8.08E-01 |
| | Q3 | 0.041 | -0.115 | 0.198 | |
| | Q4 | 0.062 | -0.097 | 0.221 | |
| Piperidine | Q2 | -0.152 | -0.313 | 0.008 | 4.19E-01 |
| | Q3 | -0.145 | -0.309 | 0.019 | |
| | Q4 | -0.105 | -0.272 | 0.061 | |
| 5-Aminolevulinate | Q2 | 0.068 | -0.087 | 0.223 | 1.69E-03 |
| | Q3 | 0.195 | 0.036 | 0.353 | |
| | Q4 | 0.200 | 0.039 | 0.362 | |
| Allantoin | Q2 | 0.022 | -0.055 | 0.099 | 9.62E-02 |
| | Q3 | 0.055 | -0.023 | 0.134 | |
| | Q4 | 0.090 | 0.010 | 0.169 | |
| 7-Methylguanine | Q2 | -0.123 | -0.254 | 0.008 | 1.12E-01 |
| | Q3 | -0.068 | -0.201 | 0.066 | |
| | Q4 | -0.028 | -0.165 | 0.108 | |
| N1-Acetylspermidine | Q2 | 0.027 | -0.128 | 0.183 | 7.92E-01 |
| | Q3 | 0.019 | -0.140 | 0.178 | |
| | Q4 | 0.012 | -0.149 | 0.174 | |
| N8-Acetylspermidine | Q2 | 0.056 | -0.089 | 0.201 | 2.79E-04 |
| | Q3 | 0.184 | 0.036 | 0.332 | |
| | Q4 | 0.225 | 0.075 | 0.376 | |

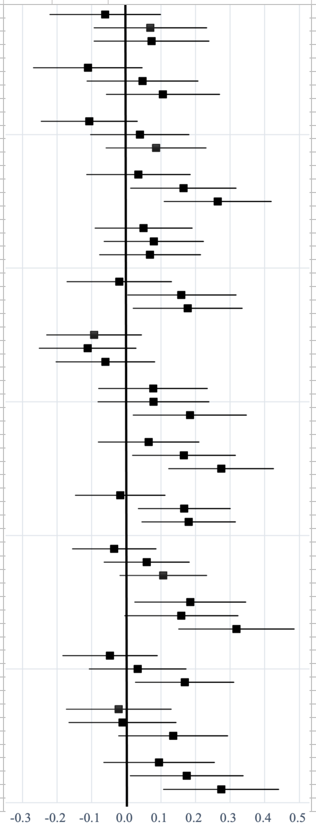

| Metabolites | Cd Exposure | Standardized β | 95%CI | | trend FDR p |
|---|---|---|---|---|---|
| Gly-Leu | Q2 | -0.061 | -0.220 | 0.099 | 9.26E-02 |
| | Q3 | 0.070 | -0.093 | 0.232 | |
| | Q4 | 0.073 | -0.093 | 0.239 | |
| N-epsilon-Acetyllysine | Q2 | -0.111 | -0.268 | 0.046 | 1.76E-02 |
| | Q3 | 0.047 | -0.113 | 0.208 | |
| | Q4 | 0.106 | -0.058 | 0.269 | |
| SAM+ | Q2 | -0.107 | -0.246 | 0.032 | 1.16E-02 |
| | Q3 | 0.039 | -0.103 | 0.181 | |
| | Q4 | 0.086 | -0.058 | 0.230 | |
| 1-Methyladenosine | Q2 | 0.035 | -0.114 | 0.185 | 7.71E-03 |
| | Q3 | 0.165 | 0.013 | 0.317 | |
| | Q4 | 0.264 | 0.109 | 0.419 | |
| Lactate | Q2 | 0.050 | -0.090 | 0.191 | 5.56E-01 |
| | Q3 | 0.080 | -0.064 | 0.223 | |
| | Q4 | 0.069 | -0.077 | 0.215 | |
| Malonate | Q2 | -0.020 | -0.171 | 0.131 | 1.15E-03 |
| | Q3 | 0.159 | 0.004 | 0.314 | |
| | Q4 | 0.177 | 0.020 | 0.335 | |
| 4-Oxopentanoate | Q2 | -0.093 | -0.230 | 0.044 | 9.08E-01 |
| | Q3 | -0.112 | -0.252 | 0.029 | |
| | Q4 | -0.060 | -0.203 | 0.083 | |
| Succinate | Q2 | 0.078 | -0.079 | 0.235 | 3.11E-03 |
| | Q3 | 0.079 | -0.082 | 0.239 | |
| | Q4 | 0.184 | 0.020 | 0.347 | |
| Isethionate | Q2 | 0.065 | -0.081 | 0.210 | 4.20E-03 |
| | Q3 | 0.167 | 0.018 | 0.315 | |
| | Q4 | 0.274 | 0.123 | 0.426 | |
| 5-Oxoproline | Q2 | -0.017 | -0.147 | 0.112 | 1.37E-01 |
| | Q3 | 0.168 | 0.035 | 0.300 | |
| | Q4 | 0.180 | 0.045 | 0.315 | |
| Glutarate | Q2 | -0.035 | -0.155 | 0.086 | 2.34E-01 |
| | Q3 | 0.059 | -0.064 | 0.182 | |
| | Q4 | 0.107 | -0.019 | 0.232 | |
| Malate | Q2 | 0.185 | 0.025 | 0.345 | 3.80E-04 |
| | Q3 | 0.159 | -0.004 | 0.323 | |
| | Q4 | 0.318 | 0.152 | 0.485 | |
| Threonate | Q2 | -0.047 | -0.184 | 0.090 | 4.15E-03 |
| | Q3 | 0.033 | -0.107 | 0.173 | |
| | Q4 | 0.169 | 0.027 | 0.311 | |
| Ethanolamine phosphate | Q2 | -0.022 | -0.173 | 0.130 | 3.17E-02 |
| | Q3 | -0.011 | -0.166 | 0.144 | |
| | Q4 | 0.136 | -0.022 | 0.294 | |
| 2-Oxoglutarate | Q2 | 0.095 | -0.065 | 0.255 | 7.99E-04 |
| | Q3 | 0.174 | 0.011 | 0.338 | |
| | Q4 | 0.274 | 0.108 | 0.440 | |

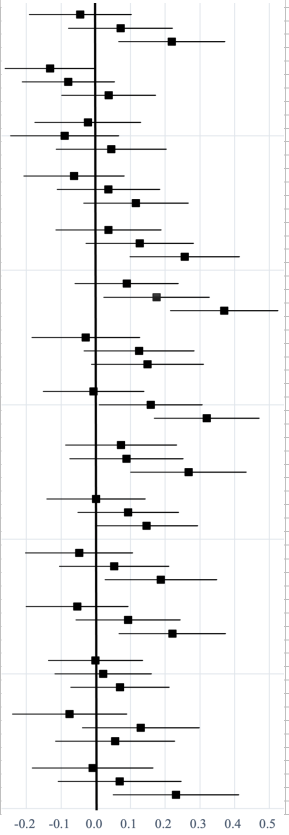

| Metabolites | Cd Exposure | Standardized β | 95%CI | | trend FDR p |
|---|---|---|---|---|---|
| Pimelate | Q2 | -0.044 | -0.191 | 0.102 | 1.67E-03 |
| | Q3 | 0.071 | -0.078 | 0.221 | |
| | Q4 | 0.219 | 0.066 | 0.371 | |
| Urate | Q2 | -0.132 | -0.261 | -0.002 | 2.22E-03 |
| | Q3 | -0.079 | -0.212 | 0.054 | |
| | Q4 | 0.037 | -0.098 | 0.172 | |
| Glycerophosphate | Q2 | -0.023 | -0.175 | 0.130 | 4.34E-01 |
| | Q3 | -0.090 | -0.246 | 0.066 | |
| | Q4 | 0.045 | -0.114 | 0.203 | |
| trans-Aconitate | Q2 | -0.062 | -0.207 | 0.082 | 1.64E-01 |
| | Q3 | 0.036 | -0.111 | 0.184 | |
| | Q4 | 0.116 | -0.035 | 0.266 | |
| cis-Aconitate | Q2 | 0.037 | -0.115 | 0.188 | 3.74E-03 |
| | Q3 | 0.127 | -0.028 | 0.282 | |
| | Q4 | 0.256 | 0.098 | 0.414 | |
| N-Acetylaspartate | Q2 | 0.089 | -0.059 | 0.238 | 2.11E-06 |
| | Q3 | 0.175 | 0.023 | 0.327 | |
| | Q4 | 0.370 | 0.216 | 0.525 | |
| Azelate | Q2 | -0.029 | -0.184 | 0.126 | 3.59E-02 |
| | Q3 | 0.125 | -0.033 | 0.283 | |
| | Q4 | 0.149 | -0.012 | 0.310 | |
| Isocitrate | Q2 | -0.006 | -0.151 | 0.139 | 4.03E-06 |
| | Q3 | 0.158 | 0.010 | 0.306 | |
| | Q4 | 0.319 | 0.168 | 0.470 | |
| Adipate | Q2 | 0.073 | -0.087 | 0.233 | 4.22E-03 |
| | Q3 | 0.089 | -0.074 | 0.252 | |
| | Q4 | 0.267 | 0.101 | 0.434 | |
| 3-Hydroxy-3-methylglutarate | Q2 | 0.001 | -0.141 | 0.143 | 4.07E-03 |
| | Q3 | 0.093 | -0.051 | 0.238 | |
| | Q4 | 0.146 | -0.001 | 0.294 | |
| 2,3-Pyridinedicarboxylate | Q2 | -0.048 | -0.202 | 0.107 | 6.35E-03 |
| | Q3 | 0.053 | -0.105 | 0.211 | |
| | Q4 | 0.188 | 0.027 | 0.348 | |
| Suberate | Q2 | -0.054 | -0.201 | 0.093 | 2.45E-03 |
| | Q3 | 0.093 | -0.057 | 0.243 | |
| | Q4 | 0.220 | 0.067 | 0.373 | |
| Gluconate | Q2 | -0.001 | -0.137 | 0.135 | 1.84E-01 |
| | Q3 | 0.021 | -0.118 | 0.160 | |
| | Q4 | 0.070 | -0.072 | 0.211 | |
| Saccharate | Q2 | -0.075 | -0.240 | 0.090 | 2.37E-01 |
| | Q3 | 0.130 | -0.039 | 0.298 | |
| | Q4 | 0.056 | -0.116 | 0.227 | |
| N-Acetylneuraminate | Q2 | -0.009 | -0.183 | 0.165 | 1.00E-02 |
| | Q3 | 0.069 | -0.109 | 0.246 | |
| | Q4 | 0.231 | 0.050 | 0.412 | |

Multivariate regression model was adjusted by age (years),sex ,systolic blood pressure (mmHg), smoke (pack-year), rice intake(bowl/week), BMI (kg/m2), HbA1c (%), LDLc (mg/dL), alcohol consumption (ethanol intake: g/day), physical activity (METs, quartiles), educational history (less than 10 years, 10 to 12 years, more than 12 years), dietary energy intake (kcal, quartiles), urinary Na/K ratio and uric acid (mg/dL).
Trend p values were adjusted by FDR(False Discovery Rate) in each qurtile (123 penalties per analysis).
