# Supplementary material for: A population-based urinary and plasma metabolomics study of environmental exposure to cadmium
Source: Environ Health Prev Med. 2024 Mar 30;29:22. doi: 10.1265/ehpm.23-00218 (PMC10992994; doi:10.1265/ehpm.23-00218)
Supplement: Supplementary file 20 — Additional file 20: S Figure 20 Association between urine metabolites and cadmium exposure groups excluding participants of eGFR is >60 mL/min/1.73 m2 in sex stratification. [file ehpm-29-022-s020.pdf]

S Figure 20 Association between plasma metabolites and cadmium exposure groups excluding participants of eGFR is > 60 mL/min/1.73m2 in sex stratification.

Men Variables

Forest plot in univariate regression model (adjusted model)

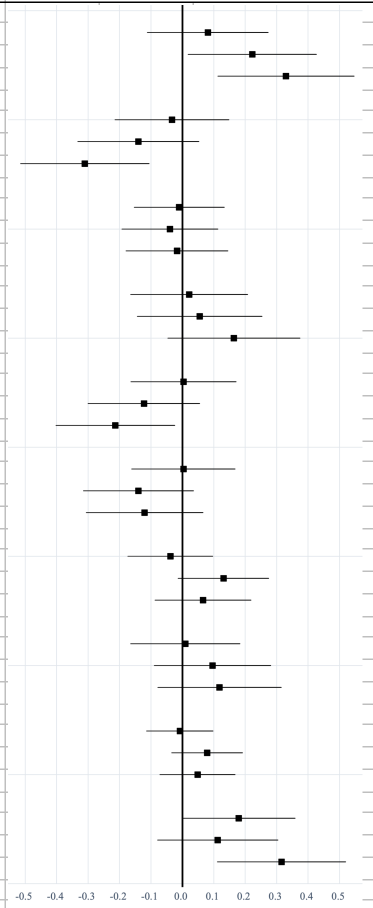

Metabolites	Cd Exposure	Standardized β	95%CI		trend FDR p
	Q2	0.081	-0.111	0.273	
Trimethylamine N-oxide	Q3	0.222	0.018	0.426	3.98E-04
	Q4	0.330	0.113	0.547	
	Q2	-0.033	-0.214	0.148	
Ala	Q3	-0.140	-0.333	0.053	9.60E-03
	Q4	-0.311	-0.515	-0.106	
	Q2	-0.010	-0.153	0.133	
Sarcosine	Q3	-0.040	-0.192	0.113	1.56E-01
	Q4	-0.017	-0.179	0.145	
	Q2	0.022	-0.165	0.208	
N,N-Dimethylglycine	Q3	0.055	-0.143	0.253	3.57E-02
	Q4	0.164	-0.046	0.375	
	Q2	0.004	-0.164	0.171	
Creatinine	Q3	-0.122	-0.300	0.055	8.26E-02
	Q4	-0.213	-0.402	-0.024	
	Q2	0.003	-0.161	0.168	
Ile	Q3	-0.140	-0.315	0.035	8.47E-02
	Q4	-0.120	-0.306	0.066	
	Q2	-0.038	-0.173	0.097	
Asp	Q3	0.131	-0.013	0.275	6.20E-02
	Q4	0.066	-0.087	0.219	
	Q2	0.010	-0.165	0.184	
Carnitine	Q3	0.096	-0.089	0.281	1.20E-01
	Q4	0.118	-0.078	0.315	
	Q2	-0.008	-0.114	0.098	
Uridine	Q3	0.079	-0.034	0.191	6.84E-02
	Q4	0.048	-0.071	0.168	
	Q2	0.179	-0.001	0.359	
2-Oxoglutarate	Q3	0.112	-0.079	0.304	9.56E-03
	Q4	0.315	0.112	0.519	

Women Variables

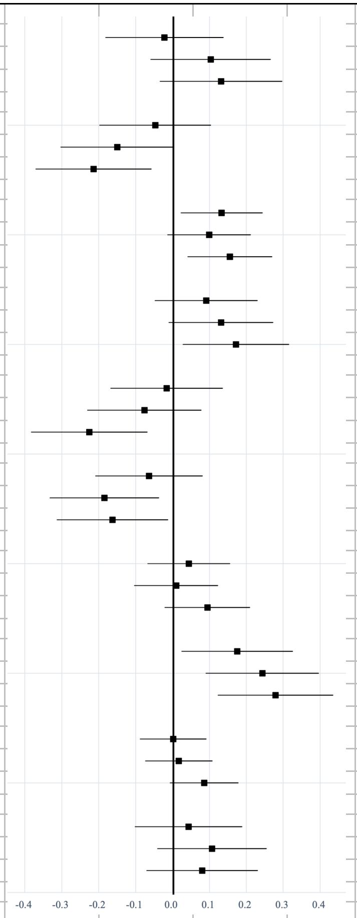

Metabolites	Cd Exposure	Standardized β	95%CI		trend FDR p
	Q2	-0.022	-0.181	0.137	
Trimethylamine N-oxide	Q3	0.103	-0.059	0.265	2.32E-02
	Q4	0.132	-0.033	0.297	
	Q2	-0.047	-0.198	0.104	
Ala	Q3	-0.149	-0.303	0.004	7.88E-03
	Q4	-0.214	-0.370	-0.058	
	Q2	0.133	0.023	0.244	
Sarcosine	Q3	0.099	-0.013	0.211	4.66E-03
	Q4	0.156	0.041	0.270	
	Q2	0.091	-0.047	0.230	
N,N-Dimethylglycine	Q3	0.131	-0.009	0.272	8.72E-05
	Q4	0.172	0.029	0.315	
	Q2	-0.016	-0.168	0.135	
Creatinine	Q3	-0.076	-0.230	0.078	3.33E-02
	Q4	-0.226	-0.382	-0.069	
	Q2	-0.064	-0.209	0.081	
Ile	Q3	-0.185	-0.332	-0.037	1.66E-02
	Q4	-0.163	-0.313	-0.012	
	Q2	0.044	-0.067	0.155	
Asp	Q3	0.010	-0.103	0.123	4.01E-04
	Q4	0.094	-0.021	0.210	
	Q2	0.175	0.025	0.325	
Carnitine	Q3	0.243	0.091	0.396	1.92E-04
	Q4	0.279	0.123	0.435	
	Q2	0.002	-0.087	0.091	
Uridine	Q3	0.017	-0.073	0.108	5.67E-05
	Q4	0.086	-0.006	0.178	
	Q2	0.044	-0.101	0.188	
2-Oxoglutarate	Q3	0.107	-0.040	0.255	9.79E-02
	Q4	0.080	-0.070	0.230	

Multivariate regression model was adjusted by age (years), sex, systolic blood pressure (mmHg), smoke (pack-year), rice intake(bowl/week), BMI (kg/m2), HbA1c (%), LDLc (mg/dL), alcohol consumption (ethanol intake: g/day), physical activity (METs, quartiles), educational history (less than 10 years, 10 to 12 years, more than 12 years), dietary energy intake (kcal, quartiles), urinary Na/K ratio and uric acid (mg/dL).

P values were adjusted by FDR(False Discovery Rate) in each qurtile (94 penalties per analysis).
