# Supplementary material for: A population-based urinary and plasma metabolomics study of environmental exposure to cadmium
Source: Environ Health Prev Med. 2024 Mar 30;29:22. doi: 10.1265/ehpm.23-00218 (PMC10992994; doi:10.1265/ehpm.23-00218)
Supplement: Supplementary file 21 — Additional file 21: S Figure 21 Association between urine metabolites and cadmium exposure groups excluding participants of HbA1c is <6.5% or prescribing diabetes medication. [file ehpm-29-022-s021.pdf]

S Figure 21 Association between urine metabolites and cadmium exposure groups excluding participants of HbA1c is < 6.5% or prescribing diabetes medication.

Forest plot in multivariate regression model (adjusted model)

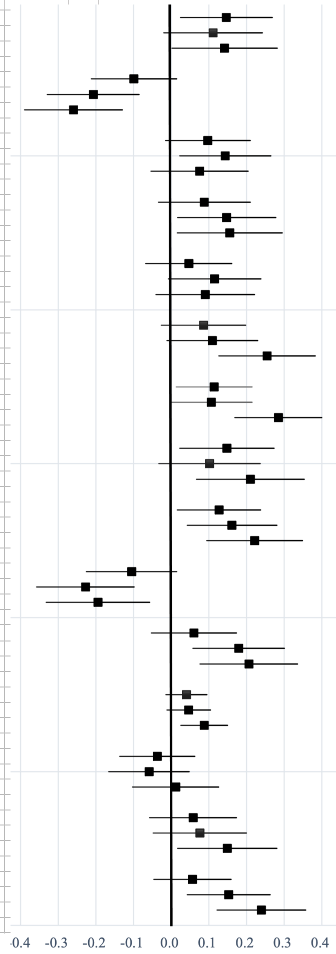

Metabolites	Cd Exposure	Standardized β	95%CI		trend FDR p
	Q2	0.146	0.024	0.268	
Trimethylamine N-oxide	Q3	0.111	-0.021	0.242	3.50E-02
	Q4	0.141	0.002	0.281	
	Q2	-0.099	-0.213	0.015	
Ala	Q3	-0.207	-0.329	-0.085	1.41E-03
	Q4	-0.260	-0.390	-0.130	
	Q2	0.097	-0.016	0.210	
Choline	Q3	0.143	0.022	0.264	3.35E-02
	Q4	0.075	-0.055	0.204	
	Q2	0.088	-0.034	0.210	
Taurine	Q3	0.147	0.016	0.278	1.22E-02
	Q4	0.155	0.016	0.295	
	Q2	0.047	-0.068	0.161	
1-Methylnicotinamide	Q3	0.115	-0.008	0.238	9.86E-03
	Q4	0.090	-0.041	0.221	
	Q2	0.086	-0.027	0.198	
ADMA	Q3	0.109	-0.011	0.230	1.16E-03
	Q4	0.254	0.126	0.383	
	Q2	0.114	0.013	0.215	
Uridine	Q3	0.106	-0.002	0.215	4.76E-03
	Q4	0.284	0.169	0.400	
	Q2	0.147	0.022	0.273	
Adenosine	Q3	0.102	-0.033	0.237	1.25E-02
	Q4	0.210	0.067	0.354	
	Q2	0.127	0.016	0.238	
Ethanolamine	Q3	0.161	0.042	0.280	3.29E-03
	Q4	0.222	0.094	0.349	
	Q2	-0.105	-0.226	0.015	
Piperidine	Q3	-0.228	-0.358	-0.099	1.25E-02
	Q4	-0.195	-0.332	-0.057	
	Q2	0.060	-0.054	0.173	
5-Aminolevulinate	Q3	0.179	0.057	0.301	1.57E-04
	Q4	0.206	0.076	0.336	
	Q2	0.040	-0.014	0.095	
Allantoin	Q3	0.046	-0.012	0.105	3.02E-02
	Q4	0.087	0.025	0.149	
	Q2	-0.037	-0.137	0.063	
7-Methylguanine	Q3	-0.059	-0.167	0.048	4.47E-03
	Q4	0.012	-0.103	0.126	
	Q2	0.058	-0.058	0.173	
N1-Acetylspermidine	Q3	0.076	-0.048	0.200	1.40E-02
	Q4	0.149	0.016	0.281	
	Q2	0.056	-0.047	0.159	
N8-Acetylspermidine	Q3	0.152	0.042	0.263	1.79E-05
	Q4	0.239	0.121	0.357	

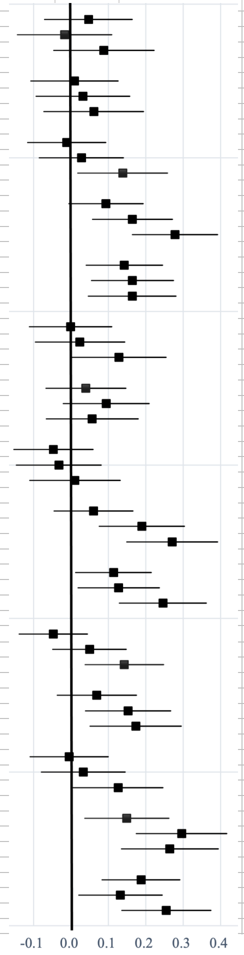

Metabolites	Cd Exposure	Standardized β	95%CI		trend FDR p
	Q2	0.046	-0.071	0.164	
Gly-Leu	Q3	-0.017	-0.143	0.109	4.57E-02
	Q4	0.088	-0.047	0.222	
	Q2	0.009	-0.108	0.126	
N-epsilon-Acetyllysine	Q3	0.032	-0.094	0.157	3.16E-02
	Q4	0.061	-0.073	0.194	
	Q2	-0.012	-0.117	0.093	
SAM+	Q3	0.028	-0.085	0.141	9.56E-05
	Q4	0.138	0.018	0.259	
	Q2	0.093	-0.006	0.193	
1-Methyladenosine	Q3	0.164	0.057	0.271	6.24E-05
	Q4	0.278	0.164	0.392	
	Q2	0.143	0.040	0.245	
Lactate	Q3	0.164	0.054	0.274	2.11E-02
	Q4	0.164	0.046	0.281	
	Q2	-0.001	-0.111	0.109	
Malonate	Q3	0.023	-0.096	0.141	4.96E-03
	Q4	0.128	0.001	0.255	
	Q2	0.040	-0.068	0.147	
4-Oxopentanoate	Q3	0.094	-0.021	0.209	2.53E-02
	Q4	0.057	-0.067	0.180	
	Q2	-0.047	-0.153	0.059	
Succinate	Q3	-0.033	-0.147	0.081	1.38E-01
	Q4	0.010	-0.111	0.132	
	Q2	0.060	-0.046	0.166	
Isethionate	Q3	0.189	0.075	0.303	3.46E-05
	Q4	0.270	0.148	0.392	
	Q2	0.113	0.012	0.215	
5-Oxoproline	Q3	0.127	0.018	0.236	2.70E-03
	Q4	0.246	0.129	0.362	
	Q2	-0.048	-0.139	0.044	
Glutarate	Q3	0.049	-0.049	0.148	6.64E-04
	Q4	0.142	0.037	0.248	
	Q2	0.069	-0.038	0.175	
Malate	Q3	0.153	0.039	0.267	2.74E-03
	Q4	0.173	0.051	0.295	
	Q2	-0.005	-0.110	0.099	
Threonate	Q3	0.033	-0.080	0.145	4.09E-03
	Q4	0.126	0.006	0.246	
	Q2	0.149	0.036	0.262	
Ethanolamine phosphate	Q3	0.296	0.175	0.417	1.05E-06
	Q4	0.264	0.135	0.394	
	Q2	0.187	0.083	0.291	
2-Oxoglutarate	Q3	0.132	0.020	0.244	2.45E-04
	Q4	0.255	0.135	0.374	

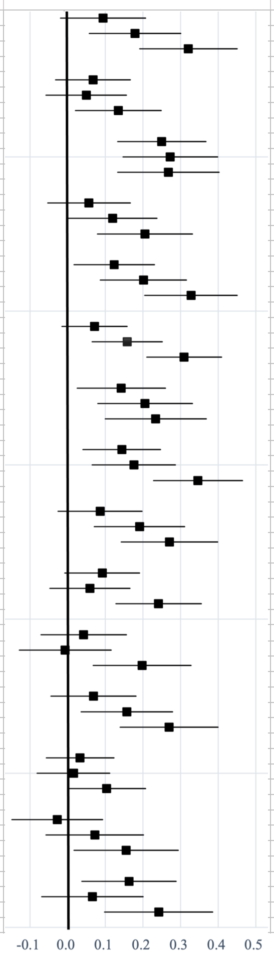

Metabolites	Cd Exposure	Standardized β	95%CI		trend FDR p
	Q2	0.094	-0.019	0.207	
Pimelate	Q3	0.179	0.058	0.301	6.70E-07
	Q4	0.321	0.191	0.451	
	Q2	0.068	-0.032	0.167	
Urate	Q3	0.050	-0.057	0.157	4.48E-05
	Q4	0.135	0.020	0.249	
	Q2	0.251	0.133	0.368	
Glycerophosphate	Q3	0.273	0.147	0.400	1.06E-04
	Q4	0.268	0.133	0.403	
	Q2	0.057	-0.053	0.167	
trans-Aconitate	Q3	0.120	0.002	0.238	2.76E-03
	Q4	0.206	0.079	0.332	
	Q2	0.124	0.017	0.231	
cis-Aconitate	Q3	0.202	0.087	0.317	8.42E-07
	Q4	0.328	0.205	0.451	
	Q2	0.072	-0.015	0.158	
N-Acetylaspartate	Q3	0.159	0.066	0.252	1.97E-10
	Q4	0.310	0.210	0.409	
	Q2	0.143	0.025	0.260	
Azelate	Q3	0.206	0.080	0.332	9.60E-04
	Q4	0.234	0.100	0.369	
	Q2	0.144	0.041	0.247	
Isocitrate	Q3	0.176	0.065	0.287	2.18E-09
	Q4	0.347	0.228	0.465	
	Q2	0.087	-0.025	0.199	
Adipate	Q3	0.191	0.071	0.312	1.34E-05
	Q4	0.271	0.143	0.399	
	Q2	0.092	-0.007	0.191	
3-Hydroxy-3-methylglutarate	Q3	0.060	-0.047	0.166	3.13E-05
	Q4	0.242	0.128	0.356	
	Q2	0.043	-0.071	0.156	
2,3-Pyridinedicarboxylate	Q3	-0.006	-0.129	0.116	5.80E-03
	Q4	0.198	0.068	0.329	
	Q2	0.069	-0.044	0.182	
Suberate	Q3	0.158	0.036	0.280	2.33E-05
	Q4	0.270	0.140	0.400	
	Q2	0.033	-0.057	0.123	
Gluconate	Q3	0.016	-0.081	0.113	2.44E-03
	Q4	0.104	0.001	0.207	
	Q2	-0.027	-0.148	0.093	
Saccharate	Q3	0.073	-0.057	0.202	7.23E-03
	Q4	0.156	0.017	0.294	
	Q2	0.163	0.038	0.289	
N-Acetylneuraminate	Q3	0.066	-0.068	0.201	8.42E-03
	Q4	0.242	0.098	0.386	

Multivariate regression model was adjusted by age (years),sex ,systolic blood pressure (mmHg), smoke (pack-year), rice intake(bowl/week), BMI (kg/m2), HbA1c (%), LDLc (mg/dL), alcohol consumption (ethanol intake: g/day), physical activity (METs, quartiles), educational history (less than 10 years, 10 to 12 years, more than 12 years), dietary energy intake (kcal, quartiles), urinary Na/K ratio and uric acid (mg/dL).
Trend p values were adjusted by FDR(False Discovery Rate) in each qurtile (123 penalties per analysis).
