# Supplementary material for: A population-based urinary and plasma metabolomics study of environmental exposure to cadmium
Source: Environ Health Prev Med. 2024 Mar 30;29:22. doi: 10.1265/ehpm.23-00218 (PMC10992994; doi:10.1265/ehpm.23-00218)
Supplement: Supplementary file 22 — Additional file 22: S Figure 22 Association between urine metabolites and cadmium exposure groups excluding participants of HbA1c is <6.5% or prescribing diabetes medication in men. [file ehpm-29-022-s022.pdf]

S Figure 22 Association between urine metabolites and cadmium exposure groups excluding participants of HbA1c is < 6.5% or prescribing diabetes medication in men.

Forest plot in multivariate regression model (adjusted model)

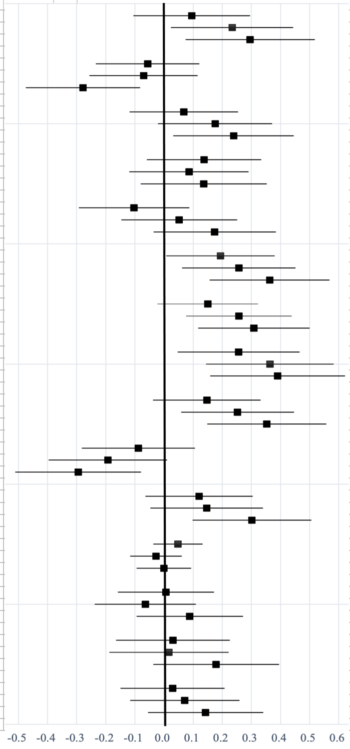

Metabolites	Cd Exposure	Standardized β	95%CI		trend FDR p
Trimethylamine N-oxide	Q2	0.095	-0.104	0.295	
	Q3	0.234	0.025	0.443	2.85E-03
	Q4	0.296	0.075	0.517	
Ala	Q2	-0.056	-0.232	0.121	
	Q3	-0.070	-0.255	0.115	1.93E-02
	Q4	-0.278	-0.473	-0.083	
Choline	Q2	0.068	-0.117	0.254	
	Q3	0.176	-0.019	0.371	1.01E-02
	Q4	0.239	0.033	0.444	
Taurine	Q2	0.138	-0.058	0.333	
	Q3	0.086	-0.118	0.290	1.39E-01
	Q4	0.137	-0.079	0.352	
1-Methylnicotinamide	Q2	-0.103	-0.291	0.086	
	Q3	0.052	-0.145	0.250	3.56E-03
	Q4	0.174	-0.034	0.383	
ADMA	Q2	0.194	0.009	0.379	
	Q3	0.257	0.063	0.451	9.04E-05
	Q4	0.363	0.158	0.568	
Uridine	Q2	0.150	-0.021	0.322	
	Q3	0.257	0.077	0.437	1.84E-03
	Q4	0.309	0.119	0.499	
Adenosine	Q2	0.257	0.048	0.465	
	Q3	0.364	0.145	0.582	8.55E-04
	Q4	0.390	0.159	0.621	
Ethanolamine	Q2	0.147	-0.036	0.331	
	Q3	0.253	0.060	0.446	1.35E-04
	Q4	0.353	0.149	0.556	
Piperidine	Q2	-0.088	-0.282	0.106	
	Q3	-0.193	-0.395	0.010	5.83E-03
	Q4	-0.295	-0.509	-0.080	
5-Aminolevulinate	Q2	0.121	-0.063	0.304	
	Q3	0.147	-0.045	0.339	2.66E-03
	Q4	0.302	0.099	0.505	
Allantoin	Q2	0.048	-0.035	0.131	
	Q3	-0.028	-0.116	0.060	1.17E-01
	Q4	0.000	-0.093	0.092	
7-Methylguanine	Q2	0.007	-0.157	0.171	
	Q3	-0.064	-0.236	0.108	1.16E-02
	Q4	0.089	-0.093	0.270	
N1-Acetylspermidine	Q2	0.031	-0.164	0.225	
	Q3	0.017	-0.187	0.221	1.55E-01
	Q4	0.179	-0.036	0.394	
N8-Acetylspermidine	Q2	0.030	-0.148	0.207	
	Q3	0.072	-0.115	0.258	2.86E-01
	Q4	0.143	-0.054	0.339	

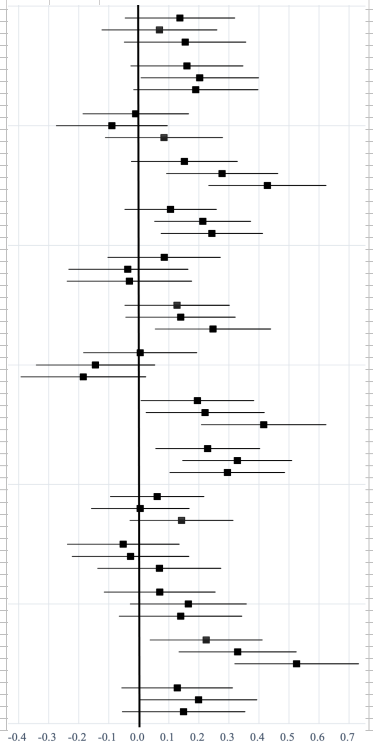

Metabolites	Cd Exposure	Standardized β	95%CI		trend FDR p
Gly-Leu	Q2	0.137	-0.046	0.319	
	Q3	0.068	-0.123	0.260	1.00E-01
	Q4	0.154	-0.048	0.356	
N-epsilon-Acetyllysine	Q2	0.160	-0.027	0.347	
	Q3	0.203	0.007	0.399	3.88E-03
	Q4	0.190	-0.017	0.397	
SAM+	Q2	-0.010	-0.187	0.166	
	Q3	-0.090	-0.275	0.095	4.07E-02
	Q4	0.084	-0.112	0.279	
1-Methyladenosine	Q2	0.151	-0.025	0.328	
	Q3	0.277	0.092	0.463	2.71E-05
	Q4	0.428	0.232	0.623	
Lactate	Q2	0.106	-0.047	0.258	
	Q3	0.213	0.053	0.372	2.38E-03
	Q4	0.243	0.075	0.412	
Malonate	Q2	0.084	-0.103	0.272	
	Q3	-0.037	-0.233	0.160	6.32E-01
	Q4	-0.031	-0.239	0.176	
4-Oxopentanoate	Q2	0.127	-0.047	0.301	
	Q3	0.139	-0.043	0.321	4.09E-03
	Q4	0.247	0.055	0.439	
Succinate	Q2	0.004	-0.184	0.193	
	Q3	-0.145	-0.342	0.053	1.38E-01
	Q4	-0.185	-0.393	0.024	
Isethionate	Q2	0.195	0.007	0.383	
	Q3	0.221	0.024	0.417	3.53E-04
	Q4	0.416	0.209	0.624	
5-Oxoproline	Q2	0.229	0.056	0.402	
	Q3	0.328	0.146	0.509	5.88E-04
	Q4	0.295	0.103	0.486	
Glutarate	Q2	0.061	-0.094	0.217	
	Q3	0.005	-0.158	0.168	4.99E-02
	Q4	0.142	-0.030	0.314	
Malate	Q2	-0.052	-0.238	0.134	
	Q3	-0.028	-0.222	0.167	5.62E-01
	Q4	0.068	-0.137	0.274	
Threonate	Q2	0.070	-0.115	0.254	
	Q3	0.165	-0.029	0.359	3.15E-02
	Q4	0.139	-0.065	0.343	
Ethanolamine phosphate	Q2	0.224	0.037	0.411	
	Q3	0.329	0.134	0.524	3.42E-07
	Q4	0.525	0.319	0.732	
2-Oxoglutarate	Q2	0.128	-0.057	0.312	
	Q3	0.199	0.005	0.393	2.77E-01
	Q4	0.149	-0.055	0.353	

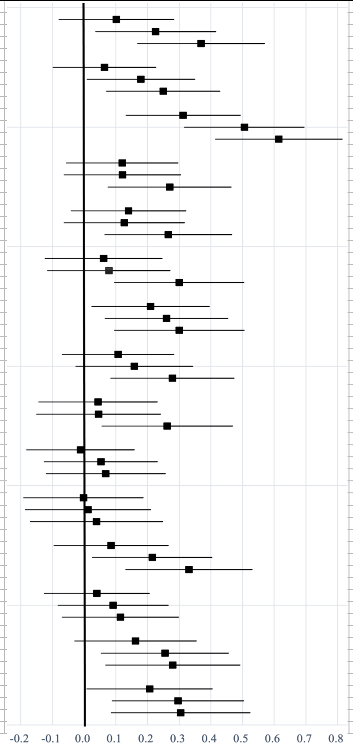

Metabolites	Cd Exposure	Standardized β	95%CI		trend p
Pimelate	Q2	0.102	-0.080	0.283	
	Q3	0.226	0.036	0.415	1.51E-04
	Q4	0.369	0.168	0.569	
Urate	Q2	0.064	-0.098	0.226	
	Q3	0.179	0.010	0.349	6.43E-05
	Q4	0.250	0.071	0.429	
Glycerophosphate	Q2	0.313	0.132	0.493	
	Q3	0.506	0.317	0.695	1.73E-09
	Q4	0.615	0.414	0.815	
trans-Aconitate	Q2	0.120	-0.056	0.296	
	Q3	0.121	-0.063	0.305	3.09E-02
	Q4	0.270	0.076	0.464	
cis-Aconitate	Q2	0.140	-0.041	0.322	
	Q3	0.127	-0.063	0.317	2.75E-02
	Q4	0.266	0.065	0.466	
N-Acetylaspartate	Q2	0.061	-0.124	0.246	
	Q3	0.078	-0.115	0.272	6.51E-03
	Q4	0.300	0.096	0.504	
Azelate	Q2	0.210	0.024	0.395	
	Q3	0.260	0.066	0.454	5.84E-03
	Q4	0.300	0.096	0.505	
Isocitrate	Q2	0.107	-0.069	0.283	
	Q3	0.158	-0.026	0.343	3.12E-03
	Q4	0.279	0.084	0.473	
Adipate	Q2	0.044	-0.143	0.231	
	Q3	0.046	-0.150	0.242	1.38E-02
	Q4	0.262	0.055	0.469	
3-Hydroxy-3-methylglutarate	Q2	-0.011	-0.182	0.159	
	Q3	0.053	-0.126	0.231	5.11E-01
	Q4	0.068	-0.120	0.256	
2,3-Pyridinedicarboxylate	Q2	-0.002	-0.191	0.187	
	Q3	0.012	-0.186	0.210	6.13E-01
	Q4	0.039	-0.170	0.248	
Suberate	Q2	0.085	-0.095	0.266	
	Q3	0.215	0.026	0.404	2.87E-04
	Q4	0.331	0.131	0.530	
Gluconate	Q2	0.040	-0.126	0.206	
	Q3	0.092	-0.082	0.266	2.10E-02
	Q4	0.115	-0.069	0.298	
Saccharate	Q2	0.162	-0.030	0.354	
	Q3	0.255	0.054	0.456	4.98E-03
	Q4	0.280	0.068	0.492	
N-Acetylneuraminate	Q2	0.207	0.008	0.405	
	Q3	0.296	0.089	0.503	7.33E-03
	Q4	0.305	0.086	0.523	

Multivariate regression model was adjusted by age (years),sex ,systolic blood pressure (mmHg), smoke (pack-year), rice intake(bowl/week), BMI (kg/m2), HbA1c (%), LDLc (mg/dL), alcohol consumption (ethanol intake: g/day), physical activity (METs, quartiles), educational history (less than 10 years, 10 to 12 years, more than 12 years), dietary energy intake (kcal, quartiles), urinary Na/K ratio and uric acid (mg/dL).
Trend p values were adjusted by FDR(False Discovery Rate) in each qurtile (123 penalties per analysis).
