# Supplementary material for: A population-based urinary and plasma metabolomics study of environmental exposure to cadmium
Source: Environ Health Prev Med. 2024 Mar 30;29:22. doi: 10.1265/ehpm.23-00218 (PMC10992994; doi:10.1265/ehpm.23-00218)
Supplement: Supplementary file 23 — Additional file 23: S Figure 23 Association between urine metabolites and cadmium exposure groups excluding participants of HbA1c is <6.5% or prescribing diabetes medication in women. [file ehpm-29-022-s023.pdf]

S Figure 23 Association between urine metabolites and cadmium exposure groups excluding participants of HbA1c is < 6.5% or prescribing diabetes medication in women.

Forest plot in multivariate regression model (adjusted model)

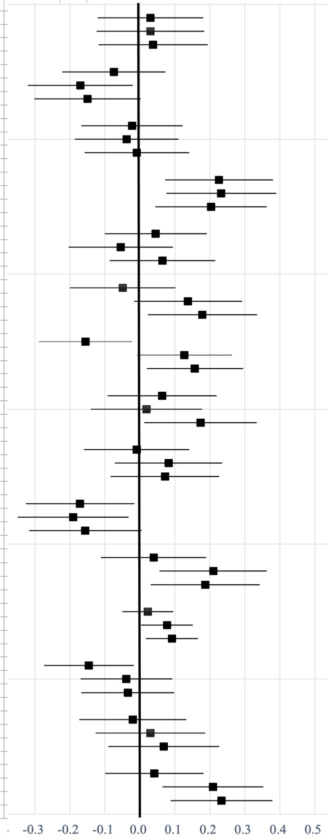

Metabolites	Cd Exposure	Standardized β	95%CI		trend FDR p
Trimethylamine N-oxide	Q2	0.030	-0.120	0.180	
	Q3	0.030	-0.123	0.183	4.59E-01
	Q4	0.038	-0.117	0.193	
Ala	Q2	-0.075	-0.221	0.072	
	Q3	-0.170	-0.319	-0.021	9.99E-02
	Q4	-0.149	-0.300	0.002	
Choline	Q2	-0.022	-0.166	0.122	
	Q3	-0.038	-0.185	0.109	5.47E-01
	Q4	-0.009	-0.158	0.140	
Taurine	Q2	0.226	0.073	0.379	
	Q3	0.233	0.076	0.389	2.26E-02
	Q4	0.203	0.045	0.362	
1-Methylnicotinamide	Q2	0.045	-0.100	0.190	
	Q3	-0.055	-0.203	0.093	2.60E-01
	Q4	0.065	-0.085	0.215	
ADMA	Q2	-0.049	-0.199	0.101	
	Q3	0.138	-0.016	0.291	1.52E-02
	Q4	0.179	0.024	0.334	
Uridine	Q2	-0.155	-0.288	-0.023	
	Q3	0.128	-0.007	0.263	1.50E-02
	Q4	0.157	0.021	0.294	
Adenosine	Q2	0.064	-0.091	0.219	
	Q3	0.019	-0.139	0.177	5.34E-02
	Q4	0.173	0.013	0.333	
Ethanolamine	Q2	-0.009	-0.159	0.140	
	Q3	0.082	-0.070	0.235	3.90E-01
	Q4	0.072	-0.083	0.226	
Piperidine	Q2	-0.171	-0.325	-0.016	
	Q3	-0.191	-0.349	-0.033	1.51E-01
	Q4	-0.156	-0.316	0.004	
5-Aminolevulinate	Q2	0.039	-0.111	0.189	
	Q3	0.210	0.057	0.362	7.88E-04
	Q4	0.187	0.032	0.341	
Allantoin	Q2	0.023	-0.049	0.094	
	Q3	0.077	0.004	0.150	4.99E-02
	Q4	0.092	0.018	0.166	
7-Methylguanine	Q2	-0.146	-0.273	-0.018	
	Q3	-0.038	-0.169	0.092	6.07E-02
	Q4	-0.035	-0.166	0.097	
N1-Acetylspermidine	Q2	-0.020	-0.172	0.132	
	Q3	0.030	-0.125	0.186	1.91E-01
	Q4	0.068	-0.089	0.226	
N8-Acetylspermidine	Q2	0.041	-0.099	0.181	
	Q3	0.209	0.066	0.352	4.20E-05
	Q4	0.234	0.089	0.378	

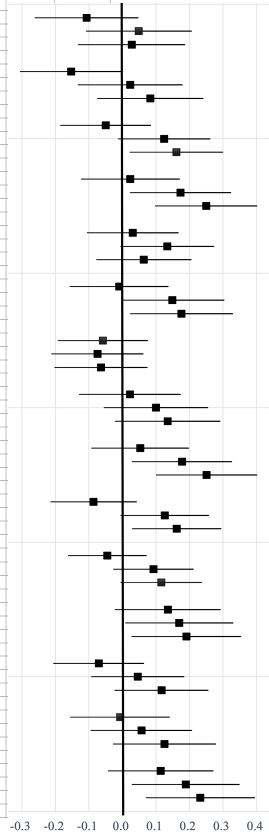

Metabolites	Cd Exposure	Standardized β	95%CI		trend FDR p
Gly-Leu	Q2	-0.107	-0.261	0.046	
	Q3	0.049	-0.108	0.206	1.30E-01
	Q4	0.028	-0.131	0.187	
N-epsilon-Acetyllysine	Q2	-0.153	-0.305	-0.001	
	Q3	0.023	-0.132	0.178	1.68E-02
	Q4	0.083	-0.074	0.241	
SAM+	Q2	-0.050	-0.184	0.084	
	Q3	0.125	-0.012	0.262	3.75E-04
	Q4	0.161	0.023	0.300	
1-Methyladenosine	Q2	0.024	-0.123	0.170	
	Q3	0.173	0.023	0.323	4.16E-03
	Q4	0.250	0.098	0.401	
Lactate	Q2	0.031	-0.105	0.167	
	Q3	0.134	-0.005	0.273	2.06E-01
	Q4	0.064	-0.077	0.205	
Malonate	Q2	-0.010	-0.157	0.137	
	Q3	0.149	-0.001	0.300	1.12E-03
	Q4	0.176	0.024	0.329	
4-Oxopentanoate	Q2	-0.058	-0.191	0.075	
	Q3	-0.075	-0.211	0.061	9.67E-01
	Q4	-0.064	-0.202	0.074	
Succinate	Q2	0.022	-0.129	0.174	
	Q3	0.101	-0.054	0.255	4.26E-03
	Q4	0.135	-0.022	0.291	
Isethionate	Q2	0.053	-0.092	0.198	
	Q3	0.177	0.029	0.326	2.76E-03
	Q4	0.251	0.101	0.401	
5-Oxoproline	Q2	-0.086	-0.214	0.042	
	Q3	0.126	-0.004	0.257	4.90E-02
	Q4	0.162	0.030	0.295	
Glutarate	Q2	-0.045	-0.161	0.072	
	Q3	0.093	-0.026	0.211	6.05E-02
	Q4	0.116	-0.004	0.236	
Malate	Q2	0.135	-0.022	0.292	
	Q3	0.170	0.010	0.330	1.53E-02
	Q4	0.191	0.028	0.353	
Threonate	Q2	-0.071	-0.205	0.064	
	Q3	0.046	-0.092	0.183	4.88E-03
	Q4	0.117	-0.023	0.256	
Ethanolamine phosphate	Q2	-0.007	-0.155	0.141	
	Q3	0.057	-0.094	0.207	1.41E-02
	Q4	0.125	-0.028	0.278	
2-Oxoglutarate	Q2	0.114	-0.042	0.271	
	Q3	0.189	0.029	0.349	3.14E-03
	Q4	0.233	0.071	0.395	

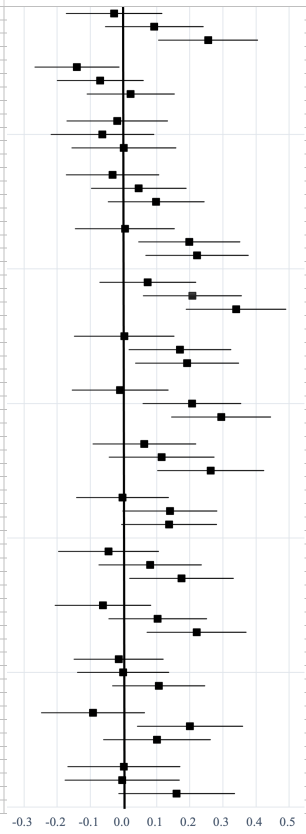

Metabolites	Cd Exposure	Standardized β	95%CI		trend FDR p
Pimelate	Q2	-0.028	-0.173	0.116	
	Q3	0.093	-0.055	0.240	1.43E-04
	Q4	0.255	0.106	0.405	
Urate	Q2	-0.140	-0.267	-0.013	
	Q3	-0.070	-0.200	0.060	2.36E-03
	Q4	0.022	-0.110	0.153	
Glycerophosphate	Q2	-0.019	-0.171	0.133	
	Q3	-0.063	-0.219	0.092	6.30E-01
	Q4	0.001	-0.156	0.158	
trans-Aconitate	Q2	-0.033	-0.172	0.107	
	Q3	0.046	-0.097	0.189	2.02E-01
	Q4	0.099	-0.046	0.243	
cis-Aconitate	Q2	0.004	-0.145	0.154	
	Q3	0.199	0.046	0.351	2.55E-03
	Q4	0.222	0.068	0.377	
N-Acetylaspartate	Q2	0.073	-0.072	0.218	
	Q3	0.208	0.060	0.356	1.86E-06
	Q4	0.340	0.190	0.490	
Azelate	Q2	0.002	-0.148	0.153	
	Q3	0.171	0.017	0.324	3.23E-03
	Q4	0.192	0.036	0.348	
Isocitrate	Q2	-0.010	-0.155	0.135	
	Q3	0.207	0.059	0.354	3.92E-06
	Q4	0.295	0.145	0.444	
Adipate	Q2	0.063	-0.092	0.218	
	Q3	0.115	-0.043	0.273	1.54E-03
	Q4	0.263	0.103	0.423	
3-Hydroxy-3-methylglutarate	Q2	-0.003	-0.142	0.135	
	Q3	0.140	-0.002	0.282	2.46E-03
	Q4	0.138	-0.006	0.281	
2,3-Pyridinedicarboxylate	Q2	-0.045	-0.196	0.106	
	Q3	0.081	-0.074	0.235	4.42E-03
	Q4	0.175	0.019	0.331	
Suberate	Q2	-0.062	-0.206	0.083	
	Q3	0.103	-0.044	0.251	7.72E-04
	Q4	0.221	0.071	0.370	
Gluconate	Q2	-0.014	-0.149	0.120	
	Q3	-0.001	-0.138	0.136	5.36E-02
	Q4	0.106	-0.033	0.245	
Saccharate	Q2	-0.092	-0.247	0.064	
	Q3	0.201	0.042	0.360	4.26E-02
	Q4	0.101	-0.060	0.262	
N-Acetylneuraminate	Q2	0.001	-0.168	0.170	
	Q3	-0.004	-0.177	0.169	9.76E-02
	Q4	0.160	-0.014	0.335	

Multivariate regression model was adjusted by age (years),sex ,systolic blood pressure (mmHg), smoke (pack-year), rice intake(bowl/week), BMI (kg/m2), HbA1c (%), LDLc (mg/dL), alcohol consumption (ethanol intake: g/day), physical activity (METs, quartiles), educational history (less than 10 years, 10 to 12 years, more than 12 years), dietary energy intake (kcal, quartiles), urinary Na/K ratio and uric acid (mg/dL).
Trend p values were adjusted by FDR(False Discovery Rate) in each qurtile (123 penalties per analysis).
