# Supplementary material for: A population-based urinary and plasma metabolomics study of environmental exposure to cadmium
Source: Environ Health Prev Med. 2024 Mar 30;29:22. doi: 10.1265/ehpm.23-00218 (PMC10992994; doi:10.1265/ehpm.23-00218)
Supplement: Supplementary file 24 — Additional file 24: S Figure 24 Association between plasma metabolites and cadmium exposure groups excluding participants of HbA1c is <6.5% or prescribing diabetes medication. [file ehpm-29-022-s024.pdf]

S Figure 24 Association between plasma metabolites and cadmium exposure groups excluding participants of HbA1c is < 6.5% or prescribing diabetes medication in women.

Variables

Forest plot in multivariate regression model (adjusted model)

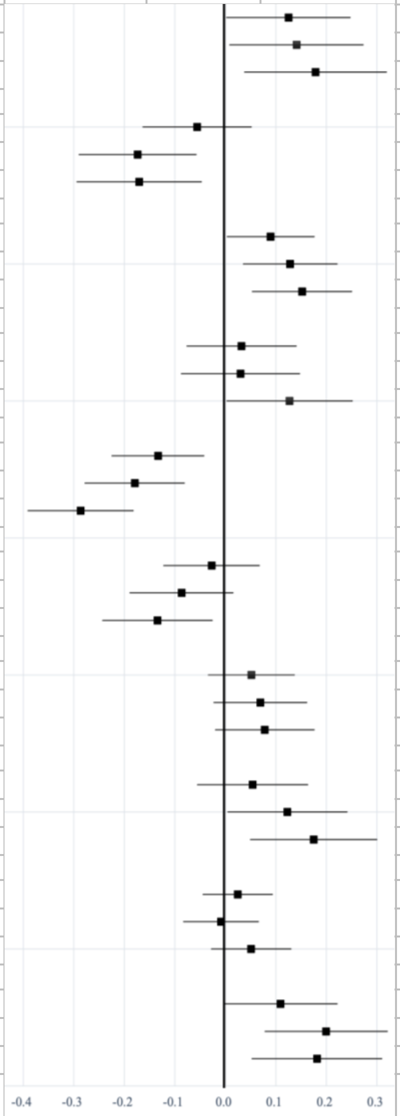

Metabolites	Cd Exposure	coefficient	95%CI		trend FDR p
Trimethylamine N-oxide	Q2	0.125	0.002	0.248	5.58E-03
	Q3	0.141	0.009	0.273	
	Q4	0.178	0.038	0.319	
Ala	Q2	-0.056	-0.163	0.051	5.19E-03
	Q3	-0.173	-0.289	-0.057	
	Q4	-0.170	-0.294	-0.047	
Sarcosine	Q2	0.090	0.004	0.176	8.07E-05
	Q3	0.128	0.036	0.221	
	Q4	0.152	0.054	0.251	
N,N-Dimethylglycine	Q2	0.032	-0.076	0.141	5.42E-04
	Q3	0.030	-0.087	0.147	
	Q4	0.127	0.003	0.252	
Creatinine	Q2	-0.133	-0.224	-0.042	2.89E-06
	Q3	-0.179	-0.277	-0.081	
	Q4	-0.286	-0.390	-0.182	
Ile	Q2	-0.027	-0.122	0.068	8.73E-03
	Q3	-0.086	-0.189	0.016	
	Q4	-0.134	-0.243	-0.026	
Asp	Q2	0.052	-0.033	0.137	6.88E-04
	Q3	0.069	-0.023	0.162	
	Q4	0.078	-0.020	0.176	
Carnitine	Q2	0.054	-0.055	0.163	1.43E-03
	Q3	0.123	0.005	0.241	
	Q4	0.175	0.050	0.300	
Uridine	Q2	0.024	-0.044	0.093	1.41E-03
	Q3	-0.008	-0.083	0.066	
	Q4	0.051	-0.028	0.130	
2-Oxoglutarate	Q2	0.110	-0.002	0.222	7.23E-04
	Q3	0.200	0.079	0.321	
	Q4	0.181	0.053	0.310	

Multivariate regression model was adjusted by age (years), sex, systolic blood pressure (mmHg), smoke (pack-year), rice intake(bowl/week), BMI (kg/m2), HbA1c (%), LDLc (mg/dL), alcohol consumption (ethanol intake: g/day), physical activity (METs, quartiles), educational history (less than 10 years, 10 to 12 years, more than 12 years), dietary energy intake (kcal, quartiles), urinary Na/K ratio and uric acid (mg/dL).
P values were adjusted by FDR(False Discovery Rate) in each qurtile (94 penalties per analysis).
