# Supplementary material for: A population-based urinary and plasma metabolomics study of environmental exposure to cadmium
Source: Environ Health Prev Med. 2024 Mar 30;29:22. doi: 10.1265/ehpm.23-00218 (PMC10992994; doi:10.1265/ehpm.23-00218)
Supplement: Supplementary file 25 — Additional file 25: S Figure 25 Association between urine metabolites and cadmium exposure groups excluding participants of HbA1c is <6.5% or prescribing diabetes medication in sex stratification. [file ehpm-29-022-s025.pdf]

S Figure 25 Association between plasma metabolites and cadmium exposure groups excluding participants of HbA1c is < 6.5% or prescribing diabetes medication in sex strastification.

Men Variables

Forest plot in univariate regression model (adjusted model)

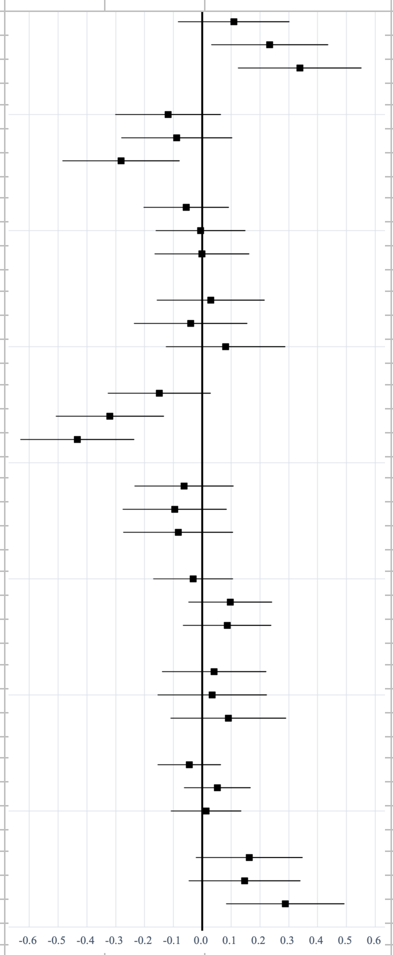

Metabolites	Cd Exposure	Standardized β	95%CI		trend FDR p
Trimethylamine N-oxi	Q2	0.109	-0.083	0.302	4.42E-04
	Q3	0.234	0.032	0.436	
	Q4	0.338	0.124	0.552	
Ala	Q2	-0.119	-0.301	0.064	2.05E-02
	Q3	-0.089	-0.280	0.103	
	Q4	-0.281	-0.484	-0.079	
Sarcosine	Q2	-0.056	-0.203	0.091	4.63E-02
	Q3	-0.006	-0.161	0.148	
	Q4	-0.001	-0.164	0.162	
N,N-Dimethylglycine	Q2	0.029	-0.157	0.215	1.78E-01
	Q3	-0.040	-0.235	0.156	
	Q4	0.081	-0.126	0.287	
Creatinine	Q2	-0.149	-0.326	0.028	6.11E-05
	Q3	-0.320	-0.507	-0.134	
	Q4	-0.434	-0.630	-0.237	
Ile	Q2	-0.063	-0.234	0.107	4.70E-01
	Q3	-0.095	-0.275	0.084	
	Q4	-0.083	-0.273	0.106	
Asp	Q2	-0.032	-0.169	0.105	4.36E-02
	Q3	0.097	-0.047	0.241	
	Q4	0.086	-0.066	0.238	
Carnitine	Q2	0.041	-0.138	0.221	2.37E-01
	Q3	0.035	-0.154	0.223	
	Q4	0.091	-0.109	0.290	
Uridine	Q2	-0.045	-0.154	0.064	2.84E-01
	Q3	0.052	-0.062	0.167	
	Q4	0.013	-0.108	0.134	
2-Oxoglutarate	Q2	0.163	-0.021	0.347	1.49E-02
	Q3	0.146	-0.046	0.339	
	Q4	0.288	0.084	0.492	

Women Variables

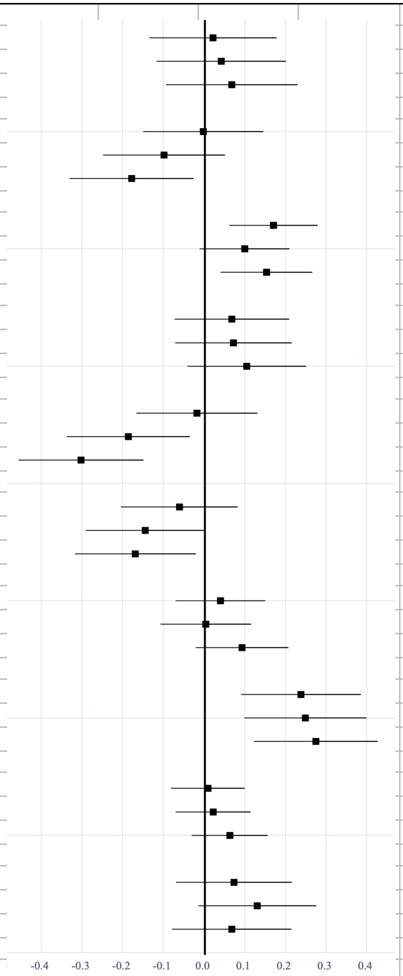

Metabolites	Cd Exposure	Standardized β	95%CI		trend FDR p
Trimethylamine	Q2	0.022	-0.134	0.178	2.34E-01
	Q3	0.042	-0.116	0.201	
	Q4	0.068	-0.093	0.229	
Ala	Q2	-0.002	-0.148	0.145	1.14E-02
	Q3	-0.098	-0.247	0.051	
	Q4	-0.178	-0.330	-0.027	
Sarcosine	Q2	0.171	0.062	0.279	7.98E-03
	Q3	0.100	-0.010	0.210	
	Q4	0.154	0.042	0.265	
N,N-Dimethylg	Q2	0.068	-0.072	0.209	3.11E-03
	Q3	0.072	-0.070	0.215	
	Q4	0.105	-0.040	0.250	
Creatinine	Q2	-0.018	-0.166	0.131	2.06E-04
	Q3	-0.186	-0.337	-0.036	
	Q4	-0.302	-0.455	-0.149	
Ile	Q2	-0.061	-0.204	0.083	7.60E-03
	Q3	-0.145	-0.290	0.001	
	Q4	-0.169	-0.317	-0.021	
Asp	Q2	0.040	-0.069	0.150	5.30E-04
	Q3	0.004	-0.107	0.115	
	Q4	0.093	-0.020	0.207	
Carnitine	Q2	0.238	0.091	0.385	5.31E-04
	Q3	0.249	0.100	0.398	
	Q4	0.274	0.123	0.426	
Uridine	Q2	0.009	-0.081	0.099	2.03E-04
	Q3	0.022	-0.069	0.114	
	Q4	0.063	-0.030	0.156	
2-Oxoglutarate	Q2	0.074	-0.068	0.215	1.03E-01
	Q3	0.131	-0.013	0.275	
	Q4	0.068	-0.078	0.214	

Multivariate regression model was adjusted by age (years), sex, systolic blood pressure (mmHg), smoke (pack-year), rice intake(bowl/week), BMI (kg/m2), HbA1c (%), LDLc (mg/dL), alcohol consumption (ethanol intake: g/day), physical activity (METs, quartiles), educational history (less than 10 years, 10 to 12 years, more than 12 years), dietary energy intake (kcal, quartiles), urinary Na/K ratio and uric acid (mg/dL).
P values were adjusted by FDR(False Discovery Rate) in each qurtile (94 penalties per analysis).
