# Supplementary material for: A population-based urinary and plasma metabolomics study of environmental exposure to cadmium
Source: Environ Health Prev Med. 2024 Mar 30;29:22. doi: 10.1265/ehpm.23-00218 (PMC10992994; doi:10.1265/ehpm.23-00218)
Supplement: Supplementary file 26 — Additional file 26: S Figure 26 Association between urine metabolites and cadmium exposure groups excluding drinkers. [file ehpm-29-022-s026.pdf]

S Figure 26 Association between urine metabolites and cadmium exposure groups excluding drinkers.

Forest plot in multivariate regression model (adjusted model)

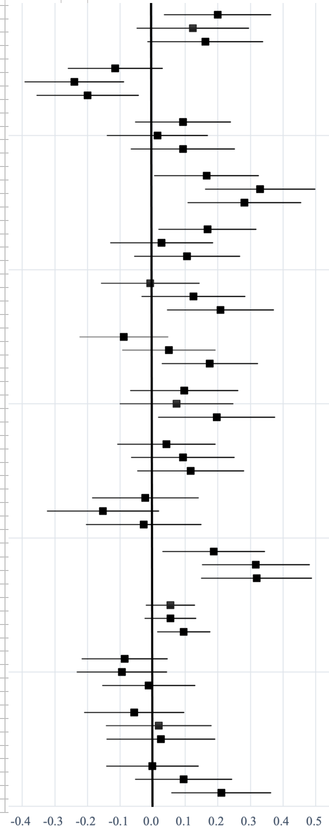

Metabolites	Cd Exposure	Standardized β	95%CI		trend FDR p
	Q2	0.199	0.036	0.362	
Trimethylamine N-oxide	Q3	0.123	-0.048	0.295	1.17E-01
	Q4	0.162	-0.015	0.338	
	Q2	-0.115	-0.259	0.030	
Ala	Q3	-0.240	-0.392	-0.089	6.51E-02
	Q4	-0.199	-0.356	-0.043	
	Q2	0.093	-0.053	0.239	
Choline	Q3	0.014	-0.139	0.168	4.72E-02
	Q4	0.093	-0.066	0.251	
	Q2	0.165	0.005	0.325	
Taurine	Q3	0.330	0.162	0.498	4.26E-04
	Q4	0.281	0.109	0.454	
	Q2	0.168	0.019	0.317	
1-Methylnicotinamide	Q3	0.027	-0.130	0.184	1.36E-01
	Q4	0.105	-0.056	0.267	
	Q2	-0.007	-0.158	0.143	
ADMA	Q3	0.125	-0.033	0.283	5.01E-03
	Q4	0.208	0.045	0.371	
	Q2	-0.088	-0.223	0.047	
Uridine	Q3	0.049	-0.093	0.192	5.89E-02
	Q4	0.175	0.029	0.322	
	Q2	0.096	-0.068	0.261	
Adenosine	Q3	0.073	-0.100	0.247	4.51E-02
	Q4	0.196	0.018	0.374	
	Q2	0.042	-0.108	0.192	
Ethanolamine	Q3	0.093	-0.065	0.251	1.71E-01
	Q4	0.117	-0.046	0.279	
	Q2	-0.022	-0.185	0.140	
Piperidine	Q3	-0.152	-0.323	0.019	6.89E-01
	Q4	-0.028	-0.203	0.148	
	Q2	0.187	0.031	0.343	
5-Aminolevulinate	Q3	0.317	0.152	0.481	1.26E-05
	Q4	0.319	0.149	0.488	
	Q2	0.054	-0.020	0.128	
Allantoin	Q3	0.054	-0.023	0.132	1.41E-02
	Q4	0.095	0.015	0.175	
	Q2	-0.086	-0.217	0.045	
7-Methylguanine	Q3	-0.094	-0.232	0.043	1.41E-02
	Q4	-0.012	-0.154	0.129	
	Q2	-0.057	-0.210	0.096	
N1-Acetylspermidine	Q3	0.019	-0.142	0.180	2.07E-01
	Q4	0.025	-0.141	0.191	
	Q2	-0.001	-0.141	0.140	
N8-Acetylspermidine	Q3	0.095	-0.053	0.243	1.24E-04
	Q4	0.210	0.058	0.363	

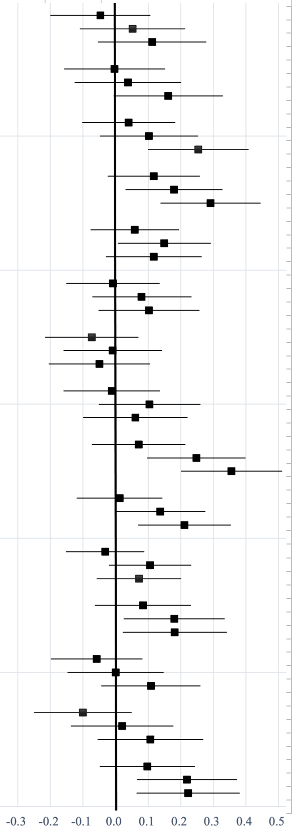

Metabolites	Cd Exposure	Standardized β	95%CI		trend FDR p
	Q2	-0.047	-0.199	0.105	
Gly-Leu	Q3	0.052	-0.109	0.212	6.82E-03
	Q4	0.112	-0.053	0.277	
	Q2	-0.003	-0.157	0.151	
N-epsilon-Acetyllysine	Q3	0.038	-0.124	0.200	2.69E-03
	Q4	0.161	-0.006	0.328	
	Q2	0.040	-0.102	0.182	
SAM+	Q3	0.102	-0.047	0.251	8.13E-06
	Q4	0.254	0.100	0.407	
	Q2	0.117	-0.024	0.257	
1-Methyladenosine	Q3	0.180	0.032	0.327	1.45E-03
	Q4	0.291	0.139	0.444	
	Q2	0.059	-0.076	0.194	
Lactate	Q3	0.150	0.008	0.292	6.12E-02
	Q4	0.117	-0.029	0.263	
	Q2	-0.008	-0.150	0.134	
Malonate	Q3	0.079	-0.070	0.229	1.27E-02
	Q4	0.102	-0.052	0.257	
	Q2	-0.073	-0.215	0.070	
4-Oxopentanoate	Q3	-0.009	-0.159	0.141	5.54E-01
	Q4	-0.050	-0.204	0.105	
	Q2	-0.012	-0.159	0.135	
Succinate	Q3	0.104	-0.051	0.259	3.20E-02
	Q4	0.061	-0.099	0.220	
	Q2	0.071	-0.072	0.213	
Isethionate	Q3	0.248	0.098	0.398	2.31E-05
	Q4	0.356	0.202	0.510	
	Q2	0.012	-0.118	0.143	
5-Oxoproline	Q3	0.137	-0.001	0.274	1.00E-01
	Q4	0.212	0.070	0.353	
	Q2	-0.032	-0.151	0.087	
Glutarate	Q3	0.106	-0.019	0.231	1.77E-01
	Q4	0.072	-0.057	0.200	
	Q2	0.084	-0.063	0.230	
Malate	Q3	0.180	0.026	0.334	5.06E-03
	Q4	0.182	0.023	0.340	
	Q2	-0.058	-0.197	0.081	
Threonate	Q3	0.000	-0.146	0.147	2.86E-02
	Q4	0.108	-0.042	0.259	
	Q2	-0.100	-0.249	0.048	
Ethanolamine phosphate	Q3	0.020	-0.136	0.177	1.33E-02
	Q4	0.107	-0.054	0.268	
	Q2	0.097	-0.048	0.242	
2-Oxoglutarate	Q3	0.219	0.066	0.372	1.71E-03
	Q4	0.222	0.065	0.380	

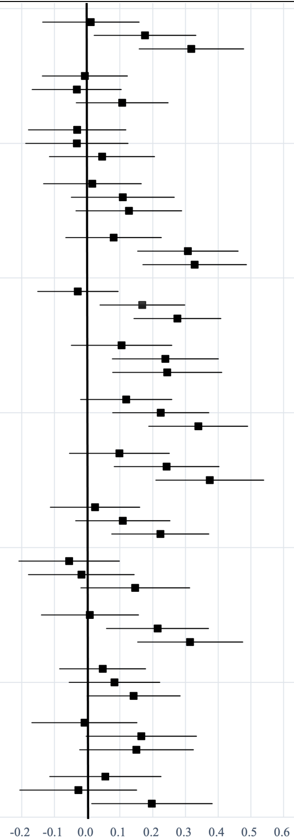

Metabolites	Cd Exposure	Standardized β	95%CI		trend FDR p
	Q2	0.011	-0.136	0.158	
Pimelate	Q3	0.177	0.022	0.332	9.43E-06
	Q4	0.318	0.159	0.478	
	Q2	-0.007	-0.137	0.122	
Urate	Q3	-0.032	-0.168	0.104	4.02E-04
	Q4	0.106	-0.034	0.247	
	Q2	-0.030	-0.179	0.118	
Glycerophosphate	Q3	-0.032	-0.188	0.125	2.59E-01
	Q4	0.045	-0.116	0.206	
	Q2	0.016	-0.133	0.165	
trans-Aconitate	Q3	0.109	-0.048	0.266	1.20E-01
	Q4	0.127	-0.034	0.289	
	Q2	0.081	-0.065	0.227	
cis-Aconitate	Q3	0.307	0.154	0.461	8.33E-06
	Q4	0.328	0.170	0.486	
	Q2	-0.028	-0.151	0.095	
N-Acetylaspartate	Q3	0.168	0.039	0.298	2.34E-07
	Q4	0.275	0.142	0.409	
	Q2	0.105	-0.049	0.259	
Azelate	Q3	0.238	0.077	0.400	3.08E-03
	Q4	0.244	0.078	0.411	
	Q2	0.119	-0.020	0.259	
Isocitrate	Q3	0.225	0.078	0.371	1.13E-06
	Q4	0.339	0.188	0.490	
	Q2	0.098	-0.054	0.251	
Adipate	Q3	0.243	0.083	0.403	2.53E-06
	Q4	0.375	0.210	0.539	
	Q2	0.024	-0.113	0.161	
3-Hydroxy-3-methylglutarate	Q3	0.109	-0.035	0.253	7.79E-05
	Q4	0.223	0.075	0.372	
	Q2	-0.055	-0.208	0.099	
2,3-Pyridinedicarboxylate	Q3	-0.018	-0.179	0.144	3.99E-02
	Q4	0.147	-0.019	0.313	
	Q2	0.008	-0.140	0.156	
Suberate	Q3	0.215	0.059	0.371	2.84E-05
	Q4	0.314	0.154	0.475	
	Q2	0.047	-0.084	0.179	
Gluconate	Q3	0.084	-0.055	0.222	1.94E-02
	Q4	0.142	0.000	0.284	
	Q2	-0.008	-0.169	0.152	2.96E-02
Saccharate	Q3	0.166	-0.003	0.334	
	Q4	0.151	-0.023	0.324	
	Q2	0.055	-0.115	0.225	7.82E-02
N-Acetylneuraminate	Q3	-0.027	-0.206	0.152	
	Q4	0.198	0.014	0.382	

Multivariate regression model was adjusted by age (years),sex ,systolic blood pressure (mmHg), smoke (pack-year), rice intake(bowl/week), BMI (kg/m2), HbA1c (%), LDLc (mg/dL), alcohol consumption (ethanol intake: g/day), physical activity (METs, quartiles), educational history (less than 10 years, 10 to 12 years, more than 12 years), dietary energy intake (kcal, quartiles), urinary Na/K ratio and uric acid (mg/dL).
Trend p values were adjusted by FDR(False Discovery Rate) in each qurtile (123 penalties per analysis).
