# Supplementary material for: A population-based urinary and plasma metabolomics study of environmental exposure to cadmium
Source: Environ Health Prev Med. 2024 Mar 30;29:22. doi: 10.1265/ehpm.23-00218 (PMC10992994; doi:10.1265/ehpm.23-00218)
Supplement: Supplementary file 27 — Additional file 27: S Figure 27 Association between urine metabolites and cadmium exposure groups excluding drinkers in men. [file ehpm-29-022-s027.pdf]

S Figure 27 Association between urine metabolites and cadmium exposure groups excluding drinkers in men.

Forest plot in multivariate regression model (adjusted model)

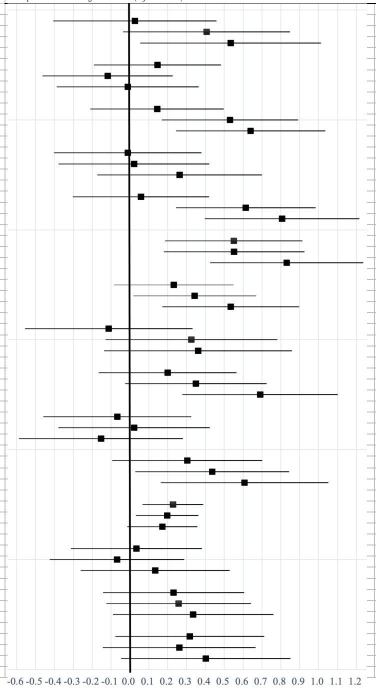

Metabolites	Cd Exposure	Standardized β	95%CI		trend FDR p
Trimethylamine N-oxide	Q2	0.027	-0.405	0.458	1.16E-02
	Q3	0.406	-0.035	0.847	
	Q4	0.534	0.056	1.012	
Ala	Q2	0.146	-0.190	0.482	7.55E-01
	Q3	-0.117	-0.461	0.226	
	Q4	-0.011	-0.386	0.363	
Choline	Q2	0.145	-0.207	0.496	5.71E-04
	Q3	0.530	0.170	0.891	
	Q4	0.640	0.246	1.034	
Taurine	Q2	-0.011	-0.401	0.378	1.50E-01
	Q3	0.022	-0.377	0.420	
	Q4	0.264	-0.171	0.699	
1-Methylnicotinamide	Q2	0.059	-0.300	0.419	8.02E-06
	Q3	0.614	0.245	0.983	
	Q4	0.806	0.398	1.215	
ADMA	Q2	0.551	0.188	0.913	5.32E-04
	Q3	0.552	0.181	0.923	
	Q4	0.831	0.426	1.235	
Uridine	Q2	0.232	-0.083	0.547	8.47E-02
	Q3	0.343	0.019	0.667	
	Q4	0.534	0.173	0.895	
Adenosine	Q2	-0.112	-0.555	0.331	4.81E-02
	Q3	0.326	-0.127	0.780	
	Q4	0.362	-0.135	0.858	
Ethanolamine	Q2	0.200	-0.164	0.564	2.41E-03
	Q3	0.349	-0.024	0.722	
	Q4	0.690	0.280	1.101	
Piperidine	Q2	-0.066	-0.457	0.325	5.96E-01
	Q3	0.022	-0.378	0.422	
	Q4	-0.153	-0.587	0.280	
5-Aminolevulinate	Q2	0.304	-0.092	0.701	8.94E-03
	Q3	0.436	0.030	0.842	
	Q4	0.607	0.164	1.050	
Allantoin	Q2	0.228	0.069	0.387	4.33E-01
	Q3	0.197	0.033	0.361	
	Q4	0.172	-0.013	0.357	
7-Methylguanine	Q2	0.035	-0.312	0.381	3.51E-01
	Q3	-0.068	-0.423	0.287	
	Q4	0.134	-0.259	0.528	
N1-Acetylspermidine	Q2	0.231	-0.141	0.604	1.78E-01
	Q3	0.258	-0.124	0.641	
	Q4	0.335	-0.088	0.759	
N8-Acetylspermidine	Q2	0.317	-0.077	0.711	7.31E-02
	Q3	0.262	-0.142	0.665	
	Q4	0.402	-0.044	0.849	

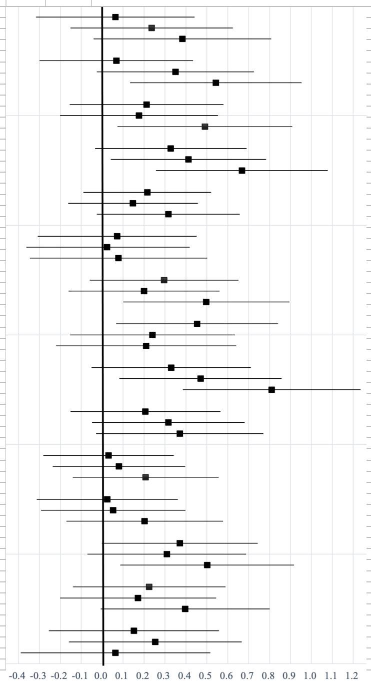

Metabolites	Cd Exposure	Standardized β	95%CI		trend FDR p
Gly-Leu	Q2	0.062	-0.315	0.440	3.13E-02
	Q3	0.236	-0.151	0.623	
	Q4	0.383	-0.040	0.806	
N-epsilon-Acetyllysine	Q2	0.068	-0.298	0.433	2.88E-03
	Q3	0.350	-0.024	0.724	
	Q4	0.543	0.135	0.951	
SAM+	Q2	0.212	-0.155	0.578	3.50E-02
	Q3	0.176	-0.200	0.552	
	Q4	0.491	0.074	0.907	
1-Methyladenosine	Q2	0.328	-0.033	0.688	1.73E-02
	Q3	0.413	0.043	0.783	
	Q4	0.668	0.258	1.077	
Lactate	Q2	0.215	-0.088	0.519	4.44E-02
	Q3	0.147	-0.162	0.455	
	Q4	0.316	-0.024	0.656	
Malonate	Q2	0.072	-0.307	0.450	5.80E-01
	Q3	0.024	-0.361	0.409	
	Q4	0.078	-0.345	0.500	
4-Oxopentanoate	Q2	0.296	-0.059	0.650	4.22E-02
	Q3	0.200	-0.160	0.560	
	Q4	0.498	0.102	0.893	
Succinate	Q2	0.453	0.067	0.839	5.17E-01
	Q3	0.240	-0.153	0.633	
	Q4	0.210	-0.220	0.639	
Isethionate	Q2	0.330	-0.050	0.710	6.64E-04
	Q3	0.470	0.083	0.857	
	Q4	0.810	0.386	1.233	
5-Oxoproline	Q2	0.207	-0.150	0.563	1.01E-01
	Q3	0.316	-0.047	0.679	
	Q4	0.371	-0.028	0.769	
Glutarate	Q2	0.031	-0.280	0.341	1.30E-01
	Q3	0.080	-0.236	0.395	
	Q4	0.208	-0.140	0.556	
Malate	Q2	0.024	-0.312	0.360	2.84E-01
	Q3	0.053	-0.291	0.396	
	Q4	0.203	-0.170	0.575	
Threonate	Q2	0.371	0.000	0.743	2.90E-02
	Q3	0.309	-0.069	0.687	
	Q4	0.502	0.087	0.916	
Ethanolamine phosphate	Q2	0.225	-0.139	0.588	1.01E-01
	Q3	0.171	-0.200	0.543	
	Q4	0.397	-0.006	0.800	
2-Oxoglutarate	Q2	0.152	-0.253	0.557	7.68E-01
	Q3	0.254	-0.158	0.666	
	Q4	0.063	-0.388	0.515	

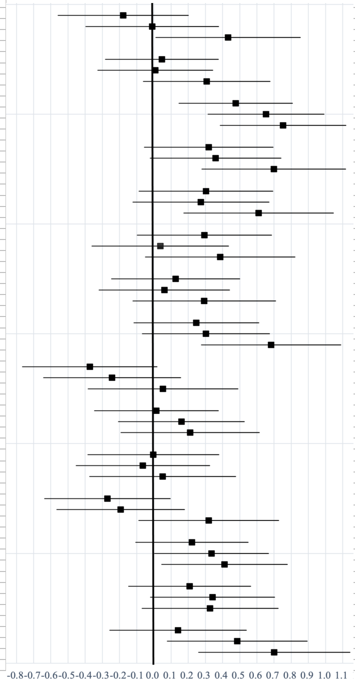

Metabolites	Cd Exposure	Standardized β	95%CI		trend FDR p
Pimelate	Q2	-0.179	-0.558	0.200	2.37E-02
	Q3	-0.010	-0.397	0.377	
	Q4	0.432	0.011	0.853	
Urate	Q2	0.047	-0.283	0.376	5.49E-02
	Q3	0.008	-0.327	0.343	
	Q4	0.308	-0.061	0.676	
Glycerophosphate	Q2	0.477	0.147	0.808	2.30E-04
	Q3	0.653	0.315	0.991	
	Q4	0.752	0.386	1.119	
trans-Aconitate	Q2	0.320	-0.055	0.695	4.48E-03
	Q3	0.360	-0.021	0.741	
	Q4	0.699	0.280	1.117	
cis-Aconitate	Q2	0.303	-0.086	0.693	3.45E-02
	Q3	0.274	-0.122	0.671	
	Q4	0.610	0.174	1.045	
N-Acetylaspartate	Q2	0.294	-0.097	0.685	2.27E-01
	Q3	0.037	-0.361	0.435	
	Q4	0.386	-0.050	0.822	
Azelate	Q2	0.127	-0.246	0.500	1.81E-01
	Q3	0.061	-0.319	0.441	
	Q4	0.293	-0.123	0.709	
Isocitrate	Q2	0.248	-0.117	0.612	1.54E-03
	Q3	0.303	-0.068	0.673	
	Q4	0.683	0.276	1.089	
Adipate	Q2	-0.373	-0.765	0.019	4.35E-01
	Q3	-0.243	-0.642	0.156	
	Q4	0.053	-0.384	0.490	
3-Hydroxy-3-methylglutarate	Q2	0.015	-0.346	0.376	2.77E-01
	Q3	0.161	-0.206	0.528	
	Q4	0.211	-0.193	0.614	
2,3-Pyridinedicarboxylate	Q2	-0.003	-0.385	0.379	8.38E-01
	Q3	-0.064	-0.453	0.325	
	Q4	0.051	-0.374	0.476	
Suberate	Q2	-0.271	-0.637	0.096	8.79E-02
	Q3	-0.193	-0.566	0.179	
	Q4	0.320	-0.088	0.728	
Gluconate	Q2	0.222	-0.105	0.549	1.40E-02
	Q3	0.336	0.004	0.668	
	Q4	0.412	0.046	0.778	
Saccharate	Q2	0.208	-0.147	0.564	5.60E-02
	Q3	0.342	-0.020	0.704	
	Q4	0.327	-0.069	0.724	
N-Acetylneuraminate	Q2	0.141	-0.257	0.539	9.05E-04
	Q3	0.486	0.078	0.893	
	Q4	0.701	0.260	1.143	

Multivariate regression model was adjusted by age (years),sex ,systolic blood pressure (mmHg), smoke (pack-year), rice intake(bowl/week), BMI (kg/m2), HbA1c (%), LDLc (mg/dL), alcohol consumption (ethanol intake: g/day), physical activity (METs, quartiles), educational history (less than 10 years, 10 to 12 years, more than 12 years), dietary energy intake (kcal, quartiles), urinary Na/K ratio and uric acid (mg/dL).
Trend p values were adjusted by FDR(False Discovery Rate) in each qurtile (123 penalties per analysis).
