# Supplementary material for: A population-based urinary and plasma metabolomics study of environmental exposure to cadmium
Source: Environ Health Prev Med. 2024 Mar 30;29:22. doi: 10.1265/ehpm.23-00218 (PMC10992994; doi:10.1265/ehpm.23-00218)
Supplement: Supplementary file 28 — Additional file 28: S Figure 28 Association between urine metabolites and cadmium exposure groups excluding drinkers in women. [file ehpm-29-022-s028.pdf]

S Figure 28 Association between urine metabolites and cadmium exposure groups excluding drinkers in women.

Forest plot in multivariate regression model (adjusted model)

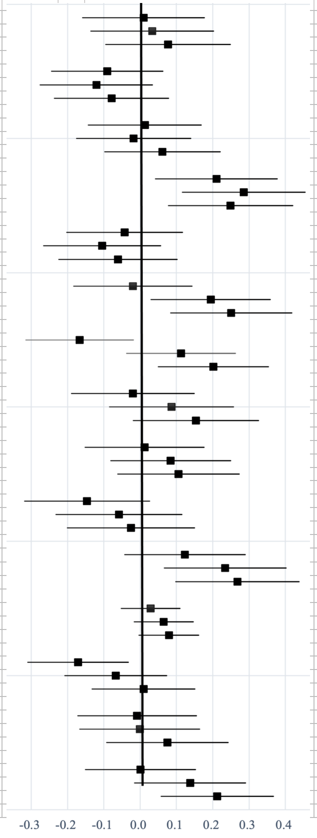

Metabolites	Cd Exposure	Standardized β	95%CI		trend FDR p
Trimethylamine N-oxide	Q2	0.009	-0.159	0.177	
	Q3	0.033	-0.136	0.203	2.01E-01
	Q4	0.077	-0.095	0.248	
Ala	Q2	-0.091	-0.244	0.063	
	Q3	-0.121	-0.276	0.035	6.79E-01
	Q4	-0.079	-0.236	0.078	
Choline	Q2	0.013	-0.143	0.168	
	Q3	-0.018	-0.175	0.139	1.11E-01
	Q4	0.061	-0.098	0.221	
Taurine	Q2	0.210	0.042	0.378	
	Q3	0.285	0.116	0.455	2.93E-03
	Q4	0.249	0.078	0.421	
1-Methylnicotinamide	Q2	-0.043	-0.202	0.117	
	Q3	-0.105	-0.266	0.056	9.38E-01
	Q4	-0.061	-0.224	0.102	
ADMA	Q2	-0.020	-0.184	0.143	
	Q3	0.194	0.029	0.359	1.17E-03
	Q4	0.251	0.084	0.418	
Uridine	Q2	-0.167	-0.315	-0.019	
	Q3	0.113	-0.037	0.262	3.67E-03
	Q4	0.202	0.050	0.354	
Adenosine	Q2	-0.020	-0.189	0.150	
	Q3	0.087	-0.084	0.257	3.62E-02
	Q4	0.154	-0.019	0.326	
Ethanolamine	Q2	0.012	-0.151	0.176	
	Q3	0.084	-0.081	0.249	2.42E-01
	Q4	0.106	-0.062	0.273	
Piperidine	Q2	-0.146	-0.318	0.026	
	Q3	-0.059	-0.232	0.115	7.98E-01
	Q4	-0.025	-0.201	0.150	
5-Aminolevulinate	Q2	0.124	-0.043	0.290	
	Q3	0.234	0.066	0.402	1.54E-04
	Q4	0.268	0.098	0.438	
Allantoin	Q2	0.029	-0.052	0.110	
	Q3	0.065	-0.017	0.147	2.10E-02
	Q4	0.079	-0.004	0.162	
7-Methylguanine	Q2	-0.171	-0.309	-0.032	
	Q3	-0.067	-0.207	0.073	8.36E-03
	Q4	0.009	-0.133	0.151	
N1-Acetylspermidine	Q2	-0.008	-0.172	0.155	
	Q3	-0.001	-0.166	0.164	1.88E-01
	Q4	0.075	-0.092	0.242	
N8-Acetylspermidine	Q2	0.001	-0.151	0.152	
	Q3	0.138	-0.015	0.291	8.78E-05
	Q4	0.212	0.057	0.368	

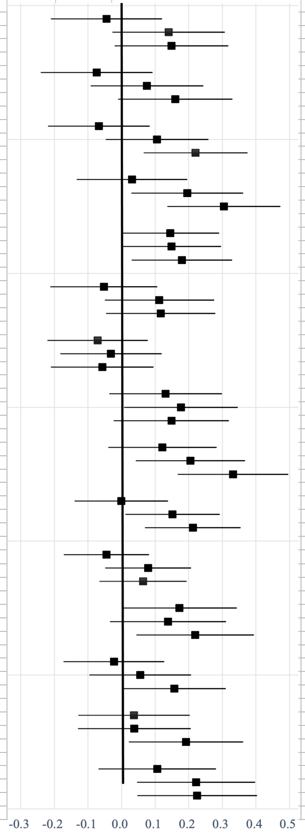

Metabolites	Cd Exposure	Standardized β	95%CI		trend FDR p
Gly-Leu	Q2	-0.045	-0.210	0.119	
	Q3	0.140	-0.026	0.306	2.84E-03
	Q4	0.149	-0.019	0.317	
N-epsilon-Acetyllysine	Q2	-0.074	-0.239	0.092	
	Q3	0.076	-0.091	0.243	3.51E-03
	Q4	0.160	-0.009	0.329	
SAM+	Q2	-0.067	-0.217	0.083	
	Q3	0.106	-0.046	0.257	4.60E-05
	Q4	0.221	0.067	0.374	
1-Methyladenosine	Q2	0.031	-0.132	0.195	
	Q3	0.196	0.031	0.362	1.08E-03
	Q4	0.305	0.137	0.472	
Lactate	Q2	0.145	0.000	0.290	
	Q3	0.149	0.003	0.295	5.27E-02
	Q4	0.180	0.032	0.328	
Malonate	Q2	-0.052	-0.211	0.106	
	Q3	0.112	-0.048	0.271	7.68E-03
	Q4	0.117	-0.045	0.279	
4-Oxopentanoate	Q2	-0.071	-0.220	0.077	
	Q3	-0.031	-0.181	0.119	7.44E-01
	Q4	-0.057	-0.209	0.094	
Succinate	Q2	0.131	-0.036	0.298	
	Q3	0.177	0.008	0.345	9.96E-03
	Q4	0.149	-0.022	0.319	
Isethionate	Q2	0.122	-0.038	0.282	
	Q3	0.205	0.044	0.367	1.67E-03
	Q4	0.332	0.169	0.496	
5-Oxoproline	Q2	0.000	-0.139	0.138	
	Q3	0.152	0.012	0.292	1.78E-01
	Q4	0.212	0.071	0.354	
Glutarate	Q2	-0.045	-0.171	0.081	
	Q3	0.079	-0.048	0.206	4.73E-01
	Q4	0.064	-0.064	0.193	
Malate	Q2	0.173	0.002	0.343	
	Q3	0.138	-0.033	0.310	8.72E-03
	Q4	0.219	0.045	0.393	
Threonate	Q2	-0.023	-0.171	0.126	
	Q3	0.056	-0.095	0.206	1.67E-02
	Q4	0.157	0.005	0.310	
Ethanolamine phosphate	Q2	0.037	-0.128	0.203	
	Q3	0.038	-0.129	0.205	1.07E-02
	Q4	0.192	0.023	0.361	
2-Oxoglutarate	Q2	0.106	-0.067	0.280	
	Q3	0.222	0.047	0.397	5.23E-03
	Q4	0.225	0.048	0.402	

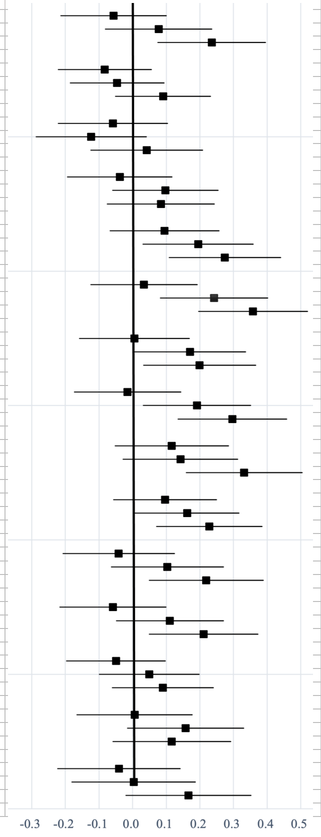

Metabolites	Cd Exposure	Standardized β	95%CI		trend FDR p
Pimelate	Q2	-0.057	-0.214	0.100	
	Q3	0.077	-0.081	0.236	1.21E-03
	Q4	0.235	0.075	0.395	
Urate	Q2	-0.083	-0.221	0.056	
	Q3	-0.046	-0.186	0.093	4.85E-04
	Q4	0.091	-0.051	0.232	
Glycerophosphate	Q2	-0.059	-0.221	0.104	
	Q3	-0.123	-0.287	0.041	4.66E-01
	Q4	0.042	-0.124	0.208	
trans-Aconitate	Q2	-0.038	-0.194	0.117	
	Q3	0.097	-0.060	0.254	3.24E-01
	Q4	0.084	-0.075	0.243	
cis-Aconitate	Q2	0.095	-0.068	0.257	
	Q3	0.195	0.031	0.359	1.43E-03
	Q4	0.274	0.108	0.440	
N-Acetylaspartate	Q2	0.034	-0.125	0.192	
	Q3	0.242	0.082	0.402	5.70E-07
	Q4	0.358	0.196	0.520	
Azelate	Q2	0.005	-0.158	0.169	
	Q3	0.171	0.006	0.336	1.13E-02
	Q4	0.199	0.032	0.366	
Isocitrate	Q2	-0.015	-0.173	0.143	
	Q3	0.191	0.032	0.351	1.38E-05
	Q4	0.297	0.135	0.458	
Adipate	Q2	0.116	-0.053	0.285	
	Q3	0.142	-0.028	0.313	4.15E-04
	Q4	0.332	0.159	0.504	
3-Hydroxy-3-methylglutarate	Q2	0.096	-0.057	0.249	
	Q3	0.162	0.007	0.317	3.77E-04
	Q4	0.228	0.071	0.384	
2,3-Pyridinedicarboxylate	Q2	-0.041	-0.207	0.124	
	Q3	0.103	-0.064	0.271	4.52E-03
	Q4	0.219	0.050	0.388	
Suberate	Q2	-0.059	-0.216	0.099	
	Q3	0.111	-0.049	0.270	6.74E-03
	Q4	0.211	0.050	0.372	
Gluconate	Q2	-0.049	-0.197	0.098	
	Q3	0.049	-0.099	0.198	1.08E-01
	Q4	0.090	-0.060	0.240	
Saccharate	Q2	0.006	-0.166	0.177	
	Q3	0.158	-0.016	0.331	1.21E-01
	Q4	0.116	-0.059	0.292	
N-Acetylneuraminate	Q2	-0.041	-0.223	0.141	
	Q3	0.003	-0.181	0.187	9.18E-02
	Q4	0.166	-0.020	0.352	

Multivariate regression model was adjusted by age (years),sex ,systolic blood pressure (mmHg), smoke (pack-year), rice intake(bowl/week), BMI (kg/m2), HbA1c (%), LDLc (mg/dL), alcohol consumption (ethanol intake: g/day), physical activity (METs, quartiles), educational history (less than 10 years, 10 to 12 years, more than 12 years), dietary energy intake (kcal, quartiles), urinary Na/K ratio and uric acid (mg/dL).
Trend p values were adjusted by FDR(False Discovery Rate) in each qurtile (123 penalties per analysis).
