# Supplementary material for: A population-based urinary and plasma metabolomics study of environmental exposure to cadmium
Source: Environ Health Prev Med. 2024 Mar 30;29:22. doi: 10.1265/ehpm.23-00218 (PMC10992994; doi:10.1265/ehpm.23-00218)
Supplement: Supplementary file 30 — Additional file 30: S Figure 30 Association between urine metabolites and cadmium exposure groups excluding drinkers in sex stratification. [file ehpm-29-022-s030.pdf]

S Figure 30 Association between plasma metabolites and cadmium exposure groups excluding drinkers in sex stratification.

Men Variables

Forest plot in univariate regression model (adjusted model)

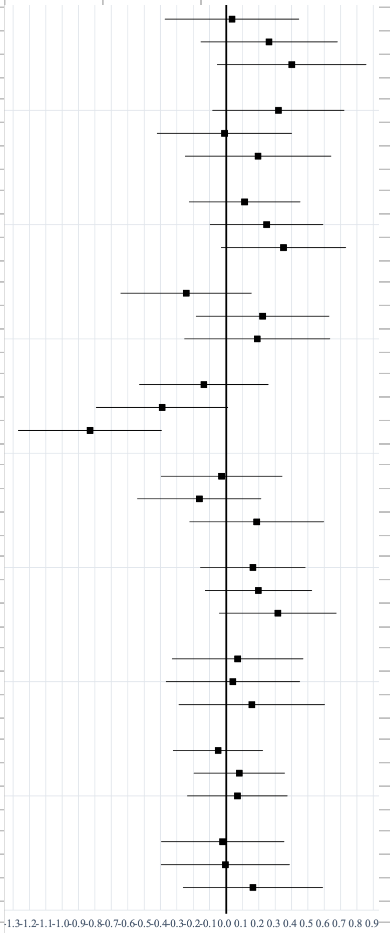

Metabolites	Cd Exposure	Standardized β	95%CI		trend FDR p
Trimethylamine N-oxi	Q2	0.036	-0.371	0.444	9.87E-03
	Q3	0.263	-0.153	0.679	
	Q4	0.401	-0.053	0.855	
Ala	Q2	0.320	-0.081	0.721	8.10E-01
	Q3	-0.009	-0.419	0.400	
	Q4	0.196	-0.249	0.641	
Sarcosine	Q2	0.114	-0.225	0.453	2.24E-01
	Q3	0.247	-0.098	0.592	
	Q4	0.351	-0.028	0.730	
N,N-Dimethylglycine	Q2	-0.243	-0.641	0.154	3.46E-02
	Q3	0.224	-0.182	0.629	
	Q4	0.190	-0.253	0.633	
Creatinine	Q2	-0.136	-0.528	0.257	2.48E-04
	Q3	-0.390	-0.790	0.011	
	Q4	-0.831	-1.266	-0.395	
Ile	Q2	-0.027	-0.395	0.342	4.62E-01
	Q3	-0.163	-0.540	0.214	
	Q4	0.187	-0.222	0.596	
Asp	Q2	0.164	-0.155	0.483	6.37E-02
	Q3	0.198	-0.127	0.522	
	Q4	0.316	-0.040	0.673	
Carnitine	Q2	0.071	-0.328	0.470	4.75E-01
	Q3	0.042	-0.366	0.449	
	Q4	0.157	-0.286	0.601	
Uridine	Q2	-0.049	-0.321	0.223	1.31E-01
	Q3	0.080	-0.196	0.357	
	Q4	0.070	-0.234	0.374	
2-Oxoglutarate	Q2	-0.020	-0.394	0.354	6.57E-01
	Q3	-0.005	-0.396	0.387	
	Q4	0.165	-0.261	0.590	

Women Variables

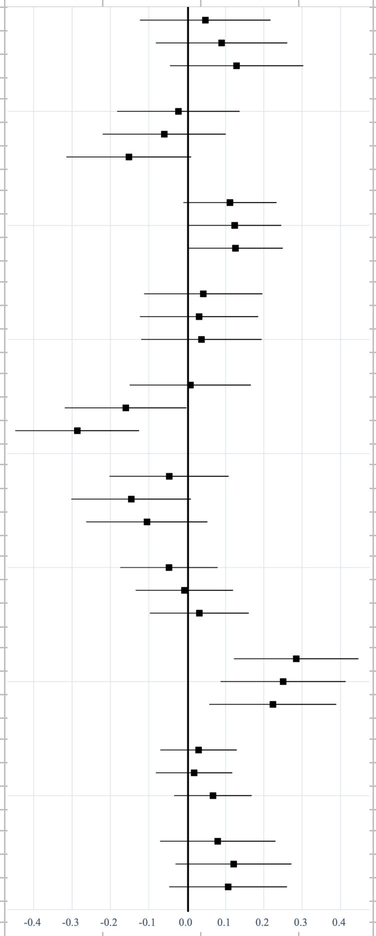

Metabolites	Cd Exposure	Standardized β	95%CI		trend FDR p
Trimethylamine	Q2	0.047	-0.122	0.217	8.56E-02
	Q3	0.090	-0.080	0.261	
	Q4	0.129	-0.044	0.302	
Ala	Q2	-0.023	-0.182	0.136	5.95E-02
	Q3	-0.060	-0.219	0.100	
	Q4	-0.152	-0.314	0.010	
Sarcosine	Q2	0.112	-0.009	0.233	3.58E-02
	Q3	0.124	0.003	0.245	
	Q4	0.126	0.003	0.249	
N,N-Dimethylg	Q2	0.042	-0.111	0.196	1.19E-01
	Q3	0.031	-0.123	0.185	
	Q4	0.037	-0.119	0.194	
Creatinine	Q2	0.008	-0.149	0.166	2.24E-04
	Q3	-0.160	-0.318	-0.002	
	Q4	-0.286	-0.447	-0.126	
Ile	Q2	-0.047	-0.201	0.108	1.43E-01
	Q3	-0.146	-0.301	0.009	
	Q4	-0.105	-0.262	0.053	
Asp	Q2	-0.047	-0.174	0.079	3.49E-02
	Q3	-0.007	-0.134	0.119	
	Q4	0.032	-0.097	0.160	
Carnitine	Q2	0.285	0.123	0.447	3.44E-02
	Q3	0.250	0.088	0.413	
	Q4	0.224	0.058	0.389	
Uridine	Q2	0.030	-0.069	0.129	5.04E-03
	Q3	0.018	-0.081	0.117	
	Q4	0.068	-0.033	0.168	
2-Oxoglutarate	Q2	0.080	-0.070	0.230	5.48E-02
	Q3	0.121	-0.029	0.272	
	Q4	0.107	-0.046	0.260	

Multivariate regression model was adjusted by age (years), sex, systolic blood pressure (mmHg), smoke (pack-year), rice intake(bowl/week), BMI (kg/m2), HbA1c (%), LDLc (mg/dL), alcohol consumption (ethanol intake: g/day), physical activity (METs, quartiles), educational history (less than 10 years, 10 to 12 years, more than 12 years), dietary energy intake (kcal, quartiles), urinary Na/K ratio and uric acid (mg/dL).

P values were adjusted by FDR(False Discovery Rate) in each qurtile (94 penalties per analysis).
